# Chemical Synthesis of Fucosylated Chondroitin Sulfate Tetrasaccharide with Fucosyl Branch at the 6-OH of GalNAc Residue

**DOI:** 10.3390/md22040184

**Published:** 2024-04-19

**Authors:** Changlun Lv, Xiaona Li, Guoqing Yang, Haijiao Chen, Chunxia Li

**Affiliations:** 1 Key Laboratory of Marine Drugs, Ministry of Education, Shandong Key Laboratory of Glycoscience and Glycotechnology, School of Medicine and Pharmacy, Ocean University of China, Qingdao 266003, China; 2 Laboratory of Marine Drugs and Bioproducts of Qingdao Pilot National Laboratory for Marine Science and Technology, Qingdao 266237, China

**Keywords:** fucosylated chondroitin sulfate, tetrasaccharide, synthesis, fucosyl branch

## Abstract

Fucosylated chondroitin sulfate is a unique glycosaminoglycan isolated from sea cucumbers, with excellent anticoagulant activity. The fucosyl branch in FCS is generally located at the 3-OH of *D*-glucuronic acid but, recently, a novel structure with α-*L*-fucose linked to the 6-OH of *N*-acetyl-galactosamine has been found. Here, using functionalized monosaccharide building blocks, we prepared novel FCS tetrasaccharides with fucosyl branches both at the 6-OH of GalNAc and 3-OH of GlcA. In the synthesis, the protective group strategy of selective *O*-sulfation, as well as stereoselective glycosylation, was established, which enabled the efficient synthesis of the specific tetrasaccharide compounds. This research enriches knowledge on the structural types of FCS oligosaccharides and facilitates the exploration of the structure–activity relationship in the future.

## 1. Introduction

**Figure 1:**
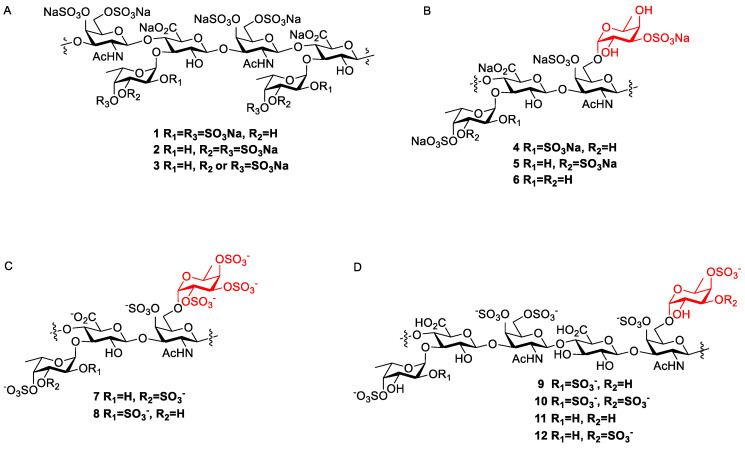
Structures of Fucosylated Chondroitin Sulfate. The typical structures of FCS (**A**), and the structures of FCS from the sea cucumber *Actinopyga mauritiana* [4] (**B**), *Cucumaria frondose* [5] (**C**), and *Holothuria mexicana* [6] (**D**).

Fucosylated chondroitin sulfate (FCS or FuCS) is a unique glycosaminoglycan found in the body wall of sea cucumbers. It mainly consists of a chondroitin sulfate (CS)-like backbone composed of *N*-acetyl-galactosamine (GalNAc), *D*-glucuronic acid (GlcA), and sulfated fucose, which is usually attached to the 3-OH of glucuronic acid, via an α-(1→3) glycosidic bond, to form the side chain of FCS. The sulfation sites of fucose in FCS mainly include 2,4-OH sulfation, 3,4-OH sulfation, and a small amount of monosulfation (3-OH or 4-OH) (Figure 1A) [1,2,3]. 

In recent years, several groups identified some new structurally unique fucosylated chondroitin sulfate polysaccharides. In 2016, Anatolii I. Usova’s group isolated fucosylated chondroitin sulfate polysaccharides from the sea cucumber *Actinopyga mauritiana*. This polysaccharide contained not only the FCS core disaccharide unit →3)-β-D-GalNAc-(1→4)-β-D-GlcA-(1→, but also a Fuc3S residue attached to 6-OH of GalNAc [4] (Figure 1B). In 2017, this group isolated another novel FCS polysaccharide, from the sea cucumber *Cucumaria frondose* (CF), with three types of fucosylated branches, which were identified as α-L-Fuc3S4S or α-L-Fuc2S4S linked to the 3-OH of the GlcNAc residue, and α-L-Fuc2S3S4S linked to the 6-OH of GalNAc [5] (Figure 1C). In 2018, Yu et al. found α-L-Fuc-4S or α-L-Fuc-3S4S were linked to the O-6 position of the GalNAc of FCS obtained from the sea cucumber *Holothuria mexicana* (FCShm) [6] (Figure 1D). These unique structures of FCS usually contain a fucose residue at the 6-OH of *D*-galactosamine.

FCS and its oligosaccharides possess a variety of pharmacological properties, including anti-inflammatory [7], antitumor [8], anti-hyperglycemic actions [9], and antiviral [10] sas well as regulating immunity and cell proliferation [11]. It has aroused a lot of interest because of its notable antithrombotic and anticoagulant activities [12]. FCS oligosaccharides can selectively inhibit intrinsic tenase (FXase, factor IXa-VIIIa complex) in the endogenous coagulation pathway [13] with low bleeding risk. Thus, it has high potential to be developed as a novel anticoagulant and antithrombotic drug candidate [13].

Due to the complexity of the FCS polysaccharide structure, it is impossible to elucidate the structure–activity relationship and accurately identify the core fragment responsible for biological activity. Therefore, various methods are employed to obtain homogeneous FCS oligosaccharides. In 2023, Jinhua Zhao’s group [14] employed copper ion-catalyzed peroxidative depolymerization [15] and β-eliminative depolymerization [16] methods to obtain fourteen FCS oligosaccharides from the sea cucumber *Phyllophorella kohkutiensis* (PkFCS), and they found that octasaccharide (Pk4b) with sulfated fucose-based side chains was the smallest fragment responsible for its anticoagulant activity associated with anti-FXase [14]. In addition, several research groups employed chemical synthesis methods to produce FCS oligosaccharides with well-defined diverse and flexible structures.

Jun-ichi Tamura et al. prepared FCS trisaccharide β-D-GalNAc(4,6-diS)(1–4)[α-L-Fuc(2,4-diS)(1–3)]-β-D-GlcA through chemical synthesis [17]. In 2018, Zhongjun Li et al. synthesized FuCS hexasaccharides and nonasaccharides using a 12-step linear procedure using the chemoenzymatic method [12], and the one-step coupling of fucose to the main chain greatly reduced the reaction steps. In 2019, Hongbo Qin et al. used a stepwise synthetic method to synthesize the trisaccharide repeating unit [α-L-Fuc(2,4-diS)(1→3)]-β-D-GlcA(1→3)-β-D-GalNAc(4,6-diS) of FCS [18]. In 2020, Biao Yu’s group reported the use of orthogonally protected trisaccharides as the key building blocks to construct hexasaccharides and nonasaccharides with [3+3] and [3+6] glycosylation strategies [19]. It is worth noting that the fucose of these FCS oligosaccharides was attached to the 3-OH of glucuronic acid, while fucosyl branches at the 6-OH of GalNAc have not been synthesized yet.

**Scheme 1:**
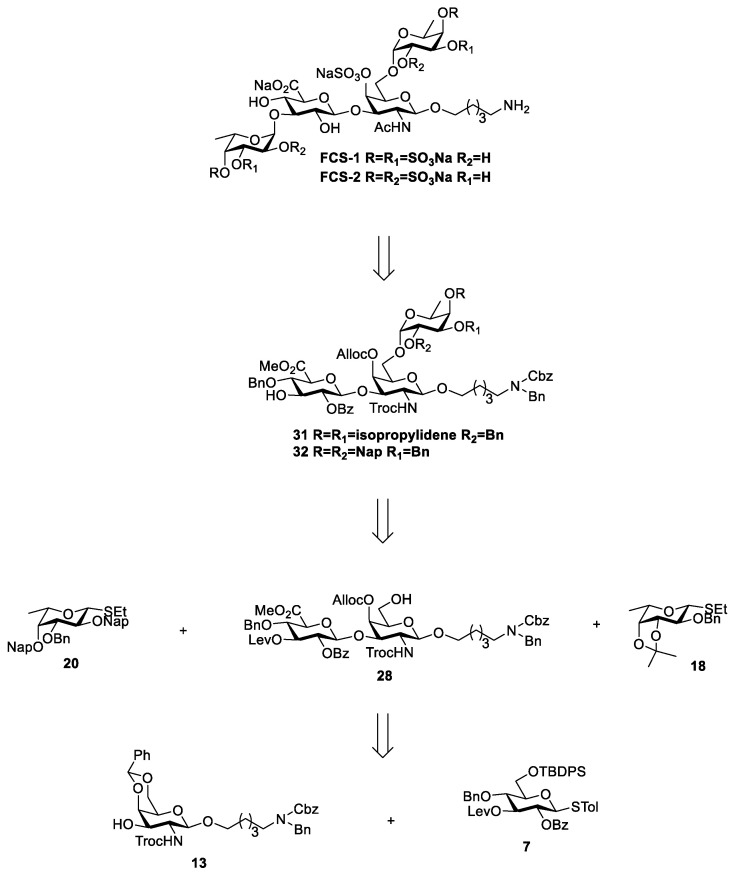
Retrosynthetic analysis of tetrasaccharides of **FCS-1** and **FCS-2**.

Herein, we report the synthesis of two FCS tetrasaccharide compounds with fucosyl branches both at the 6-OH of GalNAc and the 3-OH of GlcA (**FCS-1**, **2**, Scheme 1), based on the FCS structural types isolated from the sea cucumbers *Actinopyga mauritiana* [4] and *Cucumaria frondose* [5].

## 2. Results and Discussion

For **FCS-1** and **2**, the main backbone is formed of *D*-glucuronic acid and *N*-acetyl-galactose through β-(1→3) glycosidic bonds, with *O*-sulfated fucosyl residues attached to glucuronic acid and *N*-acetylgalactosamine via α-(1→3) and α-(1→6) glycosidic bonds, respectively. Sulfate groups were present on *N*-acetyl-galactosamine and fucose residues. The retrosynthetic analysis of **FCS-1** and **2** is shown in Scheme 1. Three commercially available monosaccharides, *N*-acetyl-galactosamine (GalNAc), *D*-glucuronic acid (GlcA), and fucose (Fuc), were used as starting materials to obtain functionalized monosaccharide building blocks **7**, **13**, **18**, and **20** by protecting group manipulation, and then to construct the CS-like disaccharide backbone through glycosylation. Finally, two fucose building blocks were introduced to afford the tetrasaccharide compounds **FCS-1** and **FCS-2** with 2,4- or 3,4-sulfation of fucose residue.

Selective *O*-sulfation and stereoselective control of glycosidic bonds were achieved using the orthogonal protection strategy. The sulfation sites in fucose building blocks (**18**, **20**) were selectively protected with isopropylidene and 2-naphthyl methyl ether (Nap), respectively. The isopropylidene could be removed under mild acidic conditions while the Nap could be oxidatively cleaved using 2,3-dichloro-5,6-dicyanobenzoquinone (DDQ) in DCM/H_2_O. The sulfation site 4-OH in GalNAc was protected with allyloxycarbonyl (Alloc) group. For building block **7** of GlcA, a benzoyl group (Bz) was introduced at the 2-OH of glucose to construct a 1,2-*trans*-glycosidic bond through neighbouring group participation, and levulinoyl (Lev) was introduced at the 3-OH as a temporary protecting group to facilitate the removal for coupling with fucose. Finally, amino-linker was introduced at the reducing end of the compound.

**Scheme 2:**
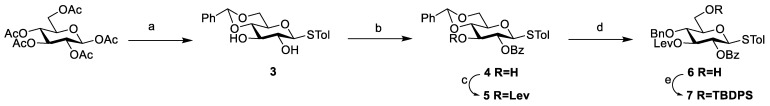
Preparation of glucose monosaccharide block **7**. Reagents and conditions: (a): i. BF_3_·Et_2_O, TolSH, DCM, 6 h; ii. CH_3_ONa, CH_3_OH; iii. CSA, PhCH(OCH_3_)_2_, CH_3_CN, 10 h, 75% for three steps; (b): Ag_2_O, BzCl, CHCl_3_, 63%; (c): LevOH, EDCI, DMAP, DCM, 86%; (d): BH_3_·THF, TMSOTf, DCM, overnight, 80%; (e): TBDPSCl, DMAP, Py, 9 h, 89%.

Based on the structural features of the target compounds and retrosynthetic analysis (Scheme 1), we firstly synthesized the monosaccharide building blocks of *N*-acetylaminogalactose (GalNAc), *D*-glucuronic acid (GlcA), and *L*-fucose (Fuc) (compounds **7**, **13**, **18**, and **20**). Using peracetylated glucose as the starting material, a seven-step reaction procedure was carried out to obtain glucosyl thioglycoside donor **7** (Scheme 2). Peracetylated glucoside was converted to β-thioglycoside in the presence of boron trifluoride diethyl etherate (BF_3_·Et_2_O) and *p*-tolylthiophenol, followed by de-acetylation, and then the 4,6-O-benzylidene glucoside **3** [18] was obtained in the presence of camphorsulphonic acid and benzaldehyde dimethyl acetal with 75% yield for three steps. The glucoside **3** was selectively benzoylated at 2-OH with 63% yield using the Ag_2_O-mediated site-selective benzoylation method [20], and then Lev group was introduced at the C-3 hydroxyl to give compound **5** [21]. Finally, the 4,6-O-benzylidene was selectively cleaved under the condition of trimethylsilyl trifluoromethanesulfonate (TMSOTf) and borane-tetrahydrofuran complex (BH_3_·THF), and the exposed C-6 hydroxyl group was protected with tert-butyldiphenylsilyl group (TBDPS) to give glucosyl thioglycoside donor **7** [22] in 89% yield.

**Scheme 3:**
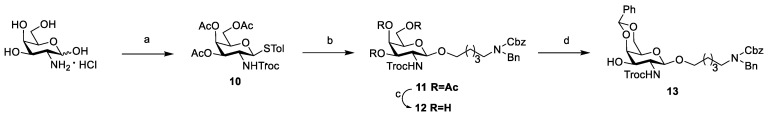
Preparation of aminogalactose monosaccharide block **13**. Reactions and conditions: (a): i. TrocCl, NaHCO_3_, H_2_O; ii. Ac_2_O, DMAP, Py; iii. BF_3_·Et_2_O, TolSH, DCM, 71% for three steps; (b): **L-1**, TMSOTf, NIS, DCM, −30 °C, 1 h, 91%; (c): CH_3_ONa, CH_3_OH, 0 °C, 30 min, quantitative; (d): CH_3_CN, CSA, PhCH(OCH_3_)_2_, 90%.

In order to construct a 1,2-*trans*-β-glycosidic bond of 2-deoxy-β-D-galactopyranoside, phthaloyl (Phth) and trichloroethoxycarbonyl (Troc), through neighbouring group participation, are often used as amino-protecting groups [23]. The conditions for the removal of Phth are more drastic, and reduce the activity of the glycosyl donor as an acyl-protecting group. On the other hand, the conditions for introducing and removing the Troc group are relatively milder, and enhance the activity of the Glycosyl donor. Therefore, Troc was chosen as an amino-protecting group for *N*-acetylaminogalactose. The compound **10** was obtained in a three-step reaction with aminogalactose hydrochloride as the starting material in 71% yield [23]. Subsequently, **10** was coupled with *N*-benzyl-benzylcarbamate protected aminopentyl linker **L-1** [24] under *N*-iodosuccinimide (NIS) and TMSOTf conditions to give β-linked product **11** [25] in 91% yield. Then the acetyl groups were removed and a benzylidene group was formed between the C-4/C-6 hydroxyl groups to give the 3-OH unprotected aminogalactose acceptor **13** [25] (Scheme 3).

**Scheme 4:**
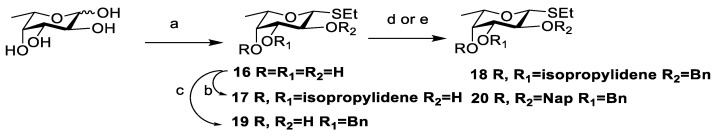
Preparation of fucose monosaccharide blocks **18** and **20**. Reactions and conditions: (a): i. DMAP, Ac_2_O, Py, quantitative; ii. BF_3_·Et_2_O, EtSH, DCM, 95% (α/β = 1/7); iii. CH_3_ONa, CH_3_OH, quantitative; (b): CSA, 2,2-DMP, acetone, 84%; (c): i. Bu_2_SnO, 7 h, toluene; ii. CsF, BnBr, DMF, 87%; (d): NaH, BnBr, DMF, 87% from **17** to **18**; (e): NaH, NapBr, DMF, 73% from **19** to **20**.

In order to synthesize FCS tetrasaccharides with different sulfation modes in Fuc, two different fucose thioglycosides, **18** and **20**, were designed (Scheme 4). The compound **18** was obtained through a 5-step reaction, according to the reported methods, in 69% yield [26,27]. For thioglycoside **20**, we first utilized dibutyltin oxide (Bu_2_SnO) to selectively introduce a Bn group at the 3-OH of compound **16** [28]. Then C-2 and C-4 hydroxyls were protected with Nap to afford the fucose thioglycoside donor **20**.

**Scheme 5:**
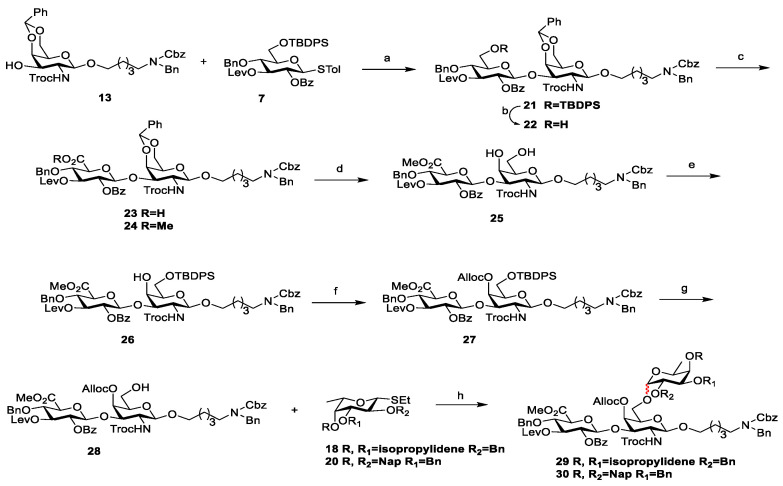
Preparation of trisaccharide compounds **29** and **30**. Reactions and conditions: (a): NIS, TfOH, DCM, −25 °C, 20 min, 94%; (b): HF·Py, THF, Py, 7 h, 85%; (c): i. TEMPO, BAIB, DCM, H_2_O, 6 h; ii. K_2_CO_3_, CH_3_I, DMF, 10 h, 74% for two steps; (d): AcOH, H_2_O, DCM, 6 h, 89%; (e): TBDPSCl, DMAP, Py, 5 h, 88%; (f): AllocCl, DMAP, 86%; (g): HF·Py, THF, Py, 2 h; (h): NIS, TfOH, DCM, Et_2_O, −15 °C, 30 min, 90% for two steps (**29**, α:β = 1.8:1), 92% for two steps (**30**, α:β = 2.75:1).

After obtaining the three kinds of monosaccharide blocks, the synthesis of chondroitin sulfate disaccharide blocks was carried out (Scheme 5). Under the condition of NIS and trifluoromethanesulfonic acid (TfOH), the aminogalactose acceptor **13** (1 eq) and glucose donor **7** (1.3 eq) were coupled at −25 °C. The glycosylation reaction was performed in a more favorable yield of 94%, and only β-linked product **21** was found (*J*_1,2_ = 7.9 Hz).

According to the retrosynthetic analysis, the differentiation of the C-4 and C-6 hydroxyl groups of GalNAc and the conversion of glucose to glucuronic acid need to be accomplished in the disaccharide block. The 6-OTBDPS of glucoside **21** was removed to give disaccharide compound **22** in 85% yield. Selective oxidation of the primary hydroxyl group in glucose unit under 2,2,6,6-Tetramethylpiperidinooxy (TEMPO) and (diacetoxyiodo) benzene (BAIB) conditions and then methyl ester protection afforded compound **24** in 74% yield for two steps [29]. Then the 4,6-O-benzylidene group of GalNAc in compound **24** was removed under acetic acid (AcOH) conditions to afford compound **25** in 89% yield. Finally, selective protection of the C-6 OH with TBDPS and the C-4 OH by Alloc gave compound **27** in two-step yield of 76%. Next, the trisaccharide backbone was constructed by removing the 6-TBDPS of GalNAc and coupling with fucose blocks, as shown in Scheme 5.

Then, compound **27** was stripped of the TBDPS protecting group to give **28** for glycosylation. Unfortunately, when compound **28** was coupled with fucose donor **18** catalyzed by NIS and TfOH, the glycosylation product was α/β-mixture (Entry 1) and the α-linked trisaccharide **29** was isolated in 41% yield (H_1_ = 4.79 ppm, C_1_ = 97.9 ppm for fucosyl unit) with a β-isomer of 37%. In order to improve the stereoselectivity, we optimized this glycosylation condition in terms of catalyst type, dosage and reaction temperature, and the experimental results are shown in Table 1.

According to Table 1, the stereoselectivity was poor (α:β = 1.1:1, Entry 1) at reaction temperatures of −25 °C. When the temperature was cooled to −45 °C, the stereoselectivity was improved (α:β = 1.25:1, Entry 2) but the yield was low (62%). Surprisingly, when the glycosylation temperature raised to −15 °C, the ratio and yield of α-linked trisaccharide increased (α:β = 1.3:1, Entry 3). On the other hand, the use of a higher amount of fucose donor 18 can increase α-selectivity (α:β = 1.8:1, Entry 4). Then, different Lewis acid catalysts such as TMSOTf or TBSOTf were tried, with no improvement in stereoselectivity (Entry 5, 6). So, the condition of Entry 4 was selected to prepare trisaccharide **29** with high α-selectivity (α:β = 1.8:1). This glycosylation reaction can be completed within 30 min and the α-, β-isomers can be easily separated using silica gel chromatography. Then, the subsequent reaction was continued with the pure alpha anomer of compound **29** (Figure S13).

Using the same glycosylation conditions, trisaccharide **30** was obtained using the disaccharide acceptor **28** with another fucose donor **20**. The α/β ratio was 2.75:1. After purification, the α-linked trisaccharide compound **30** was obtained in two steps with 67% yield (Figure S14). Comparing the stereoselectivity of two glycosylation products (**29**, **30**), it was found that the 2-Nap-protected fucose donor **20** was superior to that of the isopropylidene-protected fucose donor **18**, which might be due to the spatial effect of 2-Nap.

**Scheme 6:**
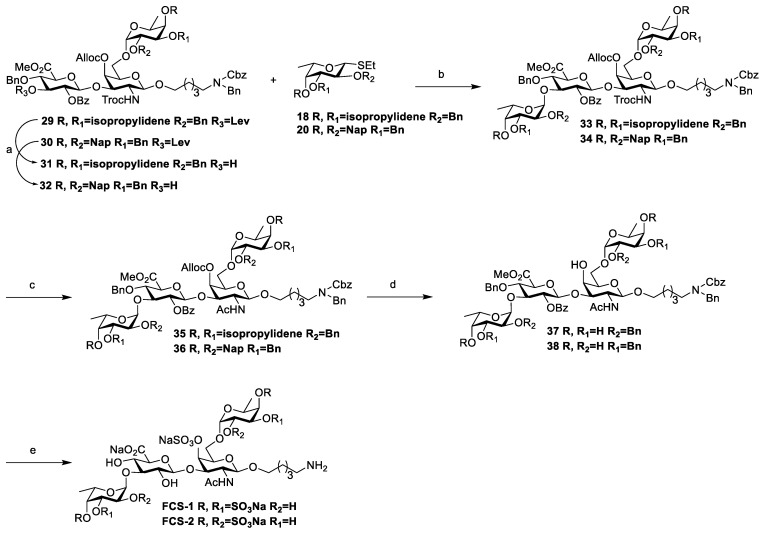
Preparation of tetrasaccharide compounds **FCS-1** and **FCS-2**. Reactions and conditions: (a): NH_2_NH_3_OAc, DCM, CH_3_OH, 5 h, 87% for **31**, 97% for **32**; (b): NIS, TfOH, DCM, Et_2_O, **18** or **20**, −15 °C, 72% for **33**, 83% for **34**; (c): Zn, THF:Ac_2_O:AcOH = 6:1:1, 2 h, 80% for **35**, 74% for **36**; (d): (1) i. AcOH, H_2_O, 11 h; ii. PPh_3_, Pd(PPh_3_)_4_, Ammonium formate, THF, 8 h, two steps, 78% for **37**; (2) i. DDQ, H_2_O, PBS, 1 h; ii. PPh_3_, Pd(PPh_3_)_4_, Ammonium formate, THF, 8 h, two steps, 60% for **38**; (e): i. SO_3_·Me_3_N, DMF, 70 °C for 120 min, then 80 °C for 40 min; ii. LiOH, NaOH, THF, H_2_O; iii. Pd(OH)_2_/C, Pd/C, CH_3_OH, H_2_O, three steps, 63% for **FCS-1**, 65% for **FCS-2**.

Next, the assembly of the tetrasaccharide compounds was performed (Scheme 6). Under the condition of hydrazine acetate, the Lev at the C-3 of **29** and **30** was removed to afford the two trisaccharide acceptors **31** and **32**. Subsequently, fucose donors **18** and **20** were coupled with **31** and **32,** respectively, under the glycosylation conditions described above. To our surprise, only the α-linked tetrasaccharide products **33** and **34** (*J*_1‴,2‴_ = 3.3 Hz) were found (Figure S15), while the β-isomer was not detected.

In the fucosylation reaction, the same fucose donor showed better glycosylation stereoselectivity with GlcA 3-OH than with GalNAc 6-OH. We hypothesize that the difference in reactivity between the primary and secondary hydroxyl groups of the acceptor may be a contributing factor. Additionally, spatial orientation likely influences the formation of the α-isomer during the coupling of the fucose donor with GlcA 3-OH.

Due to the strong electron-withdrawing nature of the carboxyl group in glucuronic acid, the glycosylation reaction was inadequate under the same glycosylation conditions (*N*-iodosuccinimide (1.5 eq), TfOH (0.3 eq), 4 Å MS, CH_2_Cl_2_, −15 °C), resulting in low yields, which were 59% and 51% for tetrasaccharides **34** and **33**, respectively. In order to improve the yield, the equivalent of TfOH in the glycosylation reaction was explored. It was found that increasing the TfOH equivalent could improve the yield. When the TfOH equivalent was increased from 0.3 eq to 0.6 eq, the glycosylation yield of compound **34** was increased from 51% to 83%. Similarly, the yield of compound **33** was improved from 59% to 72%.

Subsequently, a series of functional group transformations were carried out (Scheme 6). Firstly, the NHTroc in **33** and **34** was reduced to amino group using Zn powder [30] and acetylated to obtain compounds **35** and **36** in 80% and 74% yields, respectively. Then the isopropylidene at 3,4-OH and the Nap in fucose were removed, followed by removal of Alloc [18] to give pentol **37** and **38** as sulfated precursor in 78% and 60% yields, respectively.

Finally, the FCS tetrasaccharide compounds **FCS-1** and **FCS-2** were obtained with three steps in one pot. Firstly, compounds **37** and **38** were sulfated using sulfur trioxide trimethylamine complex (SO_3_·Me_3_N) in a microwave reactor [19] with DMF as the solvent to afford the corresponding sulfated tetrasaccharide derivatives; then, the methyl group of COOMe and benzoyl group were removed under alkaline conditions and, finally, the Bn and Cbz groups were removed through Pd/C-catalyzed hydrogenolysis to afford the target compounds **FCS-1** and **FCS-2** in 63% and 65% yields with three steps, respectively. **FCS-1** and **FCS-2** were identified by ^1^H-NMR, ^13^C-NMR, 2D-NMR, and HR-ESI-MS.

The FCS tetrasaccharides with fucosyl branches both at the 6-OH of GalNAc and the 3-OH of GlcA were synthesized for the first time through consecutive fucosylation using the linear synthesis method. **FCS-1** and **FCS-2** exhibit different fucose sulfation patterns, with **FCS-1** featuring 3,4-OH sulfation and **FCS-2** featuring 2,4-OH sulfation. Additionally, they both contain sulfate groups at the 4-OH of GalNAc. In the synthesis process, the 1,2-*trans*-β-glycosidic bond was successfully constructed by introducing the Troc protecting group in aminogalactose. The conversion of glucuronic acid was achieved at the disaccharide blocks, which reduced the reaction steps and established an efficient and concise synthesis strategy.

## 3. Materials and Methods

### 3.1. General Experimental Procedures

All chemicals were purchased from commercial suppliers and were used without further purification unless noted. Ether (Et_2_O), Pyridine (Py), Methanol (CH_3_OH), and Toluene (Toluene) were dried using 4Å molecular sieves. N, N-dimethylformamide (DMF), dichloromethane (DCM), and tetrahydrofuran (THF) were processed via the solvent purification system (FL-MD-3, Beijing Yifeng Science and Technology Co., Beijing, China). Crushed 4 Å molecular sieves (MS) were activated using flame drying under vacuum immediately before use. Thin-layer chromatography (TLC) was performed on silica gel plates 60 F254 (Yantai, China). Column chromatography silica gel was 200–300 mesh or 300–400 mesh (Qingdao Ocean Chemical Factory, Qingdao, China). Sulfation reaction was operated using a microwave synthesizer (CEM Discover, Matthews, NC, USA).

^1^H-NMR, H-H COSY, ^13^C-NMR, and HSQC spectra were measured using JNM-ECZ-600R/S1, Agilent DD2 500-MHz, or Bruker AVANCE NEO 400 MHz NMR spectrometers. NMR spectra were calibrated using solvent signals (CDCl_3_: ^1^H: 7.26 ppm, ^13^C: 77.16 ppm; CD_3_OD: ^1^H: 3.31 ppm, ^13^C: 49.00 ppm; D_2_O: ^1^H: 4.79 ppm). High-resolution electrospray ionization (ESI) mass spectra were measured using LTQ orbitrsp XL (Thermo Fisher, Waltham, MA, USA).

### 3.2. Chemical Synthesis

Compounds **7** [22], **13** [25], and **18** [26,27] were synthesized according to Scheme 2, Scheme 3, and Scheme 4, respectively. Their NMR data were found to be consistent with the literature.

Compound **21**: To a solution of glucose donor **7** [22] (141 mg, 0.17 mmol) and acceptor **13** [25] (100 mg, 0.13 mmol) in dry DCM (3 mL) dried 4 Å molecular sieves were added under a nitrogen atmosphere at room temperature. The mixture was stirred at room temperature for 1 h and then cooled to −25 °C. NIS (59 mg, 0.26 mmol) and TfOH (8.7 µL, 0.09 mmol) were added to the reaction solution and stirred for 20 min. The reaction was quenched with Et_3_N and gradually warmed to room temperature. The mixture was filtered through celite and extracted with DCM. The organic phase was washed with saturated NaHCO_3_ and brine, dried with anhydrous Na_2_SO_4_, and filtered and concentrated in vacuo. The residue was purified using flash chromatography (PE/EtOAc = 10:1, *v*/*v*) to afford white solid compound **21** (180 mg, 94%), R*_f_* = 0.27 (PE/EtOAc = 2:1, *v*/*v*). ^1^H NMR (400 MHz, CDCl_3_): *δ* 8.02 (d, *J* = 4.0 Hz, 2H, Ar-*H*), 7.73–7.64 (m, 4H, Ar-*H*), 7.55 (t, *J* = 7.4 Hz, 1H, Ar-*H*), 7.50–7.06 (m, 28H, Ar-*H*), 5.42–5.33 (m, 2H, Ph-C*H*, *H-3*), 5.22 (dd, *J* = 9.6, 7.9 Hz, 1H, *H-2*), 5.19–5.08 (m, 2H, Cbz-*CH_2_*), 4.87 (d, *J* = 7.9 Hz, 1H, *Glu-H-1*), 4.82 (d, *J* = 7.1 Hz, 1H, *Gal-H-1′*), 4.70 (d, *J* = 12.3 Hz, 1H, Ph-C*H_2_*), 4.61 (d, *J* = 11.1 Hz, 1H, CCl_3_*CH_2_*), 4.52–4.41 (m, 4H, N-Ph*CH_2_*, *H-3′*, CCl_3_*CH_2_*), 4.36–4.27 (m, 2H, *H-4′*, Ph-C*H_2_*), 4.17 (d, *J* = 11.9 Hz, 1H, *H-6a′*), 4.02 (d, *J* = 9.8 Hz, 1H, *H-6a*), 3.87 (dd, *J* = 11.2, 5.7 Hz, 1H, *H-6b*), 3.80 (d, *J* = 11.1 Hz, 2H, *H-6b′*, OC*H_2_-a*), 3.72 (t, *J* = 9.4 Hz, 1H, *H-4*), 3.64–3.56 (m, 1H, *H-5*), 3.44–3.27 (m, 2H, *H-2′*, OC*H_2_-b*), 3.24 (s, 1H, *H-5′*), 3.23–3.12 (m, 2H, NC*H_2_*), 2.53–2.44 (m, 2H, Lev-C*H_2_*), 2.44–2.32 (m, 2H, Lev-C*H_2_*), 2.00 (s, 3H, Lev-C*H_3_*), 1.55–1.37 (m, 4H, OCH_2_C*H_2_*, C*H_2_*CH_2_N), 1.29–1.15 (m, 2H, OCH_2_CH_2_C*H_2_*), 1.10 (s, 9H, SiC(C*H_3_*)*_3_*). ^13^C NMR (100 MHz, CDCl_3_): *δ* 206.0, 172.0, 165.3, 154.0, 138.0, 138.0, 137.6, 135.8, 135.6, 133.6, 133.4, 133.1, 130.1, 130.0, 129.7, 128.7, 128.5, 128.2, 128.1, 128.0, 127.3, 126.4, 126.4, 101.8(C-1), 100.7(Ph-CH), 99.4(C-1′), 95.8, 76.3(C-5), 76.2(C-4′), 76.0(C-4), 75.4(C-3), 75.3(C-3′), 74.7, 74.0, 72.6(C-2), 69.8(O-CH_2_), 69.1(C-6′), 67.3(Cbz-CH_2_), 66.6(C-5′), 63.4(C-6), 54.2(C-2′), 50.4(N-PhCH_2_), 47.3(N-CH_2_), 46.2(N-CH_2_), 37.9(Lev-CH_2_), 29.7(Lev-CH_3_), 29.2, 28.1(Lev-CH_2_), 27.5, 27.1(SiC(CH_3_)_3_), 23.3, 19.6. HRMS (ESI) *m*/*z* calcd for C_77_H_86_Cl_3_N_2_O_17_Si [M+H]^+^ 1443.4756, found 1443.4731.

Compound **22**: To a solution of **21** (191 mg, 0.13 mmol) in THF/Py (3 mL/0.6 mL), HF·Py (400 μL) was added at 0 °C under a nitrogen atmosphere. After being warmed to room temperature, the mixture was stirred for 7 h. The resulting mixture was concentrated in vacuum and extracted with DCM. The organic phase was washed with 1 N HCl, saturated NaHCO_3,_ and brine, dried with anhydrous Na_2_SO_4_, and filtered and concentrated in vacuo. The residue was purified using flash chromatography (PE/EtOAc = 1:1, *v*/*v*) to afford white solid compound **22** (135 mg, 85%), R*_f_* = 0.13 (PE/EtOAc = 1:1, *v*/*v*). ^1^H NMR (400 MHz, CDCl_3_): *δ* 8.00 (d, *J* = 7.4 Hz, 2H, Ar-*H*), 7.55 (t, *J* = 7.3 Hz, 1H, Ar-*H*), 7.40 (t, *J* = 7.7 Hz, 2H, Ar-*H*), 7.37–7.19 (m, 19H, Ar-*H*), 7.15 (d, *J* = 6.1 Hz, 1H, Ar-*H*), 5.40 (s, 1H, Ph-C*H*), 5.36 (t, *J* = 9.4 Hz, 1H, *H-3*), 5.20 (t, *J* = 8.9 Hz, 1H, *H-2*), 5.17–5.12 (m, 2H, Cbz-*CH_2_*), 4.99 (d, *J* = 7.3 Hz, 1H, *Glu-H-1*), 4.82 (d, *J* = 7.8 Hz, 1H, *Gal-H-1′*), 4.73 (d, *J* = 12.3 Hz, 1H, CCl_3_*CH_2_*), 4.66 (q, *J* = 11.4 Hz, 2H, Ph-C*H_2_*), 4.56–4.41 (m, 3H, N-Ph*CH_2_*, *H-3′*), 4.27 (d, *J* = 11.4 Hz, 2H, *H-6a′*, *H-4′*), 4.21–4.08 (m, 2H, CCl_3_*CH_2_*), 4.03 (d, *J* = 12.0 Hz, 1H, *H-6b′*), 3.92–3.71 (m, 4H, *H-4*, *H-6a*, *H-6b*, OC*H_2_-a*), 3.49 (d, *J* = 9.6 Hz, 2H, *H-5*, *H-2′*), 3.42 (s, 1H, *H-5′*), 3.40–3.28 (m, 1H, OC*H_2_-b*), 3.28–3.12 (m, 2H, NC*H_2_*), 2.55–2.45 (m, 2H, Lev-C*H_2_*), 2.39–2.28 (m, 2H, Lev-C*H_2_*), 2.00 (s, 3H, Lev-C*H_3_*), 1.57–1.39 (m, 4H, OCH_2_C*H_2_*, C*H_2_*CH_2_N), 1.29–1.23 (m, 2H, OCH_2_CH_2_C*H_2_*). ^13^C NMR (100 MHz, CDCl_3_): *δ* 205.9, 172.0, 165.4, 154.4, 138.0, 137.7, 133.6, 130.1, 129.4, 129.0, 128.7, 128.7, 128.7, 128.3, 128.2, 128.2, 128.1, 128.0, 127.4, 127.3, 126.4, 101.1, 100.3, 99.8, 95.71, 75.8, 75.7, 75.1, 75.0, 74.8, 74.1, 73.8, 72.2, 69.8, 69.3, 67.3, 66.5, 61.5, 54.1, 50.4, 50.4, 47.3, 46.1, 37.8, 29.7, 28.1, 23.4. HRMS (ESI) *m*/*z* calcd for C_61_H_71_Cl_2_^37^ClN_3_O_17_ [M+NH_4_]^+^1224.3814, found: 1224.3849. 

Compound **24**: To a solution of **22** (300 mg, 0.25 mmol) in DCM/H_2_O (1 mL/0.5 mL), TEMPO (19 mg, 0.12 mmol) and BAIB (200 mg, 0.62 mmol) were added under a nitrogen atmosphere. The reaction mixture was stirred for 7 h at room temperature. The reaction was quenched with saturated Na_2_S_2_O_3_, then extracted with DCM, and washed with brine. The organic phase was dried with anhydrous Na_2_SO_4_, and filtered and concentrated in vacuo to give compound **23** as a yellow oil, which could be used in the next reaction without purification.

To a solution of **23** in dry DMF (2 mL), K_2_CO_3_ (44 mg, 0.32 mmol) and CH_3_I (69 μL, 1.11 mmol) were added at 50 °C under a nitrogen atmosphere. After being stirred for 5 h, the reaction was quenched using 1 N HCl, then extracted with EtOAc and washed with saturated Na_2_S_2_O_3,_ water, and brine. The organic phase was dried with anhydrous Na_2_SO_4_, and filtered and concentrated in vacuo. The residue was purified using flash chromatography (PE/EtOAc = 1:1, *v*/*v*) to afford white solid compound **24** (227 mg, 74% for two steps), R*_f_* = 0.84 (PE/EtOAc = 1:2, *v*/*v*). ^1^H NMR (400 MHz, CDCl_3_): *δ* 8.00 (d, *J* = 7.5 Hz, 2H, Ar-*H*), 7.58–7.46 (m, 3H, Ar-*H*), 7.42 (t, *J* = 7.6 Hz, 2H, Ar-*H*), 7.39–7.18 (m, 19H, Ar-*H*), 7.15 (d, *J* = 4.7 Hz, 1H, Ar-*H*), 5.52 (s, 1H, Ph-C*H*), 5.33 (t, *J* = 7.8 Hz, 1H, *H-3*), 5.22 (t, *J* = 7.8 Hz, 1H, *H-2*), 5.19–5.09 (m, 2H, Cbz-*CH_2_*), 4.96 (d, *J* = 6.8 Hz, 1H, *Glu-H-1*), 4.83 (d, *J* = 8.3 Hz, 1H, *Gal-H-1′*), 4.65 (d, *J* = 11.8 Hz, 1H, CCl_3_*CH_2_*), 4.57 (q, *J* = 11.3 Hz, 2H, Ph-C*H_2_*), 4.52–4.41 (m, 3H, N-Ph*CH_2_*, *H-3′*), 4.34 (d, *J* = 3.1 Hz, 1H, *H-4′*), 4.28 (d, *J* = 11.7 Hz, 1H, *H-6a′*), 4.22–4.13 (m, 1H, CCl_3_*CH_2_*), 4.10 (d, *J* = 7.0 Hz, 2H, *H-4*, *H-5*), 4.05 (d, *J* = 12.2 Hz, 1H, *H-6b′*), 3.89–3.76 (m, 1H, OC*H_2_-a*), 3.72 (s, 3H, COOC*H_3_*), 3.43 (s, 1H, *H-5′*), 3.43–3.27 (m, 2H, *H-2′*, OC*H_2_-b*), 3.26–3.11 (m, 2H, NC*H_2_*), 2.55–2.47 (m, 2H, Lev-C*H_2_*), 2.43–2.33 (m, 2H, Lev-C*H_2_*), 2.01 (s, 3H, Lev-C*H_3_*), 1.53–1.40 (m, 4H, OCH_2_C*H_2_*, C*H_2_*CH_2_N), 1.29–1.23 (m, 2H, OCH_2_CH_2_C*H_2_*). ^13^C NMR (100 MHz, CDCl_3_): *δ* 206.0, 171.8, 168.7, 165.0, 138.0, 137.6, 133.5, 130.1, 129.4, 128.9, 128.7, 128.7, 128.6, 128.6, 128.2, 128.1, 128.0, 127.4, 127.4, 126.4, 101.2, 100.8, 100.3, 99.5, 95.8, 77.4, 75.7, 74.9, 74.7, 74.2, 74.0, 74.0, 72.1, 69.8, 69.7, 69.3, 67.3, 66.6, 54.1, 52.8, 50.6, 37.9, 29.8, 29.7, 29.2, 29.1, 28.1, 23.3. HRMS (ESI) *m*/*z* calcd for C_62_H_71_Cl_2_N_3_O_18_^37^Cl [M+NH_4_]^+^1252.3763, found 1252.3799.

Compound **25**: Compound **24** (58 mg, 0.05 mmol) was dissolved in AcOH/H_2_O (2 ml/0.5 ml) and stirred for 6 h at room temperature. The reaction was quenched with saturated NaHCO_3_, then extracted with DCM and washed with brine. The organic phase was dried with anhydrous Na_2_SO_4_, and filtered and concentrated in vacuo. The residue was purified using flash chromatography (CH_2_Cl_2_/CH_3_OH = 40:1, v/v) to afford white solid compound **25** (47 mg, 89%), R*_f_* = 0.40 (CH_2_Cl_2_/CH_3_OH = 20:1, *v*/*v*). ^1^H NMR (400 MHz, CDCl_3_): *δ* 8.00 (d, *J* = 7.6 Hz, 2H, Ar-*H*), 7.57 (t, *J* = 7.3 Hz, 1H, Ar-*H*), 7.45 (t, *J* = 7.6 Hz, 2H, Ar-*H*), 7.39–7.12 (m, 18H, Ar-*H*), 5.38 (t, *J* = 8.9 Hz, 1H, *H-3*), 5.22 (d, *J* = 8.3 Hz, 1H, *H-2*), 5.19–5.10 (m, 2H, Cbz-*CH_2_*), 4.84 (d, *J* = 7.1 Hz, 1H, *Glu-H-1*), 4.72 (d, *J* = 8.5 Hz, 1H, *Gal-H-1′*), 4.67–4.58 (m, 2H, Ph-C*H_2_*), 4.56–4.42 (m, 3H, CCl_3_*CH_2_*, N-Ph*CH_2_*), 4.36 (d, *J* = 9.5 Hz, 1H, *H-3′*), 4.09 (d, *J* = 9.1 Hz, 2H, *H-4′*, *H-5*), 4.04 (t, *J* = 8.9 Hz, 1H, *H-4*), 4.00–3.87 (m, 2H, *H-6a′*, CCl_3_*CH_2_*), 3.86–3.79 (m, 2H, OC*H_2_-a*, *H-6b′*), 3.76 (s, 3H, COOC*H_3_*), 3.54 (t, *J* = 4.9 Hz, 1H, *H-5′*), 3.42–3.24 (m, 2H, *H-2′*, OC*H_2_-b*), 3.24–3.11 (m, 2H, NC*H_2_*), 2.54–2.46 (m, 2H, Lev-C*H_2_*), 2.42–2.34 (m, 2H, Lev-C*H_2_*), 2.00 (s, 3H, Lev-C*H_3_*), 1.55–1.40 (m, 4H, OCH_2_C*H_2_*, C*H_2_*CH_2_N), 1.29–1.22 (m, 2H, OCH_2_CH_2_C*H_2_*). ^13^C NMR (100 MHz, CDCl_3_): *δ* 205.8, 171.8, 168.5, 165.1, 138.0, 137.4, 133.7, 130.1, 128.8, 128.7, 128.6, 128.2, 128.1, 128.0, 127.4, 127.3, 101.8, 100.9, 99.4, 95.7, 95.3, 78.6, 77.4, 74.9, 74.5, 73.9, 73.8, 71.9, 69.8, 69.0, 68.9, 68.9, 67.3, 62.8, 54.3, 53.0, 50.6, 47.3, 37.8, 29.7, 28.0, 23.2. HRMS (ESI) *m*/*z* calcd for C_55_H_67_Cl_2_^37^ClN_3_O_18_ [M+NH_4_]^+^1164.3450, found 1164.3483.

Compound **26**: To a solution of **25** (47 mg, 0.04 mmol) in dry pyridine (2 mL), DMAP (3 mg, 0.02 mmol) and TBDPSCl (32 μL, 0.12 mmol) were added under a nitrogen atmosphere. The reaction mixture was stirred at room temperature for 5 h, concentrated *in vacuo*, and extracted with DCM. The organic phase was washed with 1 N HCl, saturated NaHCO_3,_ and brine, dried with anhydrous Na_2_SO_4_, and filtered and concentrated in vacuo. The residue was purified using flash chromatography (PE/EtOAc = 4:1, *v*/*v*) to afford white solid compound **26** (50 mg, 88%), R*_f_* = 0.56 (PE/EtOAc = 1:1, *v*/*v*). ^1^H NMR (400 MHz, CDCl_3_): *δ* 8.01 (d, *J* = 7.5 Hz, 2H, Ar-*H*), 7.66 (t, *J* = 6.9 Hz, 4H, Ar-*H*), 7.57 (t, *J* = 7.4 Hz, 1H, Ar-*H*), 7.50–7.11 (m, 26H, Ar-*H*), 5.39 (t, *J* = 8.8 Hz, 1H, *H-3*), 5.24 (dd, *J* = 9.1, 7.7 Hz, 1H, *H-2*), 5.13 (d, *J* = 10.0 Hz, 2H, Cbz-*CH_2_*), 4.87 (d, *J* = 7.6 Hz, 1H, *Glu-H-1*), 4.69 (d, *J* = 8.1 Hz, 1H, *Gal-H-1′*), 4.67–4.57 (m, 2H, Ph-C*H_2_*), 4.53 (d, *J* = 11.5 Hz, 1H, CCl_3_*CH_2_*), 4.43 (s, 2H, N-Ph*CH_2_*), 4.41–4.33 (m, 1H, *H-3′*), 4.19 (s, 1H, *H-4′*), 4.12–4.00 (m, 2H, *H-4*, *H-5*), 3.95 (dd, *J* = 9.9, 7.2 Hz, 1H, *H-6a′*), 3.90–3.80 (m, 1H, CCl_3_*CH_2_*), 3.83–3.77 (m, 1H, *H-6b′*), 3.76–3.68 (m, 1H, OC*H_2_-a*), 3.65 (s, 3H, COOC*H_3_*), 3.57 (t, *J* = 6.4 Hz, 1H, *H-5′*), 3.33–3.07 (m, 4H, *H-2′*, OC*H_2_-b*, NC*H_2_*), 2.53–2.45 (m, 2H, Lev-C*H_2_*), 2.42–2.33 (m, 2H, Lev-C*H_2_*), 2.00 (s, 3H, Lev-C*H_3_*), 1.50–1.35 (m, 4H, OCH_2_C*H_2_*, C*H_2_*CH_2_N), 1.22–1.10 (m, 2H, OCH_2_CH_2_C*H_2_*), 1.03 (s, 9H, SiC(C*H_3_*)*_3_*). ^13^C NMR (100 MHz, CDCl_3_): *δ* 206.1, 205.8, 171.8, 168.4, 165.1, 138.0, 137.4, 135.7, 135.7, 133.6, 133.5, 133.4, 133.4, 130.1, 129.9, 129.3, 128.8, 128.7, 128.6, 128.2, 128.2, 128.0, 128.0, 127.9, 127.4, 127.3, 101.7, 99.3, 95.8, 78.9, 77.4, 74.9, 74.4, 74.2, 74.0, 73.7, 72.4, 71.9, 69.5, 67.4, 67.3, 62.4, 54.5, 52.9, 50.6, 37.8, 35.6, 29.7, 28.0, 26.9, 19.3. HRMS (ESI) *m/z* calcd for C_71_H_85_Cl_2_^37^ClN_3_O_18_Si [M+NH_4_]^+^1402.4628, found 1402.4628.

Compound **27**: To a solution of **26** (66 mg, 0.05 mmol) in dry DCM (2 mL), DMAP (12 mg, 0.10 mmol) and AllocCl (20 μL, 0.19 mmol) were added under a nitrogen atmosphere. The reaction mixture was stirred at room temperature for 4 h, then concentrated in vacuo. The residue was purified using flash chromatography (PE/EtOAc = 1.5:1, *v*/*v*) to afford white solid compound **27** (60 mg, 86%), R*_f_* = 0.51 (PE/EtOAc = 1:1, *v*/*v*). ^1^H NMR (400 MHz, CDCl_3_): *δ* 7.99 (d, *J* = 7.5 Hz, 2H, Ar-*H*), 7.64 (t, *J* = 5.8 Hz, 4H, Ar-*H*), 7.55 (t, *J* = 7.4 Hz, 1H, Ar-*H*), 7.48–7.17 (m, 22H, Ar-*H*), 7.17–7.11 (m, 1H, Ar-*H*), 5.91 (ddt, *J* = 16.3, 11.0, 5.7 Hz, 1H, C*H*=CH_2_), 5.41–5.29 (m, 3H, CH=C*H*_2*trans*_, *H-3*, *H-4′*), 5.23 (d, *J* = 10.4 Hz, 1H, CH=C*H*_2*cis*_), 5.20–5.09 (m, 3H, Cbz-*CH_2,_ H-2*), 4.85 (d, *J* = 7.4 Hz, 1H, *Glu-H-1*), 4.74 (d, *J* = 6.4 Hz, 1H, *Gal-H-1′*), 4.66–4.53 (m, 6H, Ph-C*H_2_*, CCl_3_*CH_2_*, *H-3′*, C*H_2_*-CH=CH_2_), 4.44 (s, 2H, N-Ph*CH_2_*), 4.19 (d, *J* = 15.5 Hz, 1H, CCl_3_*CH_2_*), 4.12 (t, *J* = 9.3 Hz, 1H, *H-4*), 4.03 (d, *J* = 9.8 Hz, 1H, *H-5*), 3.80–3.61 (m, 7H, COOC*H_3_*, *H-6′*, OC*H_2_-a*, *H-5′*), 3.36–3.06 (m, 4H, *H-2′*, OC*H_2_-b*, NC*H_2_*), 2.58–2.45 (m, 2H, Lev-C*H_2_*), 2.41–2.29 (m, 2H, Lev-C*H_2_*), 2.01 (s, 3H, Lev-C*H_3_*), 1.53–1.35 (m, 4H, OCH_2_C*H_2_*, C*H_2_*CH_2_N), 1.25–1.12 (m, 2H, OCH_2_CH_2_C*H_2_*), 1.03 (s, 9H, SiC(C*H_3_*)*_3_*). ^13^C NMR (100 MHz, CDCl_3_): *δ* 205.9, 171.8, 168.3, 165.0, 154.2, 138.0, 137.8, 137.0, 135.7, 135.7,133.5, 133.3, 132.0, 130.1, 129.8, 129.5, 128.7, 128.6, 128.5, 128.1, 128.1, 128.0, 128.0, 127.8, 127.4, 127.3, 118.7, 101.2, 99.3, 95.7, 77.4, 74.9, 74.7, 74.6, 74.4, 74.4, 74.0, 73.9, 73.0, 72.3, 69.8, 69.7, 68.7, 67.3, 62.1, 55.2, 52.8, 50.6, 50.4, 47.3, 46.2, 37.9, 29.8, 29.7, 28.0, 26.9, 23.3, 22.8, 19.3. HRMS (ESI) *m/z* calcd for C_75_H_89_Cl_3_N_3_O_20_Si [M+NH_4_]^+^ 1484.4869, found 1484.4868.

Compound **29**: To a solution of **27** (277 mg, 0.19 mmol) in THF/Py (1 mL/0.2 mL), HF·Py (200 μL) was added at 0 °C under a nitrogen atmosphere. After being warmed to room temperature, the mixture was stirred for 2 h. The resulting mixture was concentrated in vacuum and extracted with DCM. The organic phase was washed with 1 N HCl, saturated NaHCO_3,_ and brine, then dried with anhydrous Na_2_SO_4_, and filtered and concentrated in vacuo to afford crude product **28**, which could be used in the next reaction without purification.

To a solution of disaccharide acceptor **28** (232 mg, 0.19 mmol) and fucose donor **18** [26,27] (96 mg, 0.28 mmol) in dry DCM/Et_2_O (1 mL/1 mL), dried 4 Å molecular sieves were added under a nitrogen atmosphere at room temperature. The mixture was stirred at room temperature for 1 h and then cooled to −15 °C. NIS (64 mg, 0.28 mmol) and TfOH (5.7 µL, 0.06 mmol) were added to the reaction solution and stirred for 30 min. The reaction was quenched with Et_3_N and gradually warmed to room temperature. The mixture was filtered through celite and extracted with DCM. The organic phase was washed with saturated NaHCO_3_ and brine, dried with anhydrous Na_2_SO_4_, and filtered and concentrated in vacuo. The residue was purified using flash chromatography (PE/EtOAc = 1.5:1, *v*/*v*) to afford white solid compounds **29**alpha (164 mg, 58% for two steps) and **29**beta (91 mg, 32% for two steps), R*_f_* = 0.33 (PE/EtOAc = 1.5:1, *v*/*v*). Data for alpha anomer: ^1^H NMR (400 MHz, CDCl_3_): *δ* 7.98 (d, *J* = 7.4 Hz, 2H, Ar-*H*), 7.55 (t, *J* = 7.3 Hz, 1H, Ar-*H*), 7.43 (t, *J* = 7.5 Hz, 2H, Ar-*H*), 7.38–7.18 (m, 22H, Ar-*H*), 7.14 (d, *J* = 6.5 Hz, 1H, Ar-*H*), 5.95 (ddt, *J* = 16.1, 11.1, 5.7 Hz, 1H, C*H*=CH_2_), 5.41–5.36 (m, 1H, CH=C*H*_2*trans*_), 5.34 (d, *J* = 10.4 Hz, 1H, *H-3*), 5.28 (d, *J* = 10.9 Hz, 2H, *H-4′*, CH=C*H*_2*cis*_), 5.18–5.09 (m, 3H, *H-2*, Cbz-*CH_2_*), 4.83–4.66 (m, 5H, *Fuc-H-1″*, *Gal-H-1′*, *Glu-H-1*, Ph-C*H_2_*), 4.64 (d, *J* = 5.5 Hz, 2H, C*H_2_*-CH=CH_2_), 4.64–4.57 (m, 2H, Ph-C*H_2_*), 4.57 (d, *J* = 7.0 Hz, 1H, CCl_3_*CH_2_*), 4.50 (d, *J* = 9.0 Hz, 1H, *H-3′*), 4.45 (s, 2H, N-Ph*CH_2_*), 4.36–4.28 (m, 1H, *H-3″*), 4.17–3.98 (m, 5H, *H-4″*, *H-5″*, *H-4*, *H-5*, CCl_3_*CH_2_*), 3.81 (t, *J* = 6.1 Hz, 1H, *H-5′*), 3.77 (s, 3H, COO*Me*), 3.77–3.64 (m, 2H, *H-6a′*, OC*H_2_-a*), 3.49 (dd, *J* = 7.9, 3.3 Hz, 1H, *H-2″*), 3.45 (d, *J* = 6.7 Hz, 1H, *H-6b′*), 3.34–3.08 (m, 4H, *H-2′*, OC*H_2_-b*, NC*H_2_*), 2.51–2.44 (m, 2H, Lev-C*H_2_*), 2.39–2.31 (m, 2H, Lev-C*H_2_*), 2.00 (s, 3H, Lev-C*H_3_*), 1.52–1.41 (m, 4H, OCH_2_C*H_2_*, C*H_2_*CH_2_N), 1.40 (s, 3H, C*H_3_*), 1.34 (s, 3H, C*H_3_*), 1.34–1.29 (m, 2H, OCH_2_CH_2_C*H_2_*), 1.26 (d, *J* = 6.5 Hz, 3H, *H-6″*). ^13^C NMR (100 MHz, CDCl_3_): *δ* 205.9, 171.8, 168.3, 165.0, 154.4, 153.9, 138.5, 138.0, 137.7, 133.5, 131.9(CH=CH_2_), 130.1, 129.5, 128.7, 128.6, 128.5, 128.1, 128.0, 128.0, 127.9, 127.9, 127.4, 127.3, 118.9(CH=CH_2_), 108.9, 101.1(C-1), 99.6(C-1′), 98.0(C-1″), 95.8, 77.4(C-4), 76.5(C-2″), 76.3(C-4″), 75.8(C-3″), 74.9, 74.6(C-3′), 74.5(C-5), 74.3(C-3), 74.2, 73.9, 73.3(C-4′), 72.4, 72.1(C-5′, C-2), 69.8(O-CH_2_), 68.9(CH_2_-CH=CH_2_), 67.3(Cbz-CH_2_), 66.3(C-6′), 63.5(C-5″), 55.0(C-2′), 52.8(COOMe), 50.4(N-PhCH_2_), 47.4(N-CH_2_), 46.2(N-CH_2_), 37.8(Lev-CH_2_), 32.1, 31.6, 29.8, 29.7(Lev-CH_3_), 29.5, 28.3(CH_3_), 28.0(Lev-CH_2_), 26.5(CH_3_), 23.3, 22.8, 16.4(C-6″), 14.3. HRMS (ESI) *m*/*z* calcd for C_75_H_91_Cl_2_^37^ClN_3_O_24_ [M+NH_4_]^+^1524.5023, found: 1524.5039; Data for beta anomer: ^1^H NMR (400 MHz, CDCl_3_): *δ* 7.99 (d, *J* = 7.0 Hz, 2H, Ar-*H*), 7.56 (t, *J* = 7.0 Hz, 1H, Ar-*H*), 7.49–7.17 (m, 22H, Ar-*H*), 7.14 (d, *J* = 5.7 Hz, 1H, Ar-*H*), 5.93 (ddt, *J* = 16.2, 10.8, 5.6 Hz, 1H, C*H*=CH_2_), 5.33 (dd, *J* = 17.7, 7.0 Hz, 3H, *H-3*, *H-4′*, CH=C*H*_2*trans*_), 5.25 (d, *J* = 10.5 Hz, 1H, CH=C*H*_2*cis*_), 5.20–5.09 (m, 3H, *H-2*, Cbz-*CH_2_*), 4.78 (q, *J* = 11.9 Hz, 4H, *Gal-H-1′*, *Glu-H-1*, Ph-C*H_2_*), 4.67–4.48 (m, 6H, C*H_2_*-CH=CH_2_, Ph-C*H_2_*, CCl_3_*CH_2_*, *H-3′*), 4.44 (s, 2H, N-Ph*CH_2_*), 4.29 (d, *J* = 8.0 Hz, 1H, *Fuc-H-1″*), 4.11 (dt, *J* = 12.0, 6.6 Hz, 2H, *H-3″*, *H-4*), 4.07–3.95 (m, 3H, *H-4″*, *H-5*, CCl_3_*CH_2_*), 3.86 (dd, *J* = 9.7, 4.9 Hz, 1H, *H-6a′*), 3.83–3.70 (m, 4H, *H-5″*, *H-5′*, *H-6b′*, OC*H_2_-a*), 3.70 (s, 3H, COO*Me*), 3.33 (t, *J* = 7.5 Hz, 1H, *H-2″*), 3.30–3.07 (m, 4H, *H-2′*, OC*H_2_-b*, NC*H_2_*), 2.556–2.41 (m, 2H, Lev-C*H_2_*), 2.41–2.25 (m, 2H, Lev-C*H_2_*), 2.00 (s, 3H, Lev-C*H_3_*), 1.36 (d, *J* = 6.4 Hz, 3H, *H-6″*), 1.31 (s, 3H, C*H_3_*), 1.30 (s, 3H, C*H_3_*), 1.28–1.25 (m, 6H, OCH_2_C*H_2_*, C*H_2_*CH_2_N, OCH_2_CH_2_C*H_2_*). ^13^C NMR (151 MHz, CDCl_3_): *δ* 205.9, 177.5, 171.8, 165.0, 154.3, 138.6, 138.0, 137.6, 133.5, 131.9(CH=CH_2_), 130.1, 128.6, 128.5, 128.4, 128.3, 128.1, 128.0, 128.0, 127.6, 127.4, 127.4, 127.3, 118.7(CH=CH_2_), 109.7, 109.7, 103.2(C-1″), 101.2(C-1), 99.2(C-1′), 79.0(C-3″, C-2″), 76.4(C-4″), 74.8, 74.4(C-5), 74.2(C-3), 73.7, 73.5, 73.3(C-4′), 72.3(C-5′), 72.0(C-2), 69.8(O-CH_2_), 68.9(CH_2_-CH=CH_2_), 68.8(C-5″), 67.7(C-6′), 67.3(Cbz-CH_2_), 60.6, 54.9(C-2′), 52.8(COOMe), 50.6(N-PhCH_2_), 50.4, 47.3(N-CH_2_), 46.2(N-CH_2_), 37.8(Lev-CH_2_), 32.0, 31.6, 29.7(Lev-CH_3_), 28.0(Lev-CH_2_), 27.9(CH_3_), 26.5(CH_3_), 23.2, 22.8, 21.2, 16.7(C-6″), 14.3, 14.3. TOF-HRMS (ESI) *m*/*z*: calcd for C_75_H_91_Cl_2_^37^ClN_3_O_24_ [M+NH_4_]^+^1524.5023, found: 1524.5039.

Compound **30**: Following the synthesis of compound **29**, compounds **28** (23 mg, 0.02 mmol) and **20** (16 mg, 0.03 mmol) were reacted under the catalytic reaction of NIS (6 mg, 0.03 mmol) and TfOH (0.6 μL, 0.01 mmol) to obtain the corresponding product, which was purified using flash chromatography (PE/EtOAc = 15:1, *v*/*v*) to afford white solid compounds **30**alpha (16 mg, 67% for two steps) and **30**beta (6 mg, 25% for two steps), R*_f_* = 0.51 (PE/EtOAc = 1:1, *v*/*v*). Data for alpha anomer: ^1^H NMR (400 MHz, CDCl_3_): *δ* 7.97 (d, *J* = 7.5 Hz, 2H, Ar-*H*), 7.83–7.67 (m, 8H, Ar-*H*), 7.54 (t, *J* = 7.5 Hz, 1H, Ar-*H*), 7.50–7.17 (m, 29H, Ar-*H*), 7.13 (d, *J* = 6.9 Hz, 1H, Ar-*H*), 5.89 (ddt, *J* = 16.2, 11.3, 5.7 Hz, 1H, C*H*=CH_2_), 5.36–5.27 (m, 3H, CH=C*H*_2*trans*_, *H-3*, *H-4′*), 5.20 (d, *J* = 10.3 Hz, 1H, CH=C*H*_2*cis*_), 5.18–5.07 (m, 4H, Ph-C*H_2_*, *H-2*, Cbz-*CH_2_*), 4.94 (dd, *J* = 11.8, 1.9 Hz, 2H, Ph-C*H_2_*), 4.88 (d, *J* = 3.4 Hz, 1H, *Fuc-H-1″*), 4.86–4.78 (m, 4H, *Glu-H-1*, Ph-C*H_2_*), 4.65 (d, *J* = 7.9 Hz, 1H, *Gal-H-1′*), 4.58 (dd, *J* = 10.2, 5.8 Hz, 5H, C*H_2_*-CH=CH_2_, Ph-C*H_2,_* CCl_3_*CH_2_*), 4.49 (d, *J* = 12.7 Hz, 1H, *H-3′*), 4.43 (s, 2H, N-Ph*CH_2_*), 4.24–4.16 (m, 1H, CCl_3_*CH_2_*), 4.13 (dd, *J* = 10.2, 3.5 Hz, 1H, *H-2″*), 4.08–4.01 (m, 2H, *H-3″*, *H-4*), 3.99 (d, *J* = 9.6 Hz, 1H, *H-5*), 3.87–3.80 (m, 2H, *H-5″*, *H-5′*), 3.73 (dd, *J* = 9.8, 4.5 Hz, 2H, *H-6a′*, *H-4″*), 3.66 (s, 4H, OC*H_2_-a*, COO*Me*), 3.48 (dd, *J* = 9.8, 6.0 Hz, 1H, *H-6b′*), 3.33–3.03 (m, 4H, *H-2′*, OC*H_2_-b*, NC*H_2_*), 2.57–2.41 (m, 2H, Lev-C*H_2_*), 2.41–2.27 (m, 2H, Lev-C*H_2_*), 2.00 (s, 3H, Lev-C*H_3_*), 1.44–1.29 (m, 4H, OCH_2_C*H_2_*, C*H_2_*CH_2_N), 1.20–1.09 (m, 2H, OCH_2_CH_2_C*H_2_*), 1.06 (d, *J* = 6.5 Hz, 3H, *H-6″*). ^13^C NMR (100 MHz, CDCl_3_): *δ* 205.8, 171.8, 168.2, 165.0, 154.5, 139.2, 138.0, 137.7, 136.3, 136.2, 133.5, 133.4, 133.3, 133.1, 131.8(CH=CH_2_), 130.1, 129.5, 128.7, 128.6, 128.6, 128.5, 128.3, 128.1, 128.1, 128.1, 128.0, 127.8, 127.7, 127.4, 127.3, 127.2, 126.8, 126.7, 126.2, 126.1, 126.0, 125.9, 118.8(CH=CH_2_), 101.1(C-1), 99.3(C-1′), 98.4(C-1″), 95.8, 83.3, 79.6(C-4), 77.7(C-4″), 77.4(C-3″), 76.6(C-2″), 75.0, 74.9, 74.5(C-5), 74.4(C-3′), 74.3(C-3), 74.0, 73.6, 73.4, 73.4(C-4′), 72.4(C-5′), 72.2(C-2), 69.6(O-CH_2_), 68.8(CH_2_-CH=CH_2_), 67.3(Cbz-CH_2_), 66.6(C-5″), 66.2(C-6′), 65.7, 55.0(C-2′), 52.7(COOMe), 50.6(N-PhCH_2_), 50.4, 47.3(N-CH_2_), 46.3(N-CH_2_), 37.8(Lev-CH_2_), 29.7(Lev-CH_3_), 28.0(Lev-CH_2_), 23.3, 16.9(C-6″). HRMS (ESI) *m*/*z* calcd for C_94_H_103_Cl_3_N_3_O_24_ [M+NH_4_]^+^ 1762.5992, found 1762.6027; Data for beta anomer: ^1^H NMR (400 MHz, CDCl_3_): *δ* 7.99 (d, *J* = 7.7 Hz, 2H, Ar-*H*), 7.86–7.67 (m, 8H, Ar-*H*), 7.58–7.39 (m, 9H, Ar-*H*), 7.37–7.18 (m, 22H, Ar-*H*), 7.13 (d, *J* = 6.4 Hz, 1H, Ar-*H*), 5.87 (ddt, *J* = 16.4, 10.9, 5.7 Hz, 1H, C*H*=CH_2_), 5.42–5.27 (m, 3H, CH=C*H*_2*trans*_, *H-3*, *H-4′*), 5.23 (d, *J* = 10.3 Hz, 1H, CH=C*H*_2*cis*_), 5.19–5.07 (m, 5H, Ph-C*H_2_*, *H-2*, Cbz-*CH_2_*), 4.91 (d, *J* = 12.7 Hz, 2H, Ph-C*H_2_*), 4.88–4.79 (m, 1H, Ph-C*H_2_*), 4.74 (d, *J* = 11.8 Hz, 2H, Ph-C*H_2_*, *Glu-H-1*), 4.66 (d, *J* = 8.1 Hz, 1H, *Gal-H-1′*), 4.62–4.50 (m, 5H, C*H_2_*-CH=CH_2_, Ph-C*H_2,_* CCl_3_*CH_2_*), 4.43 (d, *J* = 7.2 Hz, 3H, *H-3′*, N-Ph*CH_2_*), 4.37 (d, *J* = 7.6 Hz, 1H, *Fuc-H-1″*), 4.02 (d, *J* = 9.0 Hz, 2H, CCl_3_*CH_2_*, *H-4*), 3.97 (d, *J* = 9.7 Hz, 1H, *H-5*), 3.89 (t, *J* = 8.7 Hz, 2H, *H-6a′*, *H-2″*), 3.83–3.63 (m, 3H, OC*H_2_-a*, *H-6b′*, *H-5′*), 3.60 (s, 4H, *H-4″*, COO*Me*), 3.57–3.49 (m, 1H, *H-3″*), 3.45 (q, *J* = 6.4 Hz, 1H, *H-5″*), 3.32–3.02 (m, 4H, *H-2′*, OC*H_2_-b*, NC*H_2_*), 2.56–2.41 (m, 2H, Lev-C*H_2_*), 2.41–2.26 (m, 2H, Lev-C*H_2_*), 2.00 (s, 3H, Lev-C*H_3_*), 1.42–1.30 (m, 6H, OCH_2_C*H_2_*, C*H_2_*CH_2_N, OCH_2_CH_2_C*H_2_*), 1.18 (d, *J* = 6.3 Hz, 3H, *H-6″*). ^13^C NMR (151 MHz, CDCl_3_): *δ* 205.9, 171.8, 164.9, 138.7, 138.0, 137.7, 136.7, 136.0, 133.5, 133.2, 133.1, 133.0, 131.8(CH=CH_2_), 130.1, 128.6, 128.5, 128.5, 128.2, 128.1, 128.0, 128.0, 127.8, 127.7, 127.7, 127.4, 126.9, 126.8, 126.6, 126.1, 126.0, 125.9, 125.8, 118.7(CH=CH_2_), 104.1(C-1″), 101.1(C-1), 99.5(C-1′), 82.6(C-3″), 79.3(C-2″), 76.1(C-4″), 75.1, 74.8, 74.7(C-3′), 74.4(C-5), 74.3(C-3), 73.8, 73.5, 73.3(C-4′), 72.3(C-5′), 72.1(C-2), 70.6(C-5″), 69.7(O-CH_2_), 68.8(CH_2_-CH=CH_2_), 67.7(C-6′), 67.3(Cbz-CH_2_), 55.0(C-2′), 52.7(COOMe), 50.6(N-PhCH_2_), 50.4, 47.3(N-CH_2_), 46.2(N-CH_2_), 37.8(Lev-CH_2_), 30.3, 29.8, 29.7(Lev-CH_3_), 29.5, 29.2, 28.0(Lev-CH_2_), 23.2, 22.8, 17.1(C-6″), 14.3. TOF-HRMS (ESI) *m*/*z*: calcd for C_94_H_103_Cl_3_N_3_O_24_ [M+NH_4_]^+^ 1762.5992, found 1762.6027.

Compound **33**: To a solution of **29** (33 mg, 0.02 mmol) in DCM/CH_3_OH (1 mL/1 mL), Hydrazine acetate (92 mg, 0.22 mmol) was added at room temperature under a nitrogen atmosphere. The reaction was quenched with acetone after 2 h and then extracted with DCM. The organic phase was washed with saturated NaHCO_3_ and brine, dried with anhydrous Na_2_SO_4_, and filtered and concentrated in vacuo to afford crude product **31**. This compound was suitable for the next step without purification.

Following the synthesis of compound **29**, compounds **31** (27 mg, 0.02 mmol) and **18** (10 mg, 0.03 mmol) were reacted under the catalytic reaction of NIS (6 mg, 0.03 mmol) and TfOH (1.2 μL, 0.01 mmol) to obtain the corresponding product after 15 min, which was purified using flash chromatography (PE/EtOAc = 2:1, *v*/*v*) to afford white solid compound **33** (17 mg, 72% for two steps, only α), R*_f_* = 0.29 (PE/EtOAc = 2:1, *v*/*v*). ^1^H NMR (400 MHz, CDCl_3_): *δ* 8.05 (d, *J* = 7.3 Hz, 3H, Ar-*H*), 7.57 (t, *J* = 7.4 Hz, 1H, Ar-*H*), 7.43 (dd, *J* = 17.3, 9.5 Hz, 3H, Ar-*H*), 7.38–7.20 (m, 34H, Ar-*H*), 7.16–7.10 (m, 5H, Ar-*H*), 7.09–7.01 (m, 2H, Ar-*H*), 5.98 (ddt, *J* = 16.6, 11.3, 5.9 Hz, 1H, C*H*=CH_2_), 5.43–5.33 (m, 2H, CH=C*H*_2*trans*_, *H-2*), 5.33–5.25 (m, 2H, CH=C*H*_2*cis*_, *H-4′*), 5.19–5.09 (m, 3H, Cbz-*CH_2_*, *Fuc-H-1‴*), 4.83–4.71 (m, 5H, *Gal-H-1′*, *Fuc-H-1″*, Ph-C*H_2_*, *Glu-H-1*), 4.69 (d, *J* = 10.9 Hz, 4H, C*H_2_*-CH=CH_2_, Ph-C*H_2_*), 4.56 (dd, *J* = 12.4, 6.2 Hz, 2H, Troc-C*H_2_*, Ph-C*H_2_*), 4.50 (d, *J* = 7.4 Hz, 1H, *H-3′*), 4.46 (s, 2H, N-Ph*CH_2_*), 4.35–4.28 (m, 3H, Troc-C*H_2_*, Ph-C*H_2_*, *H-3″*), 4.26 (dd, *J* = 6.4, 1.7 Hz, 1H, *H-5‴*), 4.17–4.13 (m, 2H, *H-3‴*), 4.08–4.01 (m, 5H, *H-4″*, *H-5″*, *H-4*, *H-5*, *H-3*), 3.82 (t, *J* = 5.5 Hz, 1H, *H-5′*), 3.76 (s, 5H, COO*Me*, *H-6a′*, OC*H_2_-a*), 3.49 (dd, *J* = 7.8, 3.4 Hz, 3H, *H-6b′*, *H-2″*, *H-4‴*), 3.27 (d, *J* = 3.7 Hz, 2H, *H-2‴*, OC*H_2_-b*), 3.23–3.09 (m, 3H, *H-2′*, NC*H_2_*), 1.51–1.41 (m, 4H, OCH_2_C*H_2_*, C*H_2_*CH_2_N), 1.39 (s, 3H, C*H_3_*), 1.34 (s, 3H, C*H_3_*), 1.26 (d, *J* = 5.4 Hz, 5H, OCH_2_CH_2_C*H_2_*, *H-6″*), 1.20 (s, 3H, C*H_3_*), 1.15 (s, 3H, C*H_3_*), 1.04 (d, *J* = 6.5 Hz, 3H, *H-6‴*). ^13^C NMR (125 MHz, CDCl_3_): *δ* 138.5, 138.0, 137.9, 133.5, 131.9(CH=CH_2_), 130.0, 128.9, 128.7, 128.6, 128.6, 128.5, 128.4, 128.3, 128.0, 127.9, 127.8, 127.5, 127.4, 127.3, 118.9(CH=CH_2_), 108.9, 108.7, 101.2(C-1), 99.5(C-1′), 97.9(C-1″), 96.2(C-1*‴*), 78.1(C-4), 76.4(C-2″), 76.3(C-4″), 75.9(C-3″), 75.8(C-4*‴*), 75.7(C-3), 75.3(C-3*‴*), 75.1(C-3′), 75.0, 74.8(C-2*‴*), 74.7(C-5), 74.4(C-2), 73.9, 73.9, 73.2(C-4′), 72.4, 72.0(C-5′), 72.0, 71.7, 70.1, 69.8(C-6′), 68.9(CH_2_-CH=CH_2_), 67.3(Cbz-CH_2_), 66.2(C-6′), 63.6(C-5*‴*), 63.4(C-5″), 55.1(C-2′), 52.8(COOMe), 50.4(N-PhCH_2_), 47.3(N-CH_2_), 46.2(N-CH_2_), 32.1, 31.6, 28.3(CH_3_), 27.8(CH_3_), 26.5(CH_3_), 26.2(CH_3_), 22.8, 22.1, 16.4(C-6″), 16.1(C-6*‴*), 14.3. HRMS (ESI) *m*/*z*: calcd for C_86_H_101_N_2_O_26_Cl_3_Na [M+Na]^+^1705.5600, found: 1705.5641.

Compound **34**: To a solution of **30** (93 mg, 0.05 mmol) in DCM/CH_3_OH (1 mL/1 mL), Hydrazine acetate (50 mg, 0.54 mmol) was added at room temperature under a nitrogen atmosphere. The reaction was quenched with acetone after 2 h and then extracted with DCM. The organic phase was washed with saturated NaHCO_3_ and brine, dried with anhydrous Na_2_SO_4_, and filtered and concentrated in vacuo to afford crude product **32**. This compound was suitable for the next step without purification.

Following the synthesis of compound **29**, compounds **32** (87 mg, 0.05 mmol) and **20** (50 mg, 0.08 mmol) were reacted under the catalytic reaction of NIS (18 mg, 0.08 mmol) and TfOH (3.2 μL, 0.03 mmol) to obtain the corresponding product after 15 min, which was purified using flash chromatography (PE/EtOAc = 2:1, *v*/*v*) to afford white solid compound **34** (95 mg, 83% for two steps, only α), R*_f_* = 0.24 (PE/EtOAc = 2:1, *v*/*v*). ^1^H NMR (400 MHz, CDCl_3_): *δ* 8.01 (d, *J* = 7.4 Hz, 2H, Ar-*H*), 7.82–7.64 (m, 11H, Ar-*H*), 7.64–7.56 (m, 4H, Ar-*H*), 7.53 (d, *J* = 7.4 Hz, 2H, Ar-*H*), 7.52–7.25 (m, 29H, Ar-*H*), 7.12 (t, *J* = 7.3 Hz, 3H, Ar-*H*), 7.04 (dd, *J* = 13.6, 7.1 Hz, 3H, Ar-*H*), 5.92 (ddt, *J* = 16.3, 11.0, 5.8 Hz, 1H, C*H*=CH_2_), 5.43 (t, *J* = 7.6 Hz, 1H, *H-2*), 5.31 (d, *J* = 9.2 Hz, 1H, CH=C*H*_2*trans*_), 5.28 (dd, *J* = 7.7, 4.4 Hz, 2H, *Fuc-H-1‴*, *H-4′*), 5.19 (d, *J* = 10.4 Hz, 2H, CH=C*H*_2*cis*_), 5.14 (d, *J* = 8.3 Hz, 2H, Cbz-*CH_2_*), 5.09 (d, *J* = 11.8 Hz, 1H, Ph-C*H_2_*), 4.94 (dd, *J* = 11.9, 3.2 Hz, 3H, Ph-C*H_2_*), 4.87 (d, *J* = 3.5 Hz, 1H, *Fuc-H-1″*), 4.81 (q, *J* = 12.3 Hz, 4H, Ph-C*H_2_*), 4.76–4.69 (m, 2H, *Gal-H-1′*, *Glu-H-1*), 4.68 (d, *J* = 11.0 Hz, 3H, Ph-C*H_2_*, C*H_2_*-CH=CH_2_), 4.65–4.57 (m, 4H, Ph-C*H_2_*, Troc-C*H_2_*, C*H_2_*-CH=CH_2_), 4.57–4.50 (m, 3H, Ph-C*H_2_*, Troc-C*H_2_*, *H-3′*), 4.46–4.35 (m, 3H, N-Ph*CH_2_*, Ph-C*H_2_*), 4.15–4.10 (m, 1H, *H-2″*), 4.09–4.01 (m, 3H, *H-4*, *H-3″*, *H-3*), 3.97 (d, *J* = 9.0 Hz, 1H, *H-5*), 3.91 (dq, *J* = 10.5, 5.0, 3.5 Hz, 2H, *H-5‴*, *H-2‴*), 3.87–3.76 (m, 3H, *H-3‴*, *H-5″*, *H-5′*), 3.73 (dd, *J* = 8.9, 4.7 Hz, 2H, *H-6a′*, *H-4″*), 3.66 (s, 4H, COO*Me*, OC*H_2_-a*), 3.47 (dd, *J* = 9.7, 5.2 Hz, 1H, *H-6b′*), 3.22 (s, 1H, *H-4‴*), 3.21–3.10 (m, 3H, NC*H_2_-a*, OC*H_2_-b*, *H-2′*), 3.10–3.03 (m, 1H, NC*H_2_-b*), 1.25–1.19 (m, 4H, OCH_2_C*H_2_*, C*H_2_*CH_2_N), 1.18–1.11 (m, 2H, OCH_2_CH_2_C*H_2_*), 1.06 (d, *J* = 6.4 Hz, 3H, *H-6″*), 0.71 (d, *J* = 6.4 Hz, 3H, *H-6‴*). ^13^C NMR (150 MHz, CDCl_3_): *δ* 168.9, 164.7, 154.5, 139.2, 139.1, 138.0, 137.7, 136.3, 136.2, 135.9, 135.7, 133.5, 133.4, 133.3, 133.3, 133.2, 133.1, 133.1, 132.0(CH=CH_2_), 130.0, 128.9, 128.7, 128.6, 128.4, 128.3, 128.2, 128.1, 128.0, 127.8, 127.8, 127.7, 127.7, 127.6, 127.4, 127.3, 127.2, 126.9, 126.9, 126.8, 126.2, 126.1, 126.0, 126.0, 125.9, 125.8, 118.8(CH=CH_2_), 101.3(C-1), 99.4(C-1′), 98.3(C-1″), 96.8(C-1*‴*), 79.6(C-3″), 78.5(C-4), 78.3(C-3*‴*), 77.7(C-4″), 77.5(C-4*‴*), 76.5(C-2″), 75.7(C-2*‴*), 75.3(C-3), 75.2(Troc-CH_2_), 75.0, 75.0, 74.8(C-3), 74.6(C-2), 74.0, 73.8, 73.6(C-3′), 73.5, 73.3, 73.2(C-4′), 72.4, 72.2(C-5′), 69.7, 69.7(O-CH_2_), 69.6, 68.9(CH_2_-CH=CH_2_), 67.3(Cbz-CH_2_), 66.7(C-5*‴*), 66.6(C-5″), 66.0(C-6′), 66.0, 60.5, 55.2(C-2′), 52.7(COOMe), 50.6(N-PhCH_2_), 50.4, 47.3(N-CH_2_), 46.2(N-CH_2_), 44.4, 32.1, 31.6, 31.6, 30.3, 29.8, 29.8, 29.5, 29.2, 29.1, 23.2, 22.8, 21.2, 16.8(C-6″), 16.3(C-6*‴*), 14.3, 14.3. HRMS (ESI) *m*/*z* calcd for C_124_H_125_Cl_3_N_2_O_26_NH_4_ [M+ NH_4_]^+^ 2180.7930, found 2180.7883. 

Compound **35**: To a solution of **33** (23 mg, 0.01 mmol) in THF/Ac_2_O/AcOH (1.8 mL/0.3 mL/0.3 mL), zinc dust (72 mg, 1.09 mmol) was added at room temperature under a nitrogen atmosphere. After being stirred for 10 h, the mixture was filtered through celite and concentrated in vacuo. The residue was purified using flash chromatography (PE/EtOAc = 1:1, *v*/*v*) to afford white solid compound **35** (17 mg, 80%), R*_f_* = 0.23 (PE/EtOAc = 1:1, *v*/*v*). ^1^H NMR (400 MHz, CDCl_3_): *δ* 8.03 (d, *J* = 7.3 Hz, 2H, Ar-*H*), 7.58 (t, *J* = 7.3 Hz, 1H, Ar-*H*), 7.48–7.02 (m, 37H, Ar-*H*), 5.97 (ddt, *J* = 16.2, 11.2, 5.7 Hz, 1H, C*H*=CH_2_), 5.43–5.33 (m, 2H, CH=C*H*_2*trans*_, *H-2*), 5.31–5.27 (m, 2H, CH=C*H*_2*cis*_, *H-4′*), 5.18–5.10 (m, 3H, Cbz-*CH_2_*, *Fuc-H-1‴*), 4.97 (d, *J* = 8.3 Hz, 1H, *Gal-H-1′*), 4.82–4.73 (m, 4H, *Fuc-H-1″*, Ph-C*H_2_*, *H-3′*), 4.68 (t, *J* = 6.4 Hz, 6H, C*H_2_*-CH=CH_2_, Ph-C*H_2_*, *Glu-H-1*), 4.56 (d, *J* = 12.6 Hz, 1H, Ph-C*H_2_*), 4.45 (s, 2H, N-Ph*CH_2_*), 4.32 (dd, *J* = 7.7, 5.6 Hz, 2H, Ph-C*H_2_*, *H-3″*), 4.28 (dd, *J* = 6.6, 2.2 Hz, 1H, *H-5‴*), 4.15 (ddd, *J* = 8.9, 6.5, 2.3 Hz, 2H, *H-3‴*, *H-3*), 4.12–4.02 (m, 4H, *H-4″*, *H-5″*, *H-4*, *H-5*), 3.84 (t, *J* = 6.0 Hz, 1H, *H-5′*), 3.76 (s, 3H, COO*Me*), 3.76–3.70 (m, 2H, *H-6a′*, OC*H_2_-a*), 3.52 (dd, *J* = 5.2, 1.8 Hz, 1H, *H-4‴*), 3.49 (dd, *J* = 7.8, 3.4 Hz, 2H, *H-6b′*, *H-2″*), 3.34–3.25 (m, 2H, *H-2‴*, OC*H_2_-b*), 3.17–3.04 (m, 3H, *H-2′*, NC*H_2_*), 1.39 (s, 3H, C*H_3_*), 1.38–1.34 (m, 4H, OCH_2_C*H_2_*, C*H_2_*CH_2_N), 1.33 (s, 3H, C*H_3_*), 1.26 (d, *J* = 5.1 Hz, 5H, OCH_2_CH_2_C*H_2_*, *H-6″*), 1.20 (s, 3H, C*H_3_*), 1.16 (s, 3H, C*H_3_*), 1.03 (d, *J* = 6.6 Hz, 3H, *H-6‴*). ^13^C NMR (100 MHz, CDCl_3_): *δ* 168.8, 164.7, 154.4, 138.6, 138.1, 138.0, 133.6, 132.0, 130.0, 129.0, 128.7, 128.6, 128.5, 128.4, 128.3, 128.1, 128.0, 127.9, 127.9, 127.8, 127.5, 127.5, 127.4, 118.8, 108.8, 108.7, 101.4, 98.8, 98.0, 96.4, 78.2, 77.4, 76.5, 76.3, 76.2, 75.8, 75.7, 75.3, 75.0, 74.9, 74.5, 73.5, 72.4, 72.3, 72.2, 71.8, 69.9, 68.8, 67.3, 63.7, 63.4, 55.6, 52.8, 50.4, 47.1, 46.1, 32.1, 31.6, 30.3, 29.8, 29.5, 28.3, 27.8, 26.5, 26.2, 23.1, 22.8, 16.4, 16.1, 14.3, 10.4. HRMS (ESI) *m*/*z* calcd for C_85_H_106_N_3_O_25_ [M+NH_4_]^+^1568.7110, found: 1568.7158.

Compound **36**: Following the synthesis of compound **35**, compound **34** (86 mg, 0.04 mmol) was reacted under zinc dust (104 mg, 1.59 mmol) to give the corresponding product, which was purified using flash chromatography (PE/EtOAc = 1:1, *v*/*v*) to afford white solid compound **36** (74 mg, 91%), R*_f_* = 0.26 (PE/EtOAc = 1.5:1, *v*/*v*). ^1^H NMR (500 MHz, CDCl_3_): *δ* 7.88 (d, *J* = 7.3 Hz, 2H, Ar-*H*), 7.74–7.56 (m, 11H, Ar-*H*), 7.55–7.46 (m, 4H, Ar-*H*), 7.46–7.40 (m, 2H, Ar-*H*), 7.40–7.29 (m, 11H, Ar-*H*), 7.29–7.15 (m, 18H, Ar-*H*), 7.15–7.06 (m, 3H, Ar-*H*), 7.03 (t, *J* = 7.5 Hz, 3H, Ar-*H*), 6.95 (dd, *J* = 14.7, 7.2 Hz, 3H, Ar-*H*), 5.83 (ddt, *J* = 17.3, 10.4, 5.7 Hz, 1H, C*H*=CH_2_), 5.35 (t, *J* = 8.1 Hz, 1H, *H-2*), 5.23 (dq, *J* = 17.3, 1.6 Hz, 1H, CH=C*H*_2*trans*_), 5.20–5.17 (m, 2H, *Fuc-H-1‴*, *H-4′*), 5.12 (dq, *J* = 10.4, 1.3 Hz, 1H, CH=C*H*_2*cis*_), 5.06–4.97 (m, 3H, Cbz-*CH_2_*, Ph-C*H_2_*), 4.84 (td, *J* = 10.0, 8.8, 4.0 Hz, 4H, *Gal-H-1′*, Ph-C*H_2_*), 4.80 (d, *J* = 3.6 Hz, 1H, *Fuc-H-1″*), 4.78–4.70 (m, 3H, Ph-C*H_2_*), 4.65 (d, *J* = 11.9 Hz, 2H, Ph-C*H_2_*, *H-3′*), 4.58 (d, *J* = 11.6 Hz, 2H, Ph-C*H_2_*, *Glu-H-1*), 4.56–4.47 (m, 5H, C*H_2_*-CH=CH_2_, Ph-C*H_2_*), 4.43 (d, *J* = 10.7 Hz, 1H, Ph-C*H_2_*), 4.36–4.29 (m, 3H, N-Ph*CH_2_*, Ph-C*H_2_*), 4.07–4.01 (m, 2H, *H-3*, *H-2″*), 3.97–3.92 (m, 2H, *H-4*, *H-3″*), 3.90 (d, *J* = 4.3 Hz, 1H, *H-5*), 3.86–3.75 (m, 4H, *H-2‴*, *H-5″*, *H-5′*, *H-5‴*), 3.70 (dd, *J* = 10.2, 2.7 Hz, 1H, *H-3‴*), 3.67–3.63 (m, 1H, *H-6a′*), 3.62 (d, *J* = 2.7 Hz, 1H, *H-4″*), 3.57 (s, 3H, COO*Me*), 3.54–3.45 (m, 1H, OC*H_2_-a*), 3.44–3.37 (m, 1H, *H-6b′*), 3.17–3.12 (m, 1H, *H-4‴*), 3.12–3.03 (m, 2H, NC*H_2_-a*, OC*H_2_-b*), 3.02–2.94 (m, 2H, NC*H_2_-b*, *H-2′*), 1.20–1.13 (m, 4H, OCH_2_C*H_2_*, C*H_2_*CH_2_N), 1.06–0.99 (m, 1H, OCH_2_CH_2_C*H_2_*), 0.98 (d, *J* = 6.4 Hz, 3H, *H-6″*), 0.97–0.90 (m, 1H, OCH_2_CH_2_C*H_2_*), 0.60 (d, *J* = 6.4 Hz, 3H, *H-6‴*). ^13^C NMR (150 MHz, CDCl_3_): *δ* 168.8, 164.7, 154.5, 139.2, 139.1, 138.0, 137.7, 136.3, 136.2, 135.9, 135.7, 133.6, 133.4, 133.3, 133.3, 133.2, 133.1, 133.0, 131.9, 130.0, 129.8, 128.9, 128.7, 128.5, 128.4, 128.2, 128.1, 128.1, 128.0, 128.0, 128.0, 127.8, 127.8, 127.7, 127.7, 127.6, 127.6, 127.4, 127.3, 127.2, 126.9, 126.8, 126.7, 126.1, 126.1, 126.0, 126.0, 125.9, 125.9, 125.8, 118.8, 101.5, 98.8, 98.4, 97.0, 79.5, 78.5, 78.5, 77.8, 77.5, 76.5, 75.7, 75.4, 75.1, 75.0, 75.0, 74.8, 74.7, 74.5, 73.6, 73.5, 73.4, 73.2, 72.6, 72.4, 69.7, 69.4, 68.8, 67.3, 66.7, 66.5, 55.6, 52.6, 50.5, 50.3, 47.2, 46.1, 42.1, 32.1, 31.6, 30.3, 29.5, 23.1, 22.8, 16.9, 16.3, 14.3. HRMS (ESI) *m*/*z* calcd for C_123_H_127_N_2_O_25_ [M+H]^+^2031.8722, found: 2031.8665.

Compound **37**: To a solution of **35** (36 mg, 0.02 mmol) in AcOH/H_2_O (2 mL/0.5 mL), and stirred for 10 h at 40 °C. The reaction was quenched with saturated NaHCO_3_, then extracted with DCM and washed with brine. The organic phase was dried with anhydrous Na_2_SO_4_, and filtered and concentrated in vacuo to give crude product as a yellow oil, which could be used in the next reaction without purification.

To a solution of crude product in dry THF (2 mL), PPh_3_ (2 mg, 0.007 mmol), Pd(PPh_3_)_4_ (3 mg, 0.002 mmol), and Ammonium formate (3 mg, 0.05 mmol) were added at room temperature under a nitrogen atmosphere. After being stirred for 3 h, the mixture was filtered through celite and concentrated in vacuo. The residue was purified using flash chromatography (DCM/MeOH = 40:1, *v*/*v*) to afford white solid compound **37** (25 mg, 78% for two steps), R*_f_* = 0.32 (DCM/MeOH = 15:1, *v*/*v*). ^1^H NMR (400 MHz, Methanol-*d*_4_): *δ* 8.06 (d, *J* = 7.1 Hz, 2H, Ar-*H*), 7.64 (t, *J* = 7.4 Hz, 1H, Ar-*H*), 7.50 (t, *J* = 7.8 Hz, 2H, Ar-*H*), 7.41 (d, *J* = 7.3 Hz, 2H, Ar-*H*), 7.38–7.10 (m, 26H, Ar-*H*), 5.34 (t, *J* = 7.9 Hz, 1H, *H-2*), 5.23 (d, *J* = 3.8 Hz, 1H, *Fuc-H-1‴*), 5.13 (d, *J* = 7.8 Hz, 2H, Cbz-*CH_2_*), 4.88 (d, *J* = 7.9 Hz, 1H, *Glu-H-1*), 4.81 (d, *J* = 3.6 Hz, 1H, *Fuc-H-1″*), 4.74 (d, *J* = 11.9 Hz, 1H, Ph-C*H_2_*), 4.68–4.59 (m, 3H, Ph-C*H_2_*), 4.46 (s, 2H, N-Ph*CH_2_*), 4.41 (d, *J* = 11.6 Hz, 1H, Ph-*CH_2_*), 4.38–4.32 (m, 1H, *Gal-H-1′*), 4.26 (dd, *J* = 18.1, 10.4 Hz, 3H, Ph-C*H_2_*, *H-3′*, *H-3*), 4.13 (s, 1H, *H-4′*), 4.01 (p, *J* = 6.9 Hz, 2H, *H-5‴*, *H-5″*), 3.92 (t, *J* = 8.9 Hz, 1H, *H-4*), 3.90–3.74 (m, 8H, *H-3‴*, *H-3″*, *H-5*, *H-6a′*, *H-2′*, COO*Me*), 3.75–3.66 (m, 2H, *H-4″*, *H-2″*), 3.65 (dd, *J* = 10.9, 4.9 Hz, 2H, *H-5′*, OC*H_2_-a*), 3.47 (dd, *J* = 8.6, 4.6 Hz, 1H, *H-6b′*), 3.43 (d, *J* = 3.8 Hz, 1H, *H-2‴*), 3.27 (d, *J* = 2.9 Hz, 2H, *H-4‴*, OC*H_2_-b*), 3.22–3.11 (m, 2H, NC*H_2_*), 1.34–1.25 (m, 4H, OCH_2_C*H_2_*, C*H_2_*CH_2_N), 1.21 (d, *J* = 6.5 Hz, 3H, *H-6″*), 1.19–1.16 (m, 2H, OCH_2_CH_2_C*H_2_*), 0.85 (d, *J* = 6.5 Hz, 3H, *H-6‴*). ^13^C NMR (150 MHz, Methanol-*d*_4_): *δ* 171.2, 166.2, 140.2, 139.3, 139.1, 134.9, 131.3, 131.0, 130.0, 129.6, 129.4, 129.2, 129.2, 129.2, 128.9, 128.9, 128.8, 128.7, 128.6, 128.3, 103.3, 102.6, 98.7, 97.3, 82.1, 79.7, 78.0, 76.6, 75.9, 75.3, 74.0, 73.8, 73.7, 73.5, 73.3, 70.9, 70.6, 70.0, 68.9, 68.4, 67.5, 67.4, 67.0, 54.8, 53.5, 52.8, 51.4, 47.5, 33.1, 30.7, 30.5, 30.2, 28.8, 24.1, 23.7, 22.6, 16.7, 16.2, 14.4. HRMS (ESI) *m*/*z* calcd for C_75_H_94_N_3_O_23_ [M+NH_4_]^+^1404.6273, found: 1404.6294.

Compound **38**: To a solution of **36** (31 mg, 0.01 mmol) in DCM/Phosphate Buffered Saline (PBS, pH 7.4) (2 mL/0.11 mL), DDQ (21 mg, 0.09 mmol) was added at room temperature. The reaction mixture was stirred for 1 h and then extracted with DCM. The organic phase was washed with saturated NaHCO_3_ and brine, dried with anhydrous Na_2_SO_4_, and filtered and concentrated in vacuo to give crude product as a yellow oil, which could be used in the next reaction without purification.

To a solution of this crude product in dry THF (2 mL), PPh_3_ (1.2 mg, 0.005 mmol), Pd(PPh_3_)_4_ (1.8 mg, 0.002 mmol), and Ammonium formate (2 mg, 0.03 mmol) were added at room temperature under a nitrogen atmosphere. After stirring for 7 h, the mixture was filtered through celite and concentrated in vacuo. The residue was purified using flash chromatography (DCM/MeOH = 30:1, *v*/*v*) to afford white solid compound **38** (12.7 mg, 60% for two steps), R*_f_* = 0.23 (DCM/MeOH = 20:1, *v*/*v*). ^1^H NMR (500 MHz, Methanol-*d*_4_): *δ* 8.07–8.02 (m, 1H, Ar-*H*), 7.61 (t, *J* = 7.4 Hz, 1H, Ar-*H*), 7.51–7.42 (m, 4H, Ar-*H*), 7.40 (d, *J* = 7.5 Hz, 2H, Ar-*H*), 7.38–7.10 (m, 17H, Ar-*H*), 5.31 (t, *J* = 7.6 Hz, 1H, *H-2*), 5.17–5.09 (m, 2H, Cbz-*CH_2_*), 5.10 (d, *J* = 3.9 Hz, 1H, *Fuc-H-1‴*), 4.95 (d, *J* = 7.3 Hz, 1H, *Glu-H-1*), 4.83 (d, *J* = 3.8 Hz, 1H, *Fuc-H-1″*), 4.76 (d, *J* = 11.9 Hz, 1H, Ph-C*H_2_*), 4.70 (d, *J* = 11.8 Hz, 1H, Ph-C*H_2_*), 4.65 (d, *J* = 12.1 Hz, 1H, Ph-C*H_2_*), 4.59 (dd, *J* = 11.4, 6.1 Hz, 2H, Ph-C*H_2_*), 4.52 (d, *J* = 11.0 Hz, 1H, Ph-C*H_2_*), 4.47 (s, 2H, N-Ph*CH_2_*), 4.38 (d, *J* = 5.9 Hz, 1H, *Gal-H-1′*), 4.27 (d, *J* = 8.7 Hz, 1H, *H-3′*), 4.19 (t, *J* = 8.2 Hz, 1H, *H-3*), 4.11 (s, 1H, *H-4′*), 4.00 (q, *J* = 6.7, 6.1 Hz, 1H, *H-5″*), 3.96–3.90 (m, 2H, *H-2″*, *H-4*), 3.90–3.78 (m, 5H, *H-5*, *H-4″*, *H-2′*, *H-5‴*, *H-6a′*), 3.76–3.70 (m, 2H, *H-5′*, OC*H_2_-a*), 3.70–3.61 (m, 6H, COO*Me*, *H-2‴*, *H-3″*, *H-6b′*), 3.49 (dd, *J* = 10.0, 3.1 Hz, 1H, *H-3‴*), 3.46–3.42 (m, 1H, *H-4‴*), 3.39–3.33 (m, 1H, OC*H_2_-b*), 3.24–3.14 (m, 2H, NC*H_2_*), 1.31–1.29 (m, 4H, OCH_2_C*H_2_*, C*H_2_*CH_2_N), 1.28–1.25 (m, 2H, OCH_2_CH_2_C*H_2_*), 1.23 (d, *J* = 6.6 Hz, 3H, *H-6″*), 0.82 (d, *J* = 6.5 Hz, 3H, *H-6‴*). ^13^C NMR (125 MHz, Methanol-*d*_4_): *δ* 171.1, 166.3, 140.2, 140.1, 139.0, 134.6, 131.2, 131.0, 129.7, 129.6, 129.4, 129.4, 129.3, 129.1, 129.0, 128.9, 128.9, 128.7, 128.6, 128.5, 128.4, 103.0, 102.6, 100.9, 99.8, 81.9, 79.8, 79.4, 78.6, 76.7, 75.7, 75.5, 74.1, 72.6, 72.5, 70.6, 70.6, 70.1, 69.2, 68.9, 68.8, 68.4, 67.8, 67.6, 53.4, 52.8, 51.3, 47.5, 33.1, 31.8, 30.8, 30.8, 30.7, 30.6, 30.5, 30.3, 30.2, 24.2, 23.7, 22.7, 16.8, 16.3, 14.4. HRMS (ESI) *m*/*z* calcd for C_75_H_94_N_3_O_23_ [M+NH_4_]^+^ 1404.6273, found 1404.6259.

5-amino-pentanyl(6-*O*-(3,4-di-*O*-sulfo-α-L-fucopyranoside)-4-*O*-sulfo-3-*O*-(3-*O*-(3,4-di-*O*-sulfo-α-L-fucopyranoside)-β-D-glucopyranosyluronate)-2-acetamino-2-deoxy-β-D-galactopyranoside) **(FCS-1)**.

To a solution of **37** (22 mg, 0.02 mmol) in dry DMF (1.5 mL), SO_3_·Me_3_N (221 mg, 1.59 mmol) was added at room temperature. The reaction mixture was heated to 70 °C in a microwave synthesizer and stirred for 2 h. Et_3_N and MeOH quenched the reaction, and it was concentrated in vacuo to give crude product as yellow oil. This product could be used in the next step without purification.

This product was dissolved in THF/H_2_O (1.6 mL/0.2 mL), and LiOH aqueous solution (1 M, 1 mL) was added. The reaction mixture was stirred overnight. After being concentrated in vacuo, it was dissolved in MeOH/DCM (1.1 mL/0.2 mL), and a NaOH aqueous solution (0.5 M, 2 mL) was added. The reaction was stirred for 8 h and the pH was adjusted to neutral by the addition of IR-120 H^+^ cation exchange resin. It was concentrated in vacuo to give crude product as yellow oil. This product could be used in the next step without purification.

To a solution of this crude product in H_2_O/MeOH (1 mL/1.5 mL), Pd/C (10%, 190 mg) and Pd(OH)_2_/C (10%, 190 mg) were added at 30 °C under a hydrogen atmosphere. After being stirred for 12 h, the mixture was filtered through celite and concentrated in vacuo. The residue was exchanged with Amberlite IR-120 (Na^+^) ion-exchange resin and purified with Sephadex LH-20 (CH_3_OH) to give the white solid compound **FCS-1** (13 mg, 63% for three steps), R*_f_* = 0.16 (EtOAc/EtOH/H_2_O = 2:1:1, *v*/*v*). ^1^H NMR (400 MHz, D_2_O): *δ* 5.33 (d, *J* = 4.0 Hz, 1H, *Fuc-H-1‴*), 5.04 (d, *J* = 3.9 Hz, 1H, *Fuc-H-1″*), 4.85 (t, *J* = 3.4 Hz, 2H, *H-4‴*, *H-4″*), 4.76 (s, 1H, *H-4′*), 4.57 (ddd, *J* = 16.3, 10.5, 2.9 Hz, 2H, *H-3‴*, *H-3″*), 4.48 (dt, *J* = 11.5, 6.0 Hz, 3H, *Gal-H-1′*, *Glu-H-1*, *H-5‴*), 4.21 (q, *J* = 6.6 Hz, 1H, *H-5″*), 4.08–3.98 (m, 3H, *H-3′*, *H-2′*, *H-5′*), 3.92 (dt, *J* = 9.4, 5.2 Hz, 4H, *H-2‴*, *H-2″*, *H-6′*), 3.86 (dd, *J* = 11.3, 5.0 Hz, 1H, OC*H_2_-a*), 3.68–3.59 (m, 3H, *H-5*, *H-4*, OC*H_2_-b*), 3.59–3.55 (m, 2H, *H-2*, *H-3*), 2.00 (s, 3H, Ac-C*H_3_*), 1.62 (dp, *J* = 20.9, 7.1, 6.6 Hz, 4H, OCH_2_C*H_2_*, C*H_2_*CH_2_N), 1.39 (p, *J* = 7.5, 6.9 Hz, 2H, OCH_2_CH_2_C*H_2_*), 1.27 (d, *J* = 6.6 Hz, 3H, *H-6″*), 1.22 (d, *J* = 6.5 Hz, 3H, *H-6‴*). ^13^C NMR (100 MHz, D_2_O): *δ* 103.2(C-1), 101.2(C-1′), 99.8(C-1″), 98.8(C-1*‴*), 81.3(C-3), 79.1(C-4″), 78.8(C-4*‴*), 77.0(C-5), 76.5(C-4′), 75.3(C-3″, C-3*‴*), 74.9(C-3′), 74.3, 73.6(C-5′), 73.0(C-2), 70.2(O-CH_2_), 70.1(C-4), 69.1(C-6′), 66.5(C-2″), 66.4(C-5*‴*), 66.3(C-5″), 66.1(C-5*‴*), 51.7(C-2′), 39.3(N-CH_2_), 28.0, 26.2, 22.2(Ac-CH_3_), 22.0, 16.0(C-6″), 15.8(C-6*‴*). HRMS (ESI) *m*/*z* calcd for C_31_H_50_N_2_O_35_S_5_ [M-6Na+2H]^4−^ 292.5205, found 292.5213.

5-amino-pentanyl(6-*O*-(2,4-di-*O*-sulfo-α-L-fucopyranoside)-4-*O*-sulfo-3-*O*-(3-*O*-(2,4-di-*O*-sulfo-α-L-fucopyranoside)-β-D-glucopyranosyluronate)-2-acetamino-2-deoxy-β-D-galactopyranoside) (**FCS-2**).

Following the synthesis of compound **FCS-1**, compound **38** (23 mg, 0.02 mmol) was converted to the corresponding product, which was purified with Sephadex LH-20 (CH_3_OH) to give the white solid compound **FCS-2** (14 mg, 65% for three steps), R*_f_* = 0.16 (EtOAc/EtOH/H_2_O = 2:1:1, *v*/*v*). ^1^H NMR (500 MHz, D_2_O): *δ* 5.55 (d, *J* = 3.6 Hz, 1H, *Fuc-H-1‴*), 5.34 (d, *J* = 3.4 Hz, 1H, *Fuc-H-1″*), 4.88 (s, 1H, *H-4′*), 4.67 (dd, *J* = 5.4, 2.1 Hz, 2H, *H-4″*, *H-4‴*), 4.54 (dq, *J* = 13.5, 6.8, 5.6 Hz, 3H, *Glu-H-1*, *Gal-H-1′*, *H-5‴*), 4.42 (ddd, *J* = 17.7, 10.4, 3.4 Hz, 2H, *H-2″*, *H-2‴*), 4.25 (q, *J* = 6.3 Hz, 1H, *H-5″*), 4.12 (ddd, *J* = 13.8, 10.5, 2.7 Hz, 2H, *H-3‴*, *H-3″*), 4.04–3.92 (m, 4H, *H-2′*, *H-3′*, *H-5′*, *H-3*), 3.95–3.90 (m, 1H, *H-6a′*), 3.91–3.86 (m, 1H, OC*H_2_-a*), 3.82 (dd, *J* = 12.1, 8.1 Hz, 1H, *H-6b′*), 3.78–3.71 (m, 2H, *H-4*, *H-5*), 3.62 (ddt, *J* = 14.5, 8.9, 3.6 Hz, 4H, *H-2*, *H-3*, OC*H_2_-b*), 2.99 (t, *J* = 7.4 Hz, 2H, NC*H_2_*), 2.01 (s, 3H, Ac-C*H_3_*), 1.67 (dt, *J* = 15.3, 7.8 Hz, 2H, OCH_2_C*H_2_*), 1.61 (dt, *J* = 14.1, 6.3 Hz, 2H, C*H_2_*CH_2_N), 1.41 (dt, *J* = 15.4, 7.3 Hz, 2H, OCH_2_CH_2_C*H_2_*), 1.28 (d, *J* = 6.5 Hz, 3H, *H-6″*), 1.24 (d, *J* = 6.5 Hz, 3H, *H-6‴*). ^13^C NMR (150 MHz, D_2_O): *δ* 103.9(C-1), 101.0(C-1′), 97.1(C-1*‴*), 96.9(C-1″), 81.4(C-3), 80.8(C-4″), 80.6(C-4*‴*), 77.3(C-3′), 77.2(C-5), 76.8(C-4), 75.2(C-1″), 75.1(C-1*‴*), 74.7(C-3), 73.7(C-5′), 72.8(C-2), 70.2(O-CH_2_), 69.3(C-4), 67.3(C-6′), 66.5(C-3″), 66.6(C-3*‴*), 66.2(C-5*‴*), 66.1(C-5″), 51.5(C-2′), 39.4(N-CH_2_), 28.0, 26.3, 22.3(Ac-CH_3_), 22.1, 15.9(C-6″), 15.6(C-6*‴*). HRMS (ESI) *m*/*z* calcd for C_31_H_51_N_2_O_35_S_5_ [M-6Na+3H]^3−^ 390.3620, found 390.3611.

## 4. Conclusions

In summary, we synthesized FCS tetrasaccharides **FCS-1** and **FCS-2** with fucosyl branches both at the 6-OH of GalNAc and the 3-OH of GlcA from commercially available monosaccharides through 27 collective steps. In the synthesis process, fucose branched chains with 1,2-*cis*-glycosidic bonds were constructed with excellent stereoselectivity, by utilizing Nap and isopropylidene groups, especially fucose coupled with glucuronic acid 3-OH. The selective sulfation of hydroxyl groups was achieved through the orthogonal protection strategy. Notably, this approach can also be utilized for sulfation modification at other hydroxyl sites, obtaining diverse FCS derivatives. Therefore, this research enriches knowledge of the types of FCS oligosaccharides, and will be beneficial to their biological and pharmacological evaluation and application.

## Figures and Tables

**Table 1:** Glycosylation of disaccharide acceptor **28** with fucose donor **18**.

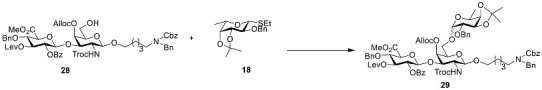						
Entry	Donor (eq)	Acceptor (eq)	T (°C)	Activator (eq)	Yield *^a^* (%)	α:β *^b^*
1	**18**(1.2)	**28**(1.0)	−25	NIS (1.5) TfOH (0.3)	78	1.1:1
2	**18**(1.2)	**28**(1.0)	−45	NIS (1.5) TfOH (0.3)	62	1.25:1
3	**18**(1.2)	**28**(1.0)	−15	NIS (1.5) TfOH (0.3)	83	1.3:1
4	**18**(1.5)	**28**(1.0)	−15	NIS (1.5) TfOH (0.3)	90	1.8:1
5	**18**(1.5)	**28**(1.0)	−15	NIS (1.5) TMSOTf (0.3)	86	1.5:1
6	**18**(1.5)	**28**(1.0)	−15	NIS (1.5) TBSOTf (0.3)	88	1.6:1

*^a^* Combined yield of α- and β-isomers. *^b^* Determined from the masses of the isolated and purified α- and β-isomer products.

## Data Availability

The data are contained within the article or Supplementary Materials.

## Supplementary Materials

The following supporting information can be downloaded at: https://www.mdpi.com/article/10.3390/md22040184/s1, Figures S1–S17: ^1^H NMR and ^13^C NMR spectrum of compounds **19**–**38**, **FCS-1** and **FCS-2**.

## References

[1] Pomin V.H. (2014). Holothurian Fucosylated Chondroitin Sulfate. Mar. Drugs.

[2] Wu M.Y., Huang R., Wen D.D., Gao N., He J.B., Li Z., Zhao J.H. (2012). Structure and Effect of Sulfated Fucose Branches on Anticoagulant Activity of the Fucosylated Chondroitin Sulfate from Sea Cucumber Thelenata Ananas. Carbohydr. Polym..

[3] Chen S.G., Xue C.H., Yin L., Tang Q.J., Yu G.L., Chai W.G. (2011). Comparison of Structures and Anticoagulant Activities of Fucosylated Chondroitin Sulfates from Different Sea Cucumbers. Carbohydr. Polym..

[4] Ustyuzhanina N.E., Bilan M.I., Dmitrenok A.S., Tsvetkova E.A., Shashkov A.S., Stonik V.A., Nifantiev N.E., Usov A.I. (2016). Structural Characterization of Fucosylated Chondroitin Sulfates from Sea Cucumbers Apostichopus Japonicus and Actinopyga Mauritiana. Carbohydr. Polym..

[5] Ustyuzhanina N.E., Bilan M.I., Dmitrenok A.S., Shashkov A.S., Nifantiev N.E., Usov A.I. (2017). The Structure of a Fucosylated Chondroitin Sulfate from the Sea Cucumber Cucumaria Frondosa. Carbohydr. Polym..

[6] Li Q.Y., Cai C., Chang Y.G., Zhang F.M., Linhardt R.J., Xue C.H., Li G.Y., Yu G.L. (2018). A Novel Structural Fucosylated Chondroitin Sulfate from Holothuria Mexicana and Its Effects on Growth Factors Binding and Anticoagulation. Carbohydr. Polym..

[7] Panagos C.G., Thomson D.S., Moss C., Hughes A.D., Kelly M.S., Liu Y., Chai W., Venkatasamy R., Spina D., Page C.P. (2014). Fucosylated Chondroitin Sulfates from the Body Wall of the Sea Cucumber Holothuria Forskali Conformation, Selectin Binding, and Biological Activity. J. Biol. Chem..

[8] Borsig L., Wang L., Cavalcante M.C.M., Cardilo-Reis L., Ferreira P.L., Mourao P.A.S., Esko J.D., Pavao M.S.G. (2007). Selectin Blocking Activity of a Fucosylated Chondroitin Sulfate Glycosaminoglycan from Sea Cucumber Effect on Tumor Metastasis and Neutrophil Recruitment. J. Biol. Chem..

[9] Gao N., Wu M.Y., Liu S., Lian W., Li Z., Zhao J.H. (2012). Preparation and Characterization of O-Acylated Fucosylated Chondroitin Sulfate from Sea Cucumber. Mar. Drugs.

[10] Tovar A.M.F., Mourão P.A.S. (1996). High Affinity of a Fucosylated Chondroitin Sulfate for Plasma Low Density Lipoprotein. Atherosclerosis.

[11] Tapon-Bretaudière J., Drouet B., Matou S., Mourão P.A.S., Bros A., Letourneur D., Fischer A.M. (2000). Modulation of Vascular Human Endothelial and Rat Smooth Muscle Cell Growth by a Fucosylated Chondroitin Sulfate from Echinoderm. Thromb. Haemost..

[12] Zhang X., Liu H.Y., Lin L.S., Yao W., Zhao J.H., Wu M.Y., Li Z.J. (2018). Synthesis of Fucosylated Chondroitin Sulfate Nonasaccharide as a Novel Anticoagulant Targeting Intrinsic Factor Xase Complex. Angew. Chem. Int. Ed..

[13] Sun H.F., Gao N., Ren L., Liu S., Lin L.S., Zheng W.Q., Zhou L., Yin R.H., Zhao J.H. (2020). The Components and Activities Analysis of a Novel Anticoagulant Candidate dHG-5. Eur. J. Med. Chem..

[14] Lan D., Zhang J.L., Shang X.L., Yu L.J., Xu C., Wang P., Cui L.G., Cheng N.Q., Sun H.F., Ran J.N. (2023). Branch Distribution Pattern and Anticoagulant Activity of a Fucosylated Chondroitin Sulfate from Phyllophorella Kohkutiensis. Carbohydr. Polym..

[15] Wu M.Y., Xu S.M., Zhao J.H., Kang H., Ding H. (2010). Preparation and Characterization of Molecular Weight Fractions of Glycosaminoglycan from Sea Cucumber Thelenata Ananas Using Free Radical Depolymerization. Carbohydr. Res..

[16] Gao N., Lu F., Xiao C., Yang L., Chen J., Zhou K., Wen D.D., Li Z., Wu M.Y., Jiang J.M. (2015). β-Eliminative Depolymerization of the Fucosylated Chondroitin Sulfate and Anticoagulant Activities of Resulting Fragments. Carbohydr. Polym..

[17] Tamura J., Tanaka H., Nakamura A., Takeda N. (2013). Synthesis of β-d-GalNAc(4,6-diS)(1–4)[α-l-Fuc(2,4-diS)(1–3)]-β-d-GlcA, a Novel Trisaccharide Unit of Chondroitin Sulfate with a Fucose Branch. Tetrahedron Lett..

[18] He H.Q., Chen D., Li X.M., Li C.J., Zhao J.H., Qin H.B. (2019). Synthesis of Trisaccharide Repeating Unit of Fucosylated Chondroitin Sulfate. Org. Biomol. Chem..

[19] Zhang L.Z., Xu P., Liu B.Z., Yu B. (2020). Chemical Synthesis of Fucosylated Chondroitin Sulfate Oligosaccharides. J. Org. Chem..

[20] Zhou X., Long Q., Li D.W., Gao J.R., Sun Q.K., Sun S.Z., Su Y., Wang P., Peng W.J., Li M. (2021). Convergent Synthesis of Branched β-Glucan Tridecasaccharides Ready for Conjugation. Synthesis.

[21] Shen K., Bai B., Liu Y.H., Lowary T.L. (2021). Synthesis of a Tridecasaccharide Lipooligosaccharide Antigen from the Opportunistic Pathogen Mycobacterium Kansasii. Angew. Chem. Int. Ed..

[22] Zhang Y.Q., Xiang G.S., He S.J., Hu Y.K., Liu Y.J., Xu L.L., Xiao G.Z. (2019). Orthogonal One-Pot Synthesis of Oligosaccharides Based on Glycosyl Ortho-Alkynylbenzoates. Org. Lett..

[23] Nitz M., Bundle D.R. (2000). Efficient Synthesis of 3,6-Dideoxy-Beta-D-Arabino-Hexopyranosyl-Terminated LacdiNac Glycan Chains of the Trichinella Spiralis Parasite. J. Org. Chem..

[24] Castelli R., Overkleeft H.S., van der Marel G.A., Codée J.D.C. (2013). 2,2-Dimethyl-4-(4-Methoxy-Phenoxy) Butanoate and 2,2-Dimethyl-4-Azido Butanoate: Two New Pivaloate-Ester-like Protecting Groups. Org. Lett..

[25] Pham J., Hernandez A., Cioce A., Achilli S., Goti G., Vivès C., Thepaut M., Bernardi A., Fieschi F., Reichardt N.C. (2020). Chemo-Enzymatic Synthesis of S. Mansoni O-Glycans and Their Evaluation as Ligands for C-Type Lectin Receptors MGL, DC-SIGN, and DC-SIGNR. Chem. Eur. J..

[26] Lv C.L., Su K.Q., Li Y., Chen H.J. (2020). Synthesis of fucosylated chondroitin sulfate trisaccharide. Chin. J. Mar. Drugs.

[27] Lampe T.F.J., Weitz-Schmidt G., Wong C.H. (1998). Parallel Synthesis of Sialyl Lewis X Mimetics on a Solid Phase: Access to a Library of Fucopeptides. Angew. Chem. Int. Ed..

[28] Lönn H. (1985). Synthesis of a Tetra- and a Nona-Saccharide Which Contain α-l-Fucopyranosyl Groups and Are Part of the Complex Type of Carbohydrate Moiety of Glycoproteins. Carbohydr. Res..

[29] Van den Bos L.J., Codée J.D.C., van der Toorn J.C., Boltje T.J., van Boom J.H., Overkleeft H.S., van der Marel G.A. (2004). Thioglycuronides: Synthesis and Application in the Assembly of Acidic Oligosaccharides. Org. Lett..

[30] Gagarinov I.A., Fang T., Liu L., Srivastava A.D., Boons G.J. (2015). Synthesis of Staphylococcus Aureus Type 5 Trisaccharide Repeating Unit: Solving the Problem of Lactamization. Org. Lett..

