# Supplementary material for: Chemical Synthesis of Fucosylated Chondroitin Sulfate Tetrasaccharide with Fucosyl Branch at the 6-OH of GalNAc Residue
Source: Mar Drugs. 2024 Apr 19;22(4):184. doi: 10.3390/md22040184 (PMC11051034; doi:10.3390/md22040184)
Supplement: Supplementary file 1 [file marinedrugs-22-00184-s001.zip › marinedrugs-2952374-supplementary.pdf]

Chemical Synthesis of Fucosylated Chondroitin Sulfate Tetrasaccharide with Fucosyl Branch at the 6-OH of GalNAc Residue

Changlun Lv[1], Xiaona Li[1], Guoqing Yang[1], Haijiao Chen[1], Chunxia Li [1,2] *

1.Key Laboratory of Marine Drugs, Ministry of Education, Shandong Provincial Key Laboratory of Glycoscience and Glycotechnology, School of Medicine and Pharmacy, Ocean University of China, Qingdao 266003, China

2. Laboratory of Marine Drugs and Bioproducts of Qingdao Pilot National Laboratory for Marine Science and Technology, Qingdao 266237, China)

* Corresponding author. Email address: lchunxia@ouc.edu.cn (Chunxia Li).

Contents of Supporting Information

Experimental Procedures of compound **19** and **20**..S3

Figure S1. ^{1}H NMR and ^{13}C NMR spectrum of compound **19**..S5

Figure S2. ^{1}H NMR and ^{13}C NMR spectrum of compound **20**..S6

Figure S3. ^{1}H NMR and ^{13}C NMR spectrum of compound **21**..S7

Figure S4. ^{1}H NMR and ^{13}C NMR spectrum of compound **22**..S8

Figure S5. ^{1}H NMR and ^{13}C NMR spectrum of compound **24**..S9

Figure S6. ^{1}H NMR and ^{13}C NMR spectrum of compound **25**..S10

Figure S7. ^{1}H NMR and ^{13}C NMR spectrum of compound **26**..S11

Figure S8. ^{1}H NMR and ^{13}C NMR spectrum of compound **27**..S12

Figure S9. ^{1}H NMR and ^{13}C NMR spectrum of compound **29**..S13

Figure S10. ^{1}H NMR and ^{13}C NMR spectrum of compound **30**..S14

Figure S11. ^{1}H NMR and ^{13}C NMR spectrum of compound **34**..S15

Figure S12. ^{1}H NMR and ^{13}C NMR spectrum of compound **35**..S16

Figure S13. ^{1}H NMR and ^{13}C NMR spectrum of compound **36**..S17

Figure S14. ^{1}H NMR and ^{13}C NMR spectrum of compound **37**..S18

Figure S15. ^{1}H NMR and ^{13}C NMR spectrum of compound **38**..S19

Figure S16. ^{1}H NMR and ^{13}C NMR spectrum of compound **FCS-1**...S20

Figure S17. ^{1}H NMR and ^{13}C NMR spectrum of compound **FCS-2**...S21

Experimental Procedures

1-Ethylthio-3-*O*-benzyl-β-L-fucopyranoside (19)

To a solution of **16**[5] (100 mg, 0.48 mmol) in dry toluene (5 mL) was added Bu_2SnO (240 mg, 0.96 mmol) under nitrogen atmosphere. The reaction mixture was heated under reflux for 5 h, then concentrated *in vacuo*. The obtained residue was dissolved in DMF (3 mL), and CsF (146 mg, 0.96 mmol), BnBr (86.2 μL, 0.72 mmol) were added and stirred at room temperature overnight. The reaction mixture was filtered through celite, extracted with DCM and washed with water and brine. The organic phase was dried with anhydrous Na_2SO_4, filtered and concentrated *in vacuo*. The residue was purified by flash chromatography (DCM/CH_3OH=30:1, v/v) to afford white solid compound **19** (127.6 mg, 89.1%). R_f =0.85（DCM/CH_3OH=10:1, v/v）。^1H NMR (500 MHz, $CDCl_3$): δ 7.38-7.26 (m, 5H), 4.76 (s, 2H), 4.26 (d, J = 9.7 Hz, 1H), 3.81-3.76 (m, 2H), 3.59 (q, J =6.1 Hz, 1H), 3.44-3.42 (dd, J = 8.7, 2.3 Hz, 1H), 2.79-2.68 (m, 2H), 2.47 (s, 1H), 2.34 (s, 1H), 1.34 (d, J = 6.2 Hz, 3H), 1.30 (t, J = 7.4 Hz, 3H). ^{13}C NMR (125 MHz, $CDCl_3$): δ 137.9, 128.7, 128.2, 128.0, 86.0, 81.8. 74.7, 72.2, 69.6, 69.3, 24.1, 16.9, 15.4. HRMS (ESI) *m/z* calcd for $C_{15}H_{23}O_4S$ $[M+H]^+$ 299.1317, found 299.1312.

1-Ethylthio-2,4-di-*O*-(2-naphthylmethyl)-3-*O*-benzyl-β-L-fucopyranoside (20)

To a solution of **19** (76 mg, 0.26 mmol) in dry DMF (2 mL) were added NaH (60% in mineral oil, 20 mg, 0.51 mmol) and NapBr (226 mg, 1.02 mmol) at 0 °C

under nitrogen atmosphere. After being warmed to room temperature, the mixture was stirred 5 h. The reaction was quenched with saturated NH_4Cl and then extracted with DCM. The organic phase was washed with water and brine, dried with anhydrous Na_2SO_4, filtered and concentrated *in vacuo*. The residue was purified by flash chromatography (PE/EtOAc=10:1, v/v) to afford white solid compound **20** (96 mg, 65%), R_f =0.78 (PE/EtOAc=2:1, v/v). ^{1}H NMR (400 MHz, $CDCl_3$) δ ^{1}H NMR (400 MHz, $CDCl_3$) δ 7.87-7.74 (m, 8H, Ar-*H*), 7.60-7.43 (m, 6H, Ar-*H*), 7.42-7.28 (m, 5H, Ar-*H*), 7.26 (s, 1H, Ar-*H*), 5.15 (d, *J* = 11.9 Hz, 1H, Nap-CH_2), 5.08 (d, *J* = 10.4 Hz, 1H, Nap-CH_2), 4.98 (d, *J* = 10.5 Hz, 1H, Nap-CH_2), 4.90 (d, *J* = 12.0 Hz, 1H, Nap-CH_2), 4.85-4.75 (m, 2H, PhCH_2), 4.45 (d, *J* = 9.6 Hz, 1H, *H-1*), 3.93 (t, *J* = 9.4 Hz, 1H, *H-2*), 3.68 (d, *J* = 2.3 Hz, 1H, *H-4*), 3.62 (dd, *J* = 9.3, 2.9 Hz, 1H, *H-3*), 3.51 (q, *J* = 6.2 Hz, 1H, *H-5*), 2.88-2.68 (m, 2H, SCH_2CH_3), 1.32 (t, *J* = 7.4 Hz, 3H, SCH_2CH_3), 1.24 (d, *J* = 6.4 Hz, 3H, *H-6*). ^{13}C NMR (100 MHz, $CDCl_3$): δ 138.7, 136.3, 136.1, 133.5, 133.3, 133.2, 133.1, 128.6, 128.1, 128.0, 127.8, 127.8, 127.7, 127.2, 127.0, 126.7, 126.6, 126.1, 126.0, 125.9, 85.2(C-1), 84.7(C-3), 78.6(C-2), 76.5(C-4), 75.9(Nap-CH_2), 74.7(C-5), 74.7(Nap-CH_2), 73.1(Ph-CH_2), 24.9(SCH_2CH_3), 17.5(C-6), 15.2($SCH_2\mathbf{C}H_3$). HRMS (ESI) *m/z* calcd for $C_{37}H_{42}O_4NS$ $[M+NH_4]^+$ 596.2829, found 596.2825.

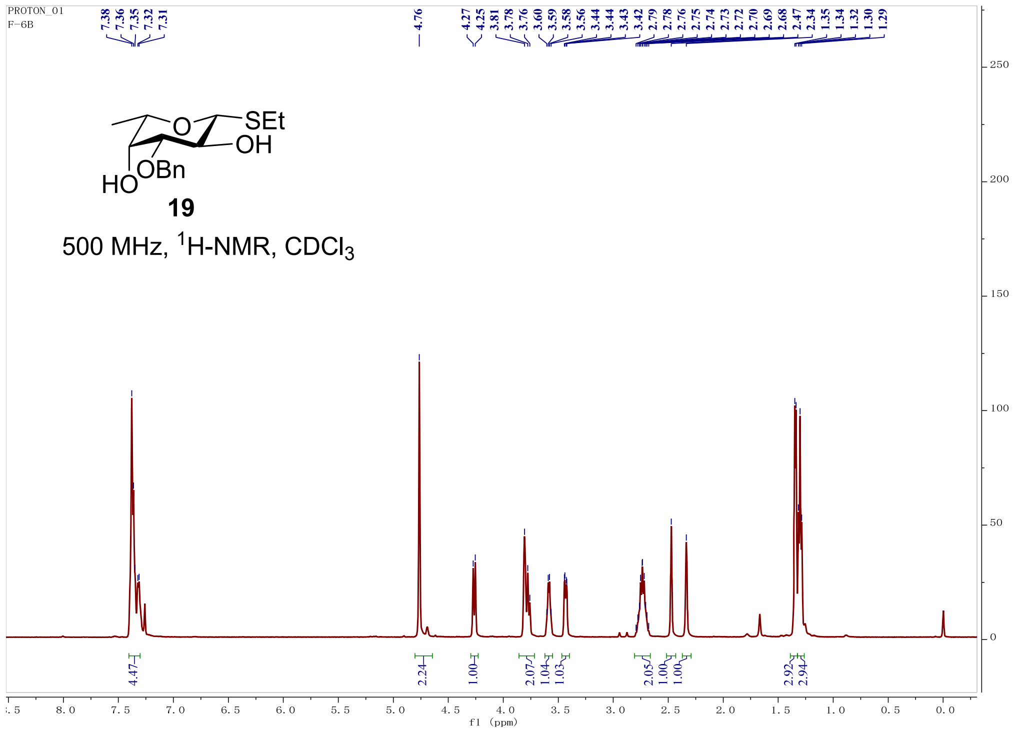

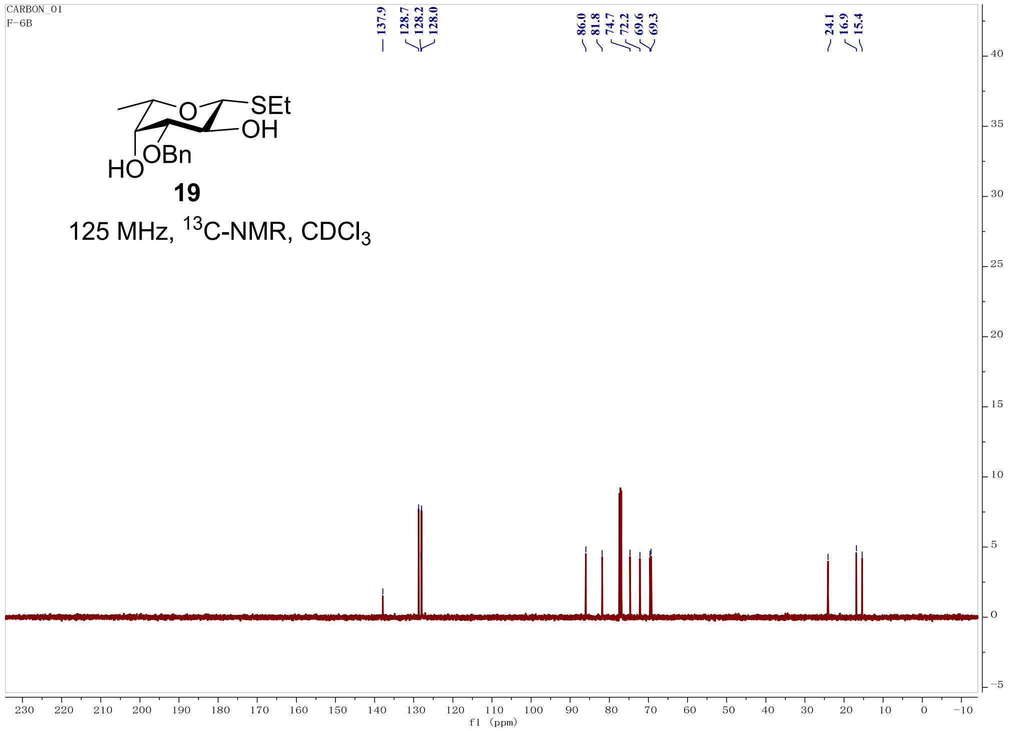

Figure S1. ^{1}H NMR and ^{13}C NMR spectrum of compound **19**.

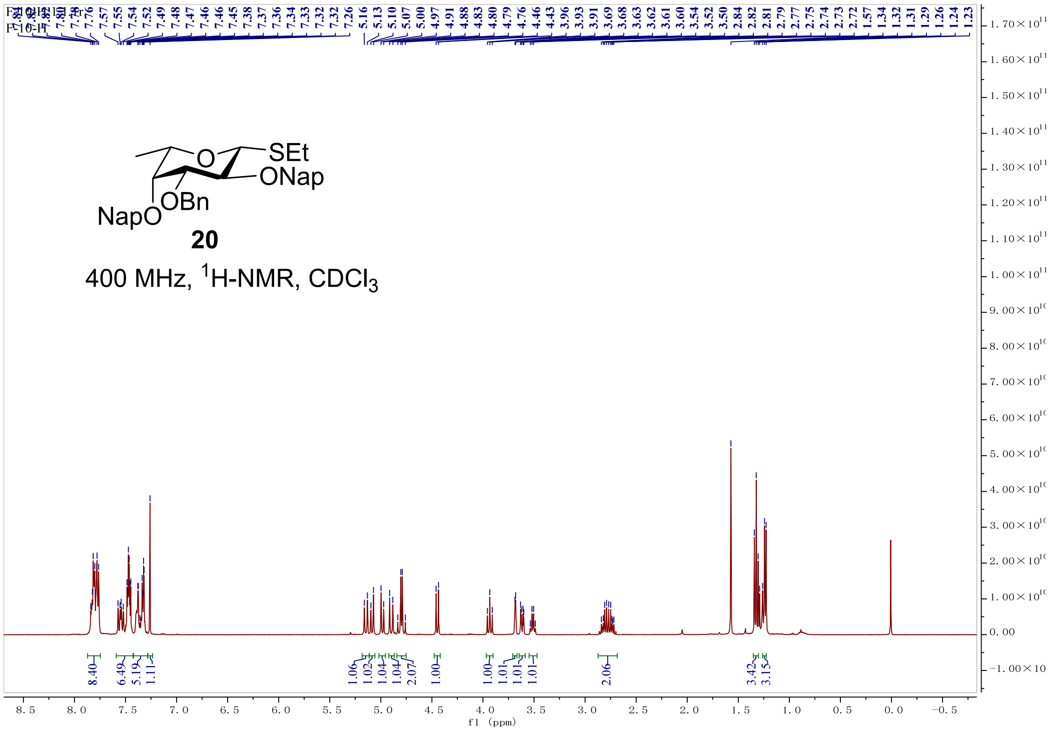

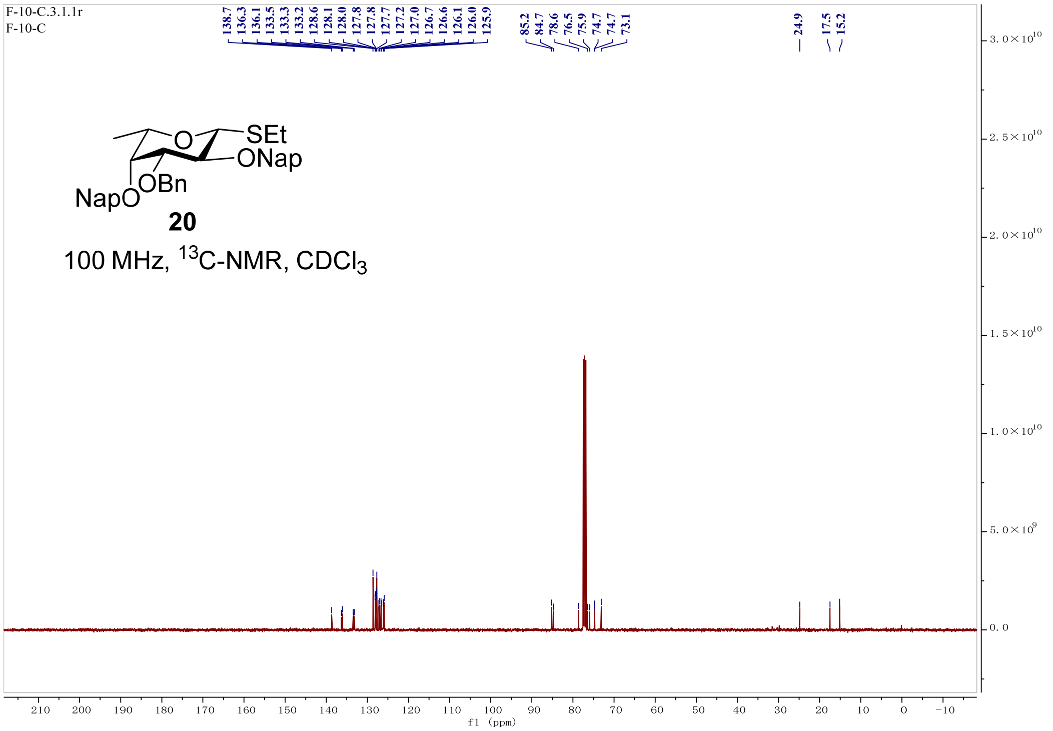

Figure S2. ^{1}H NMR and ^{13}C NMR spectrum of compound **20**.

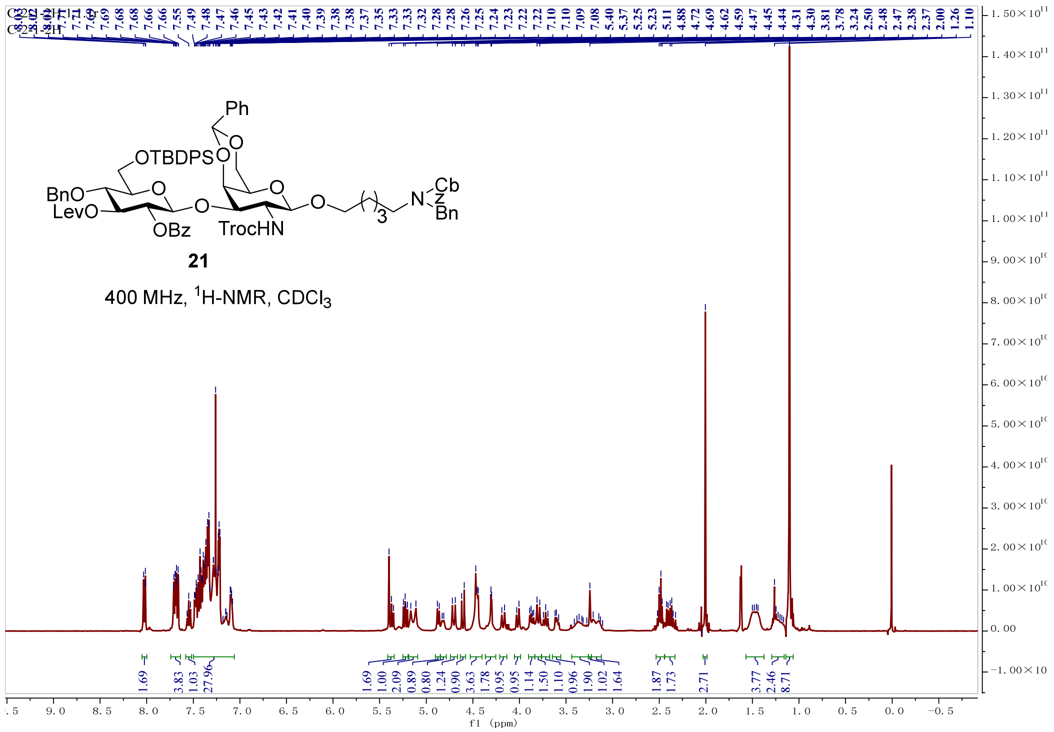

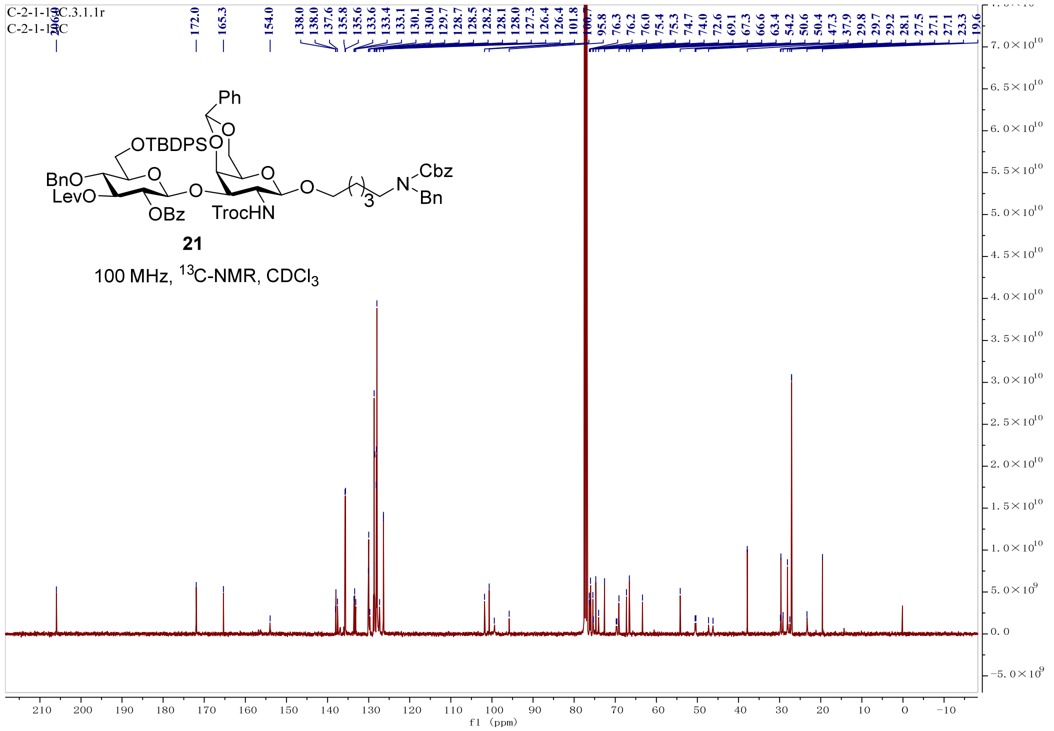

Figure S3. ^{1}H NMR and ^{13}C NMR spectrum of compound **21**.

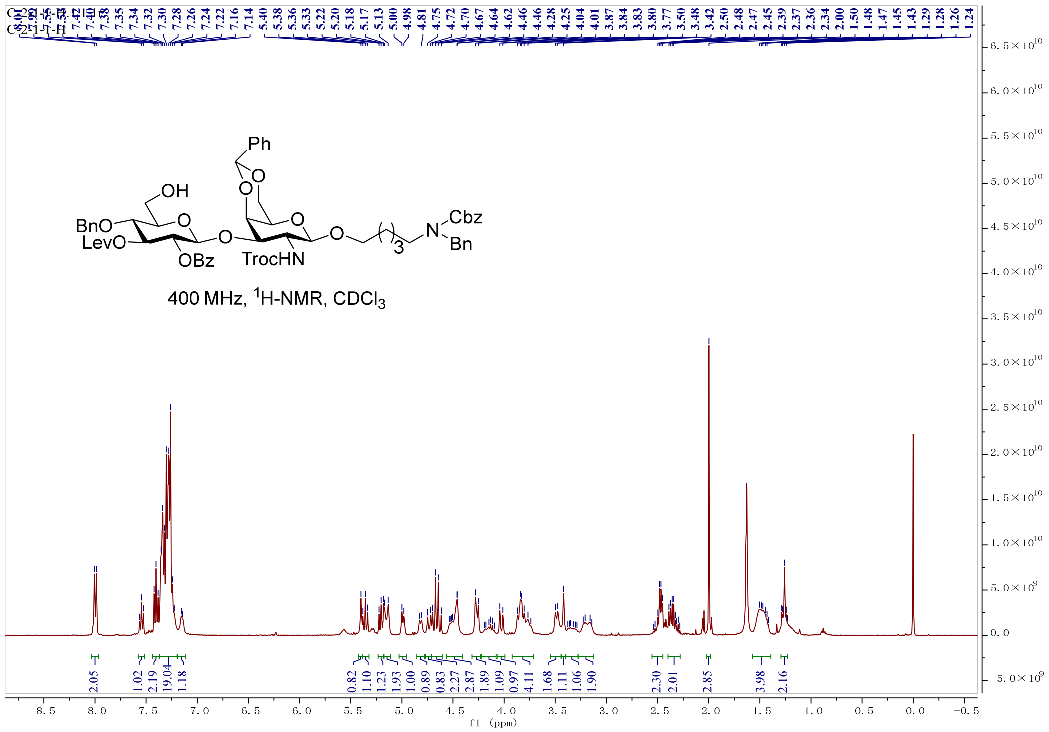

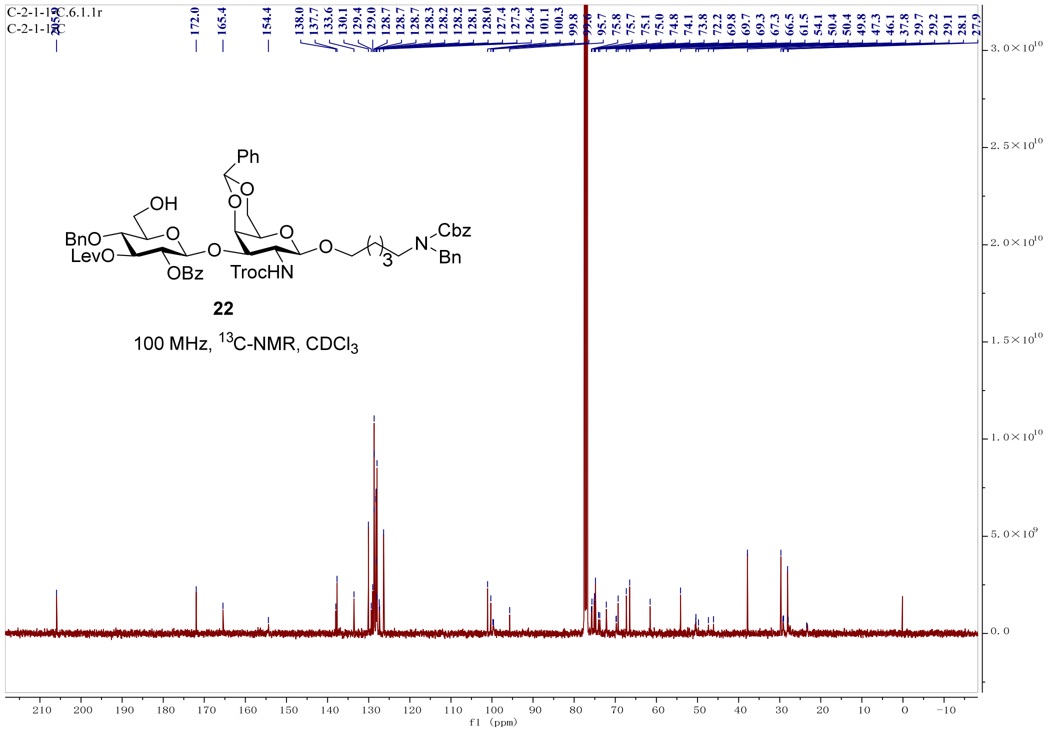

Figure S4. ¹H NMR and ¹³C NMR spectrum of compound **22**.

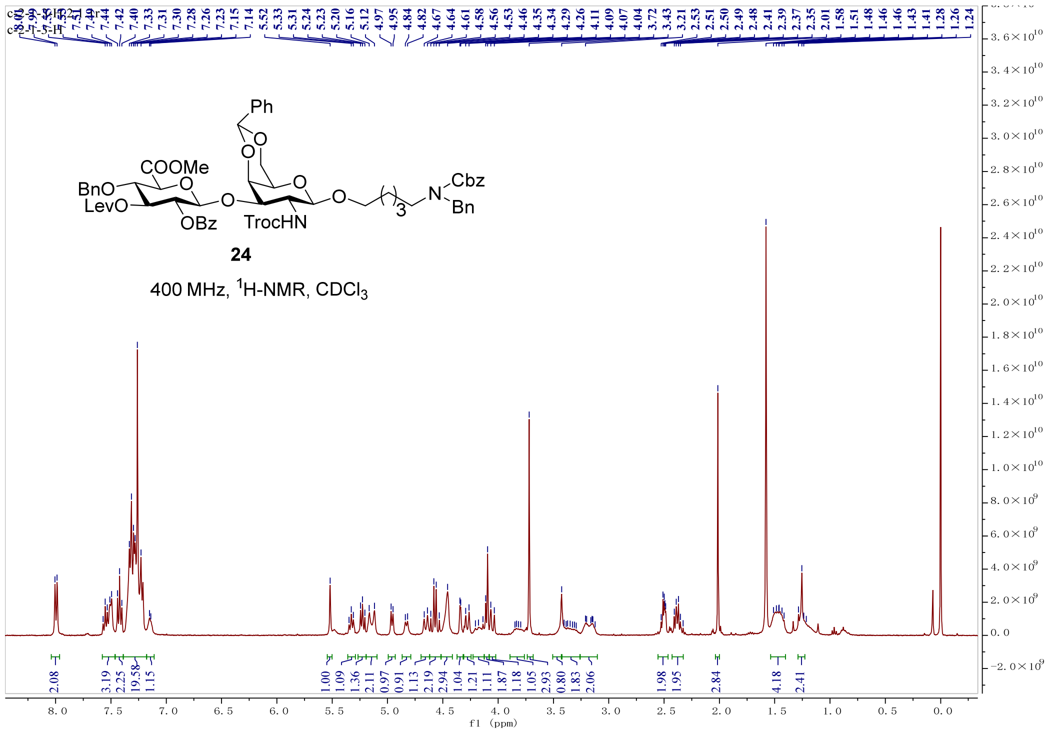

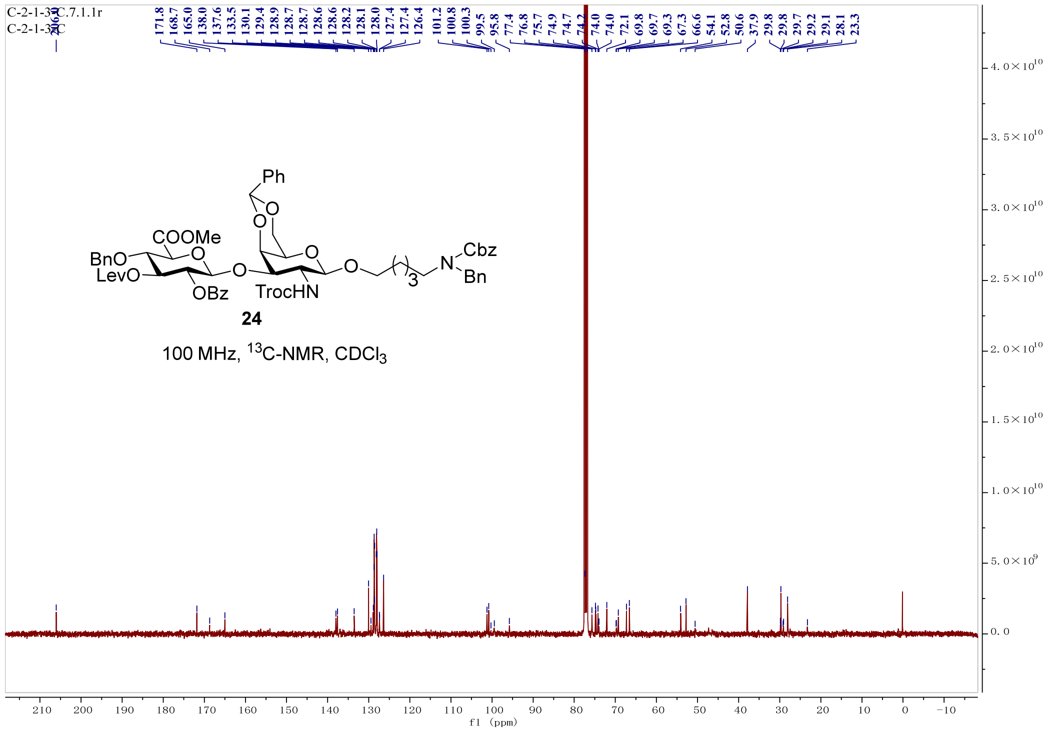

Figure S5. ^{1}H NMR and ^{13}C NMR spectrum of compound **24**.

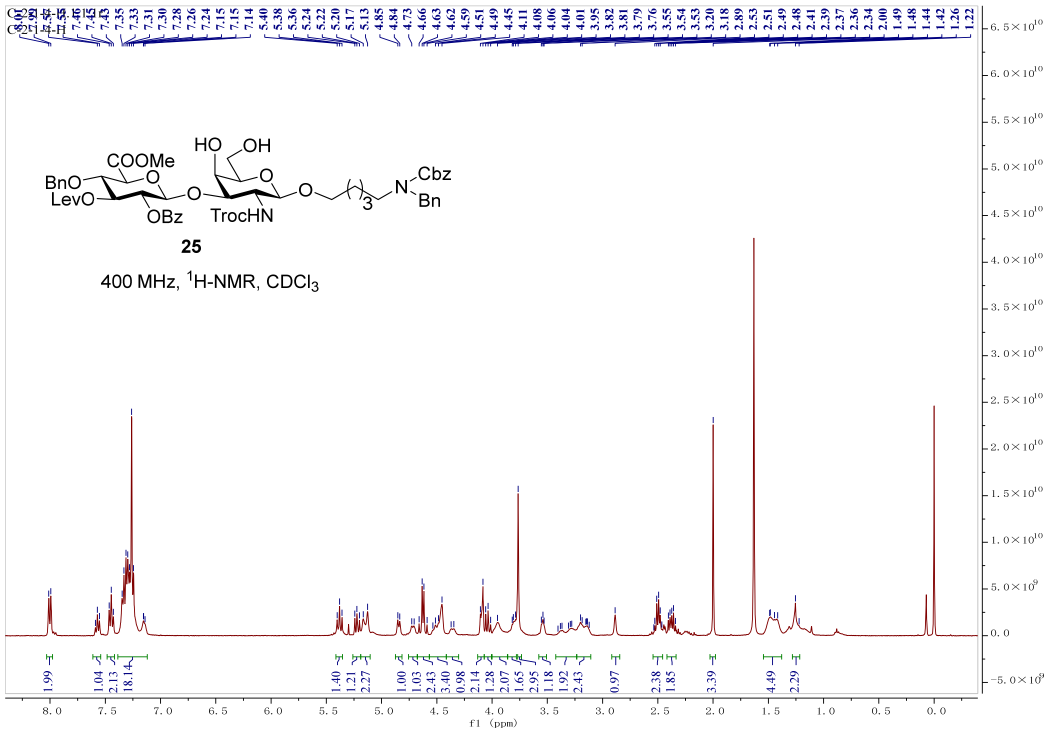

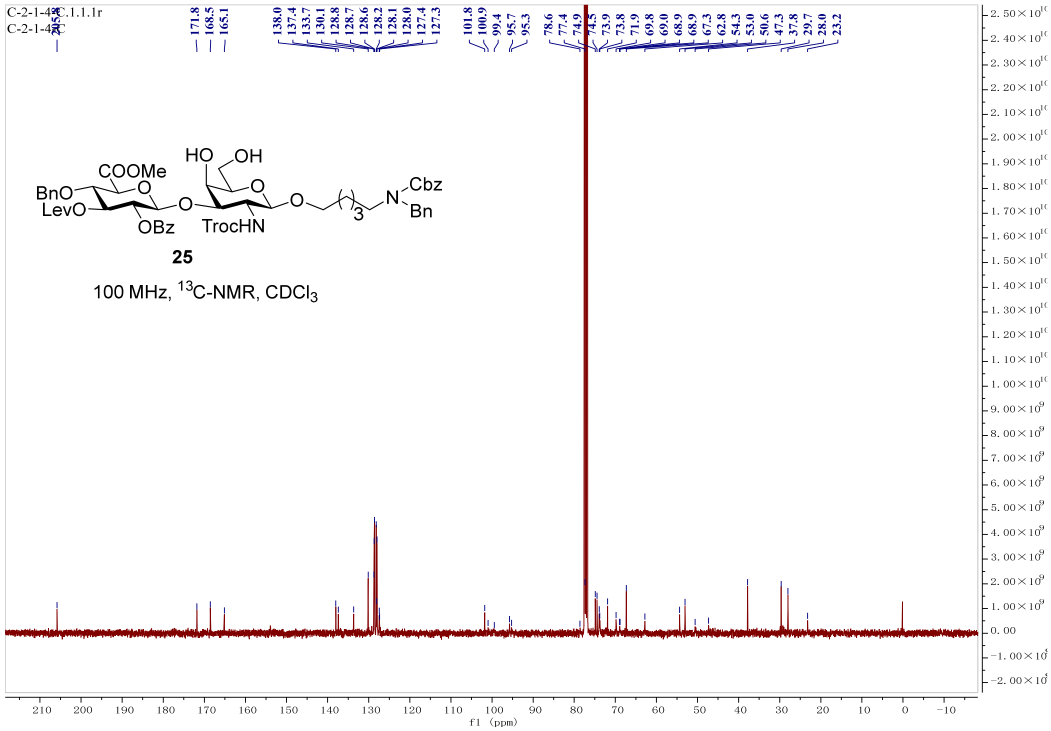

Figure S6. ^{1}H NMR and ^{13}C NMR spectrum of compound **25**.

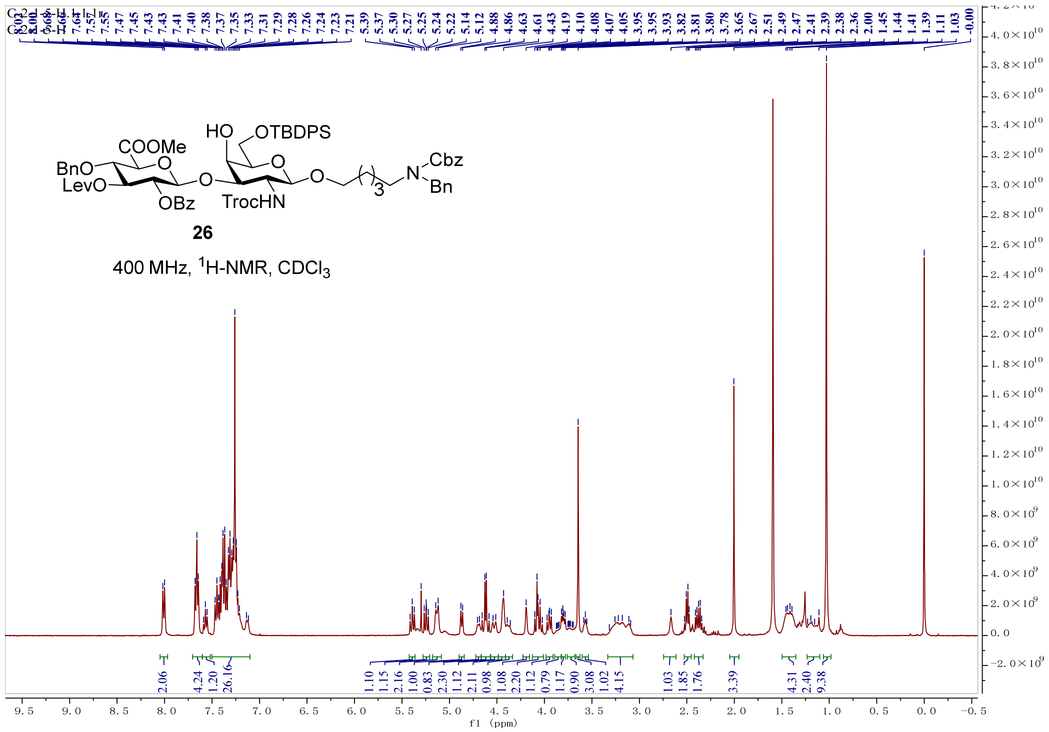

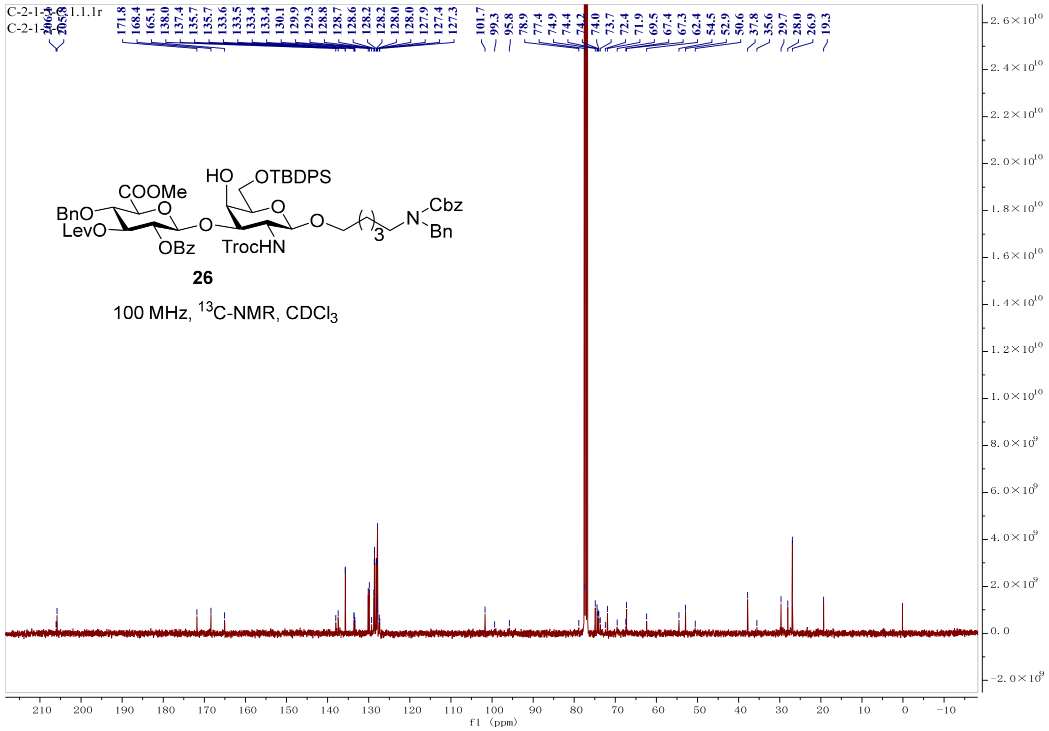

Figure S7. ^{1}H NMR and ^{13}C NMR spectrum of compound **26**.

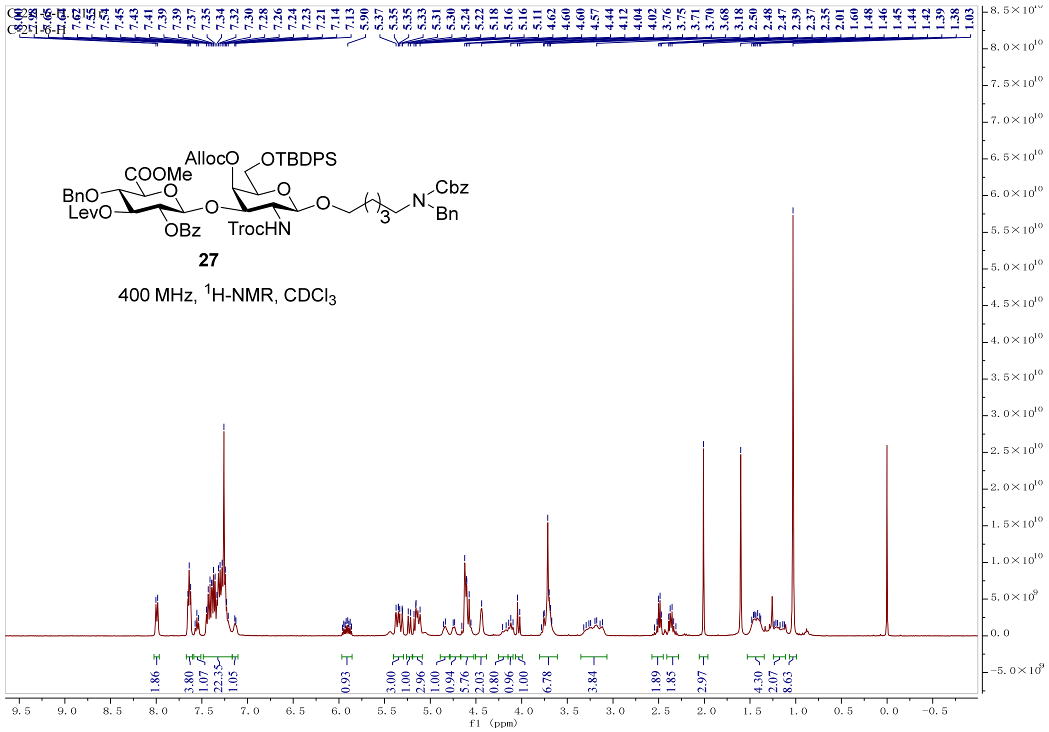

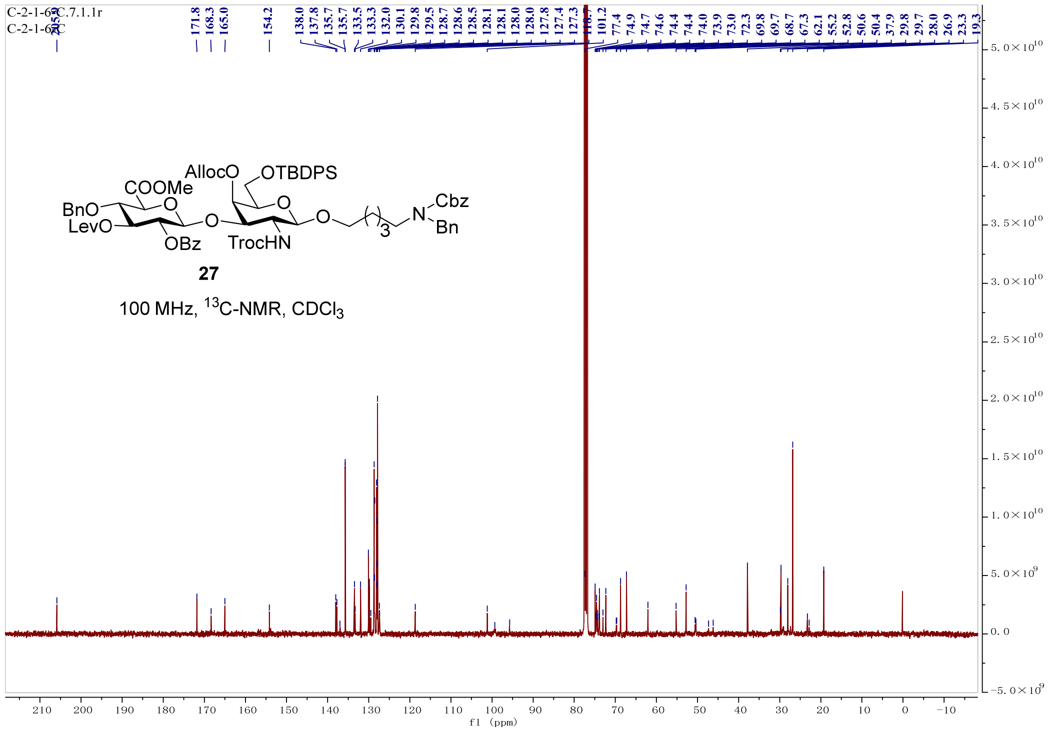

Figure S8. ^{1}H NMR and ^{13}C NMR spectrum of compound **27**.

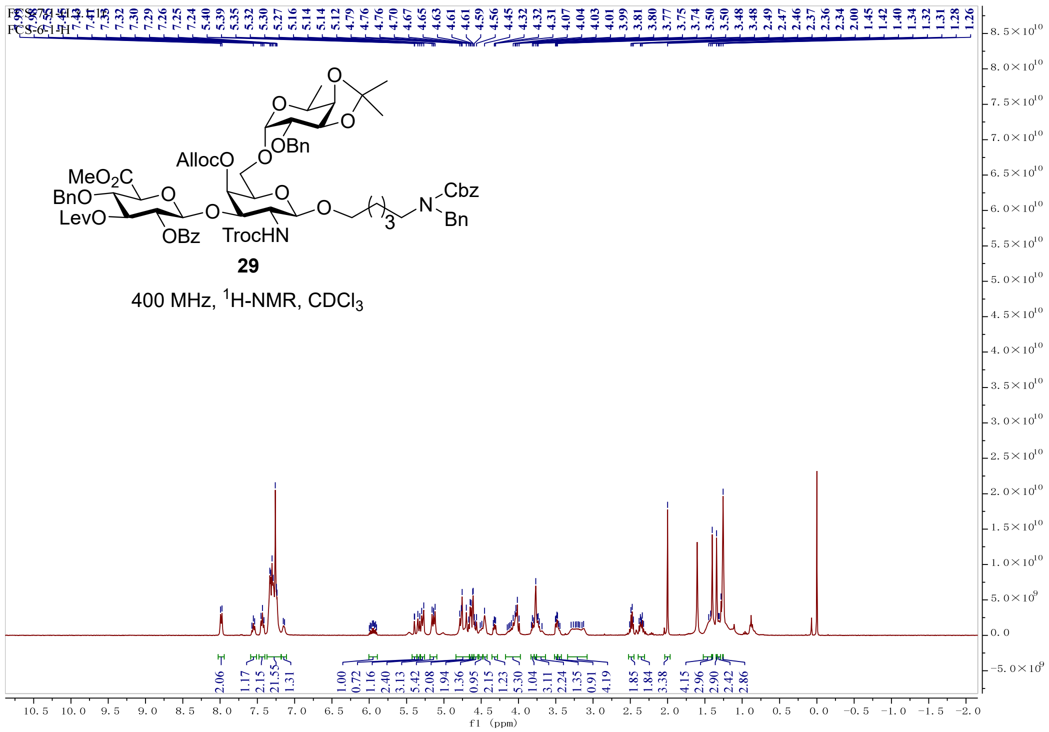

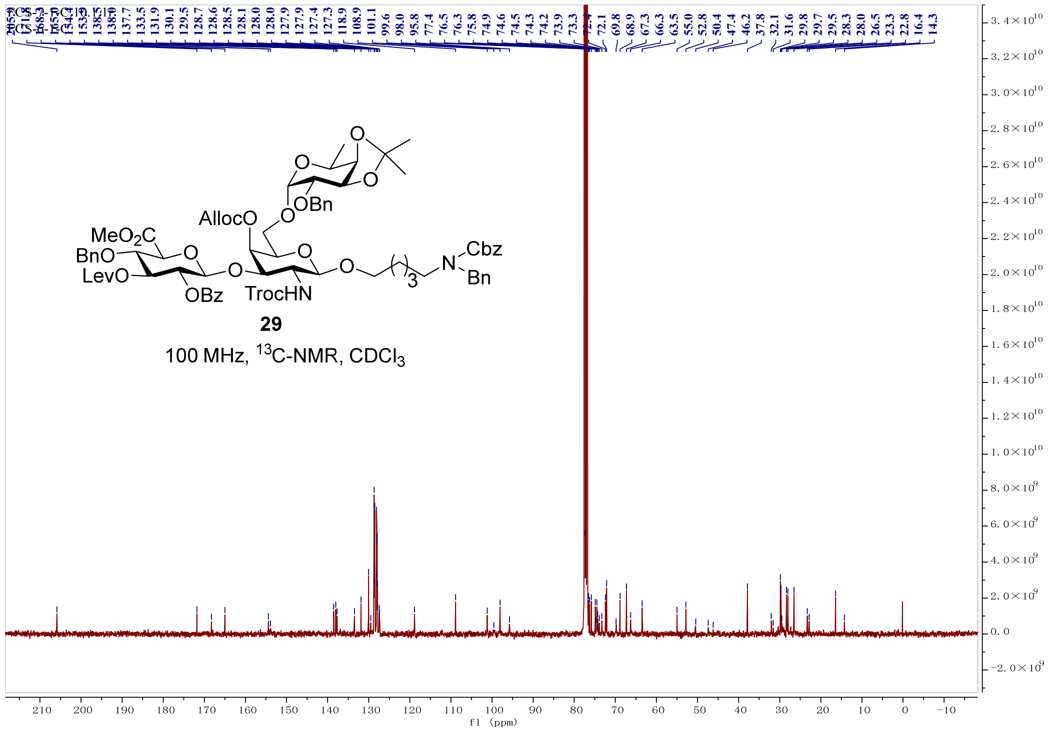

Figure S9. ^{1}H NMR and ^{13}C NMR spectrum of compound **29**.

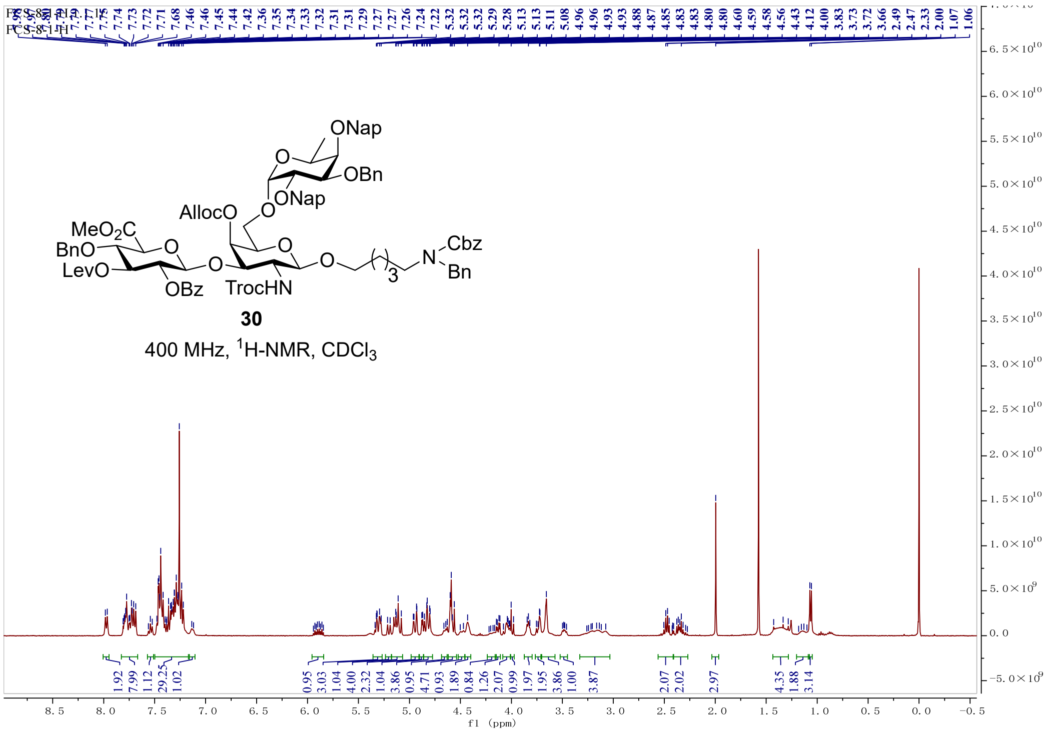

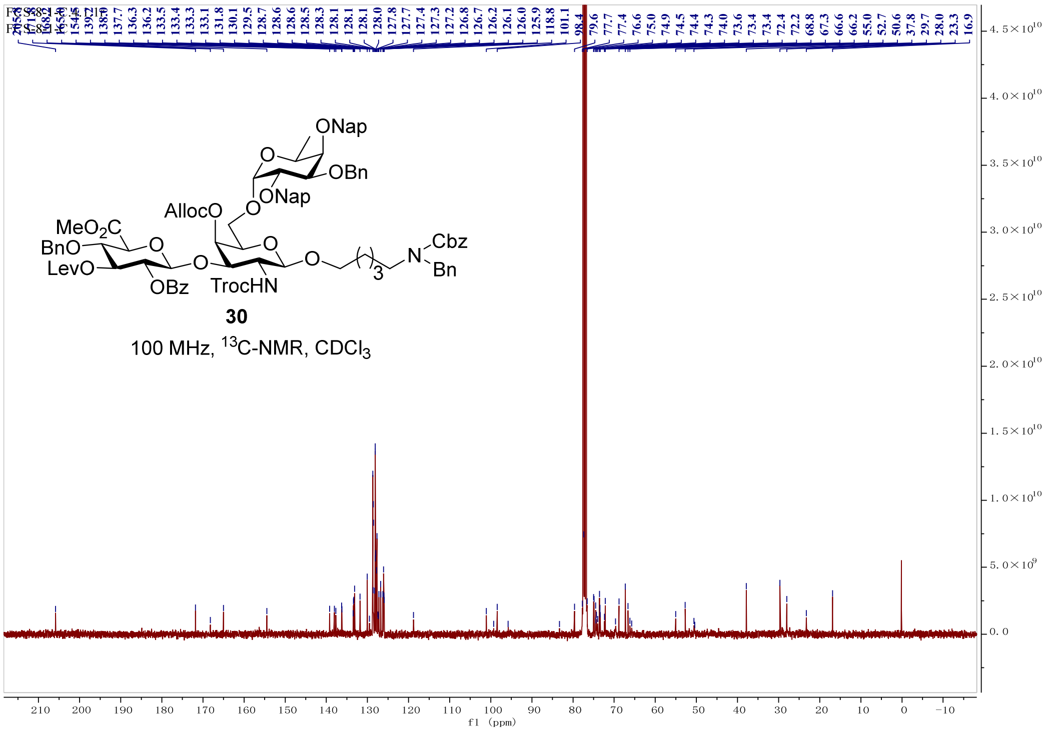

Figure S10. ^1H NMR and ^{13}C NMR spectrum of compound **30**.

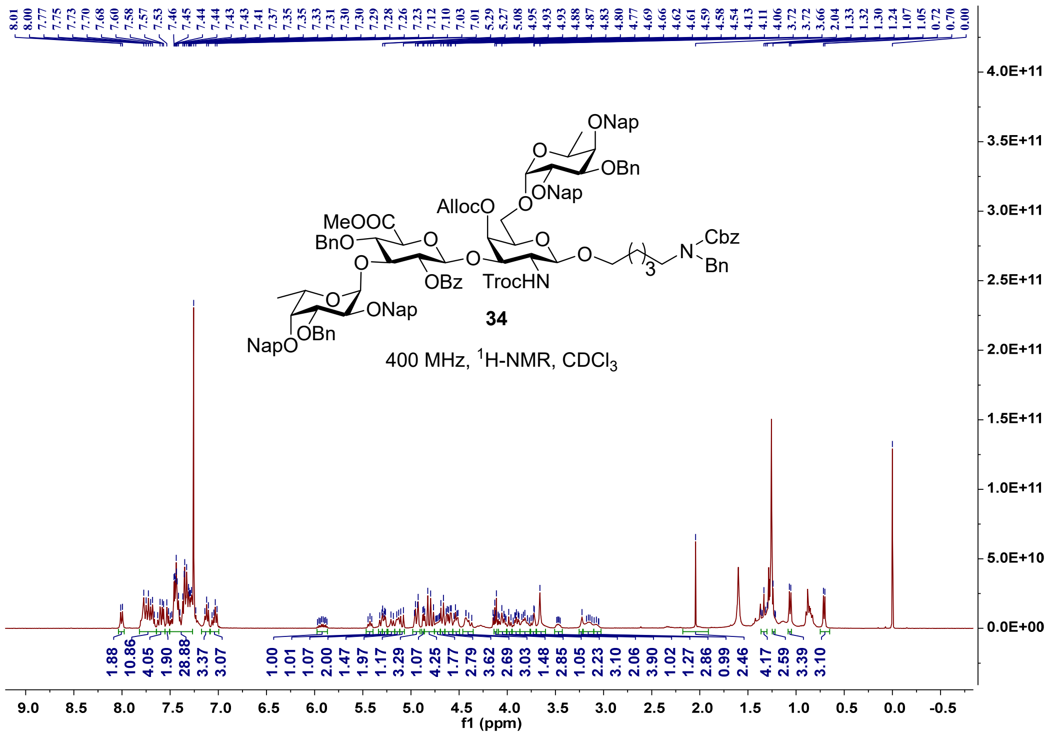

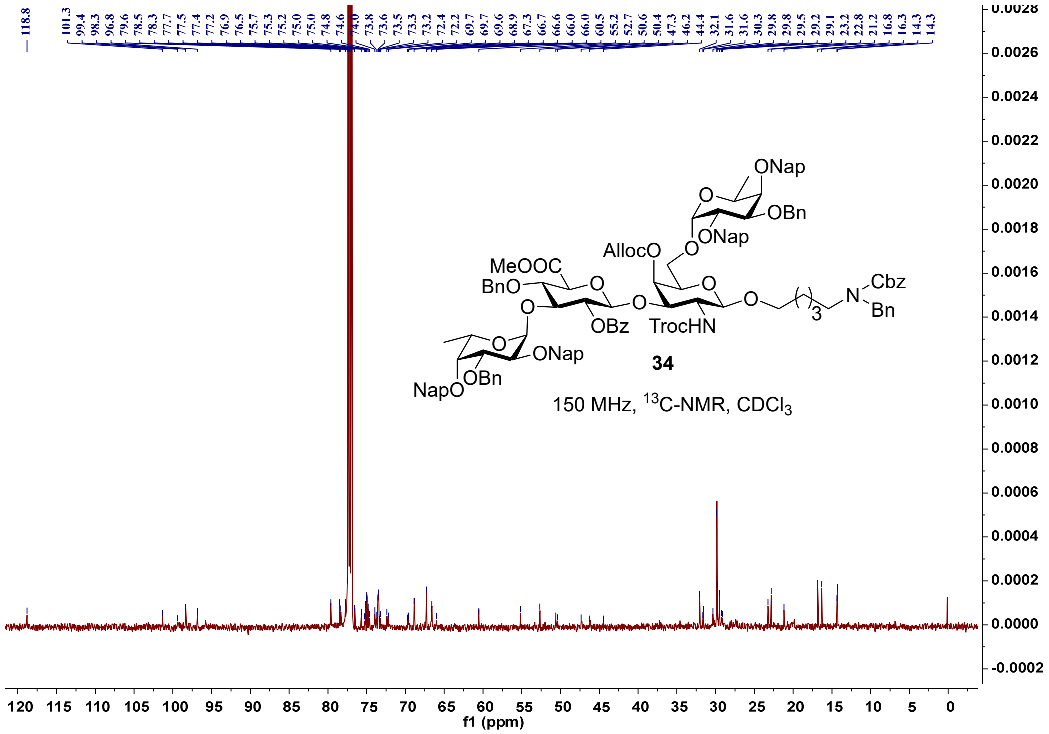

Figure S11. ^{1}H NMR and ^{13}C NMR spectrum of compound **34**.

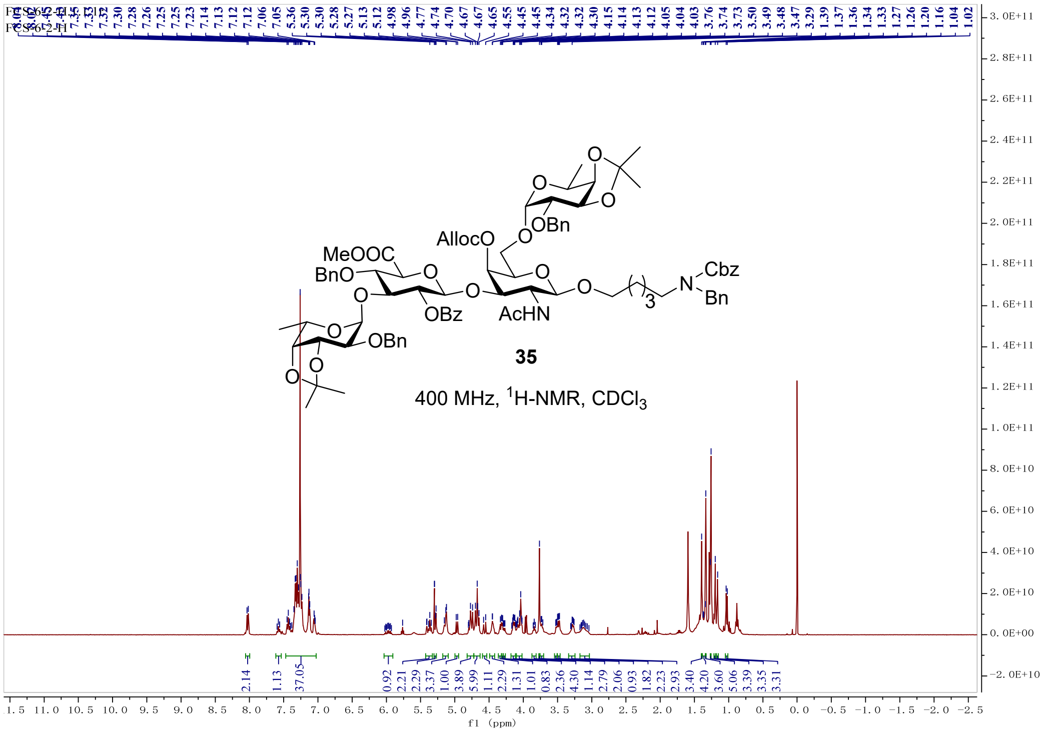

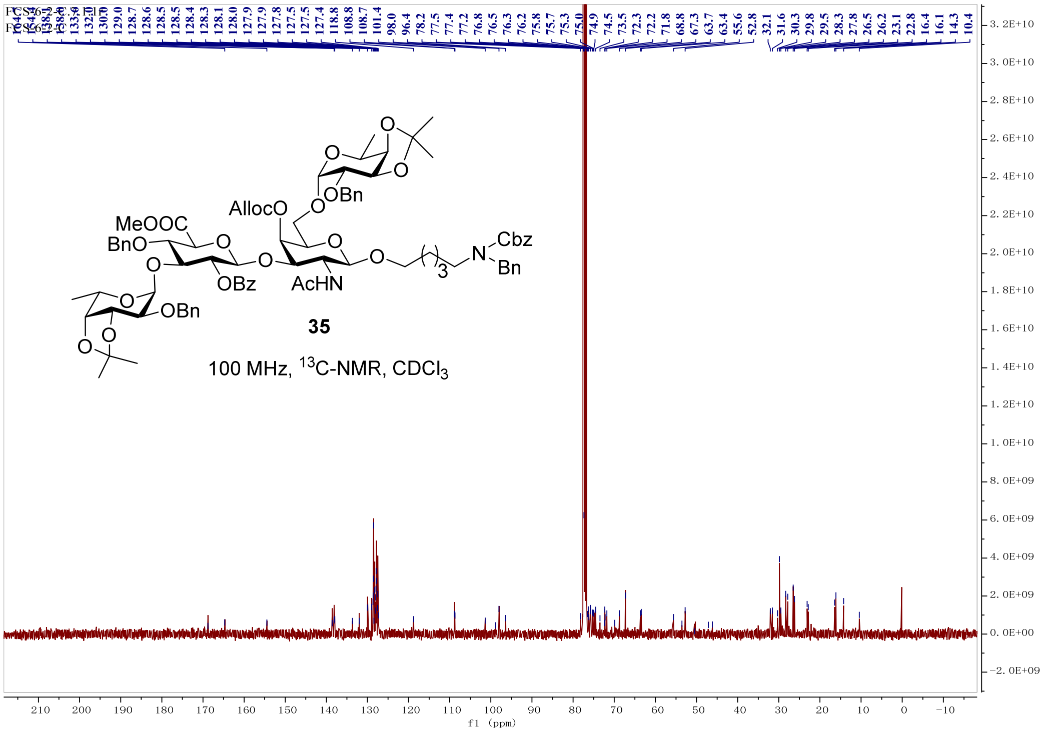

Figure S12. ^{1}H NMR and ^{13}C NMR spectrum of compound **35**.

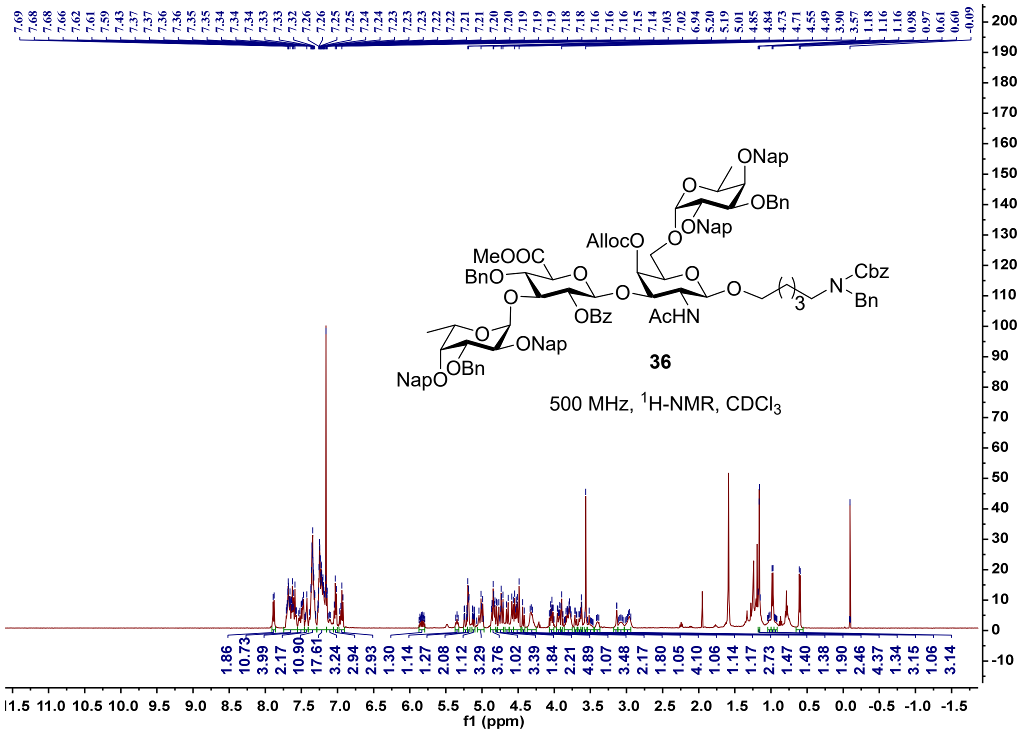

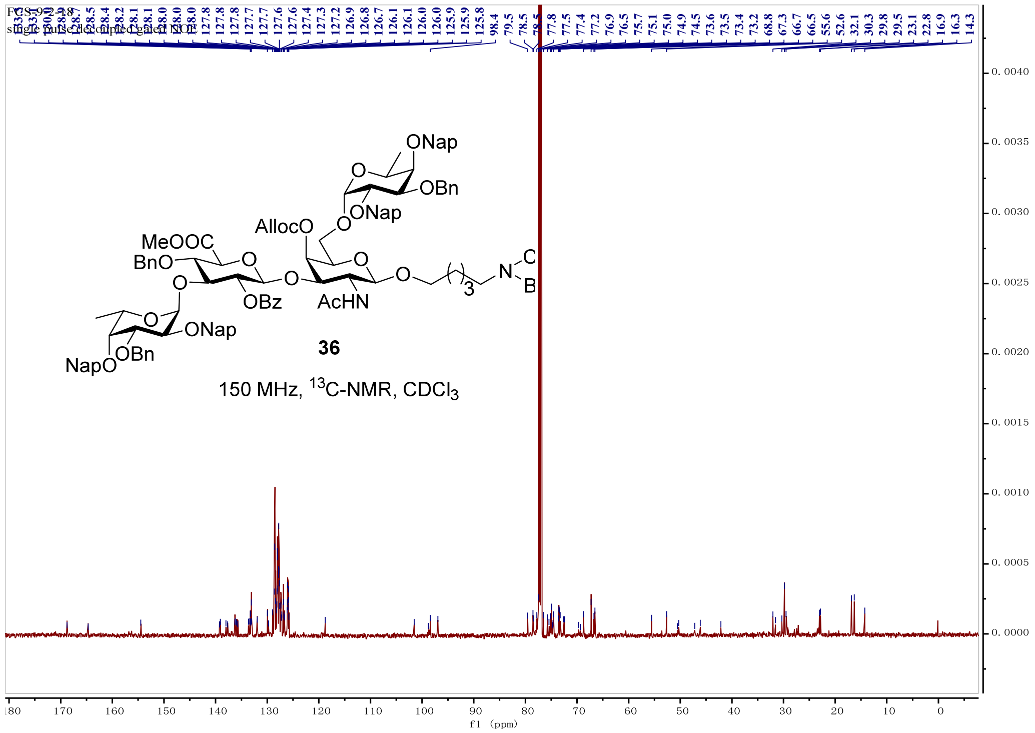

Figure S13. ^{1}H NMR and ^{13}C NMR spectrum of compound **36**.

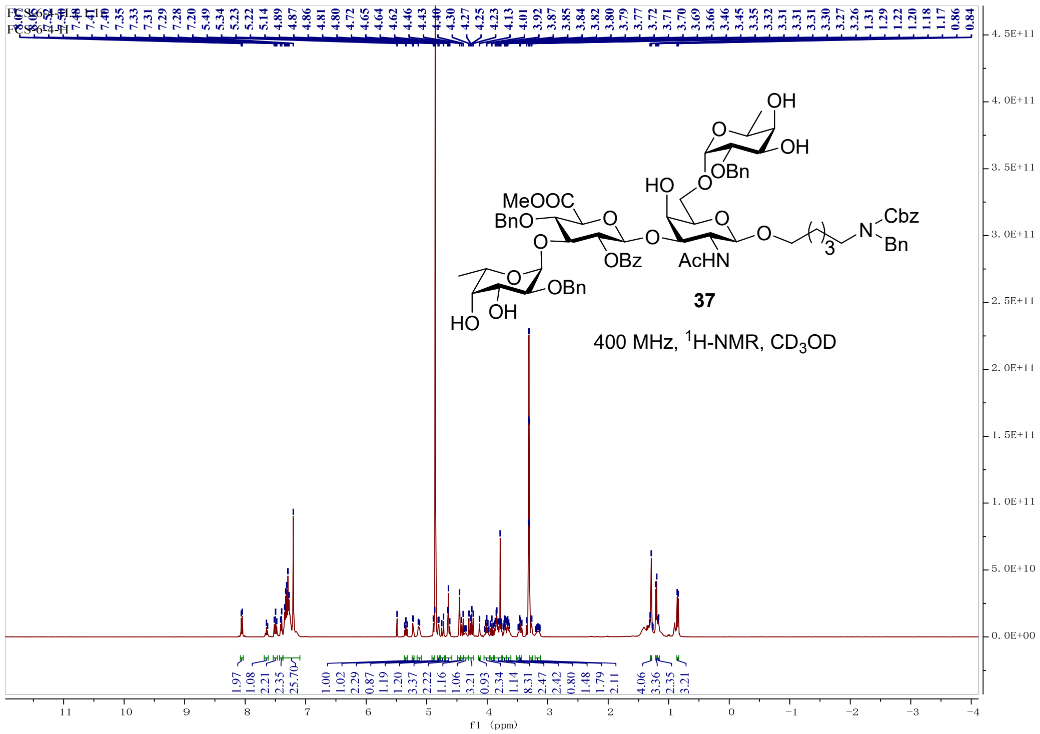

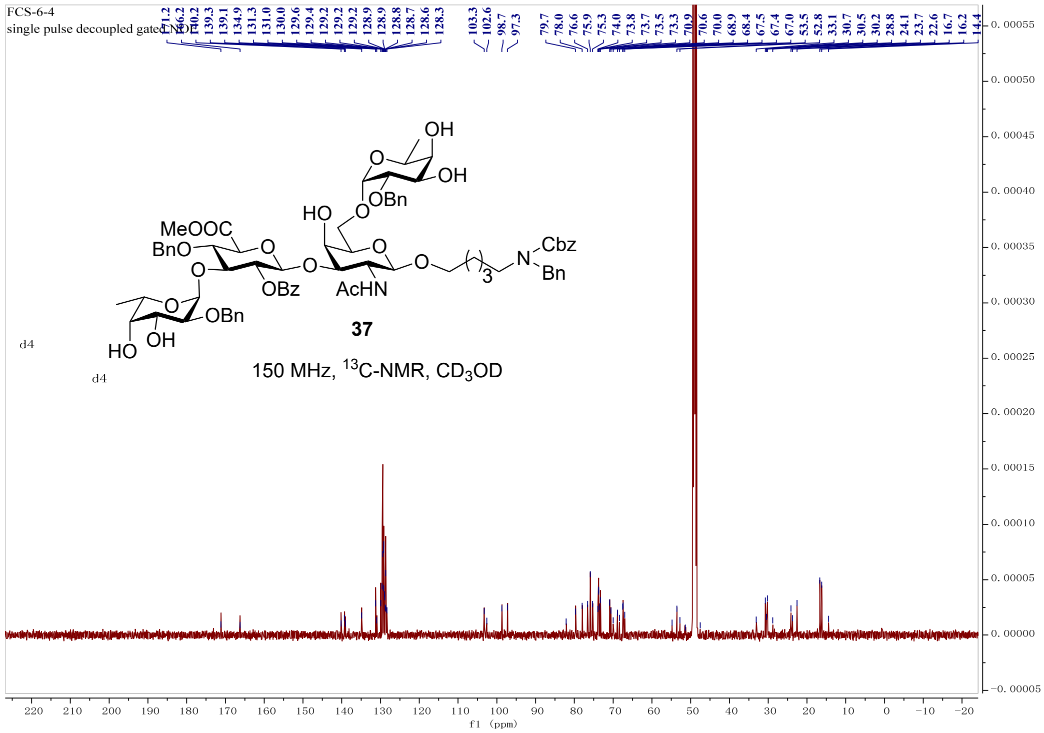

Figure S14. ^{1}H NMR and ^{13}C NMR spectrum of compound **37**.

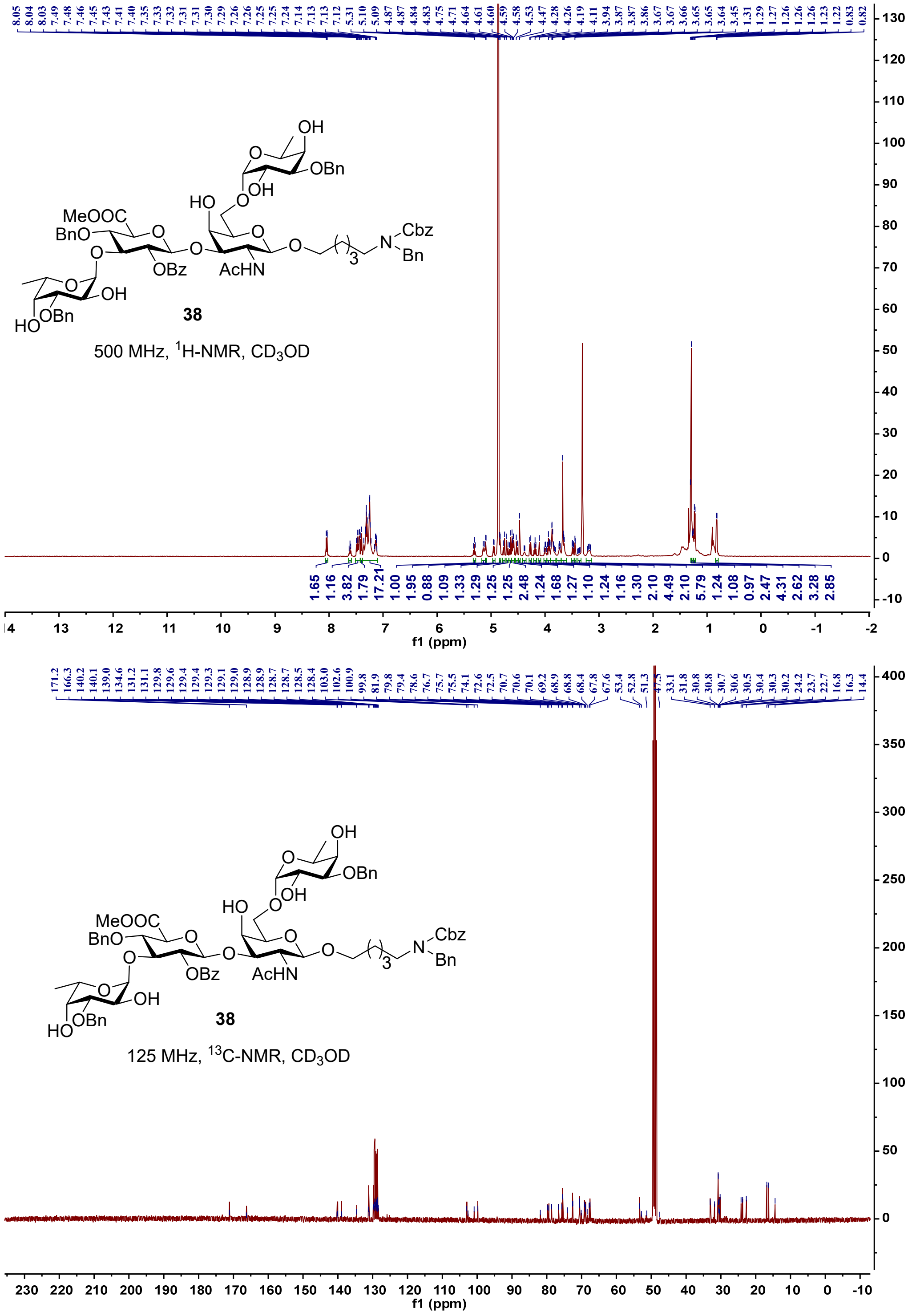

Figure S15. ^{1}H NMR and ^{13}C NMR spectrum of compound **38**.

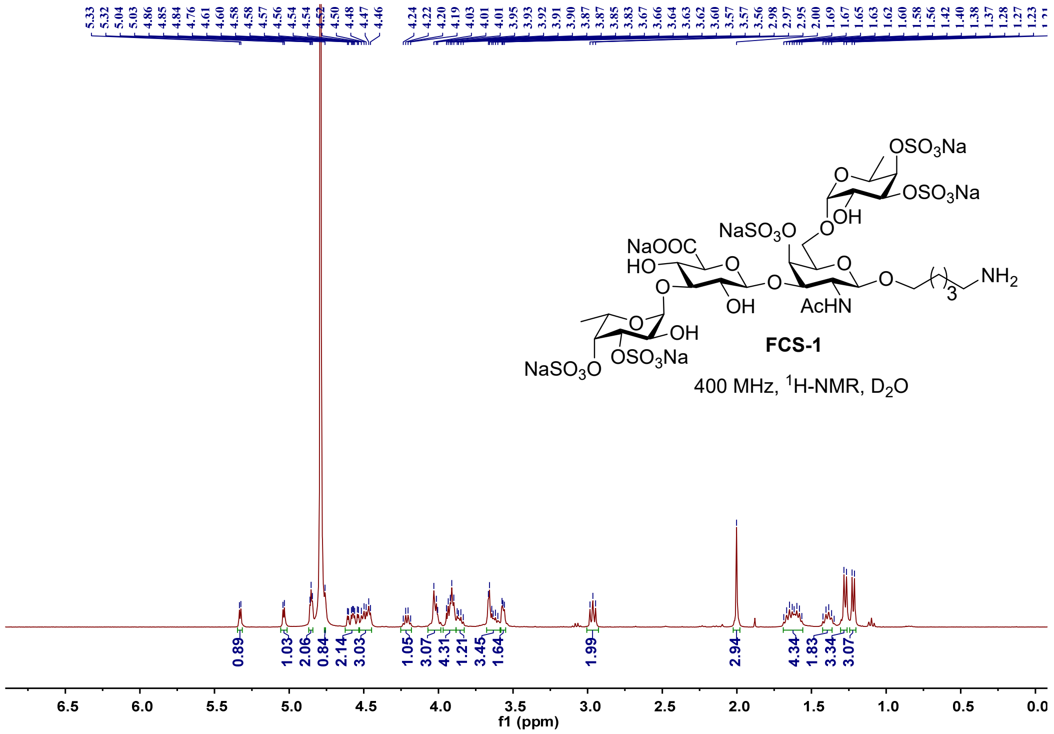

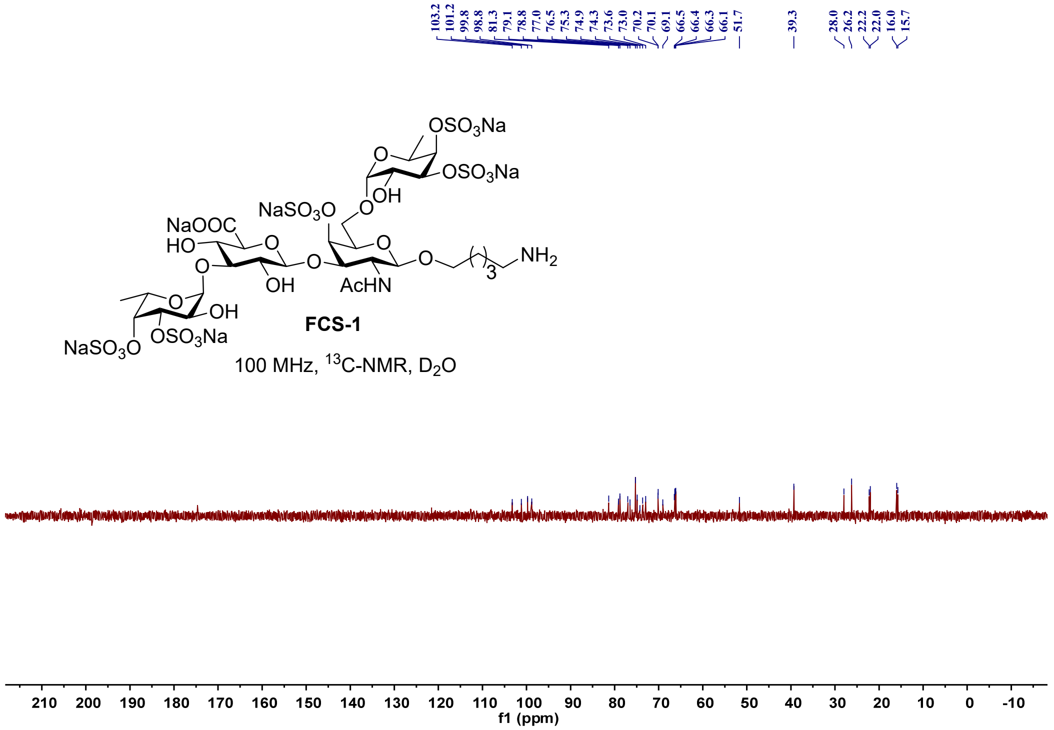

Figure S16. ^{1}H NMR and ^{13}C NMR spectrum of compound **FCS-1**.

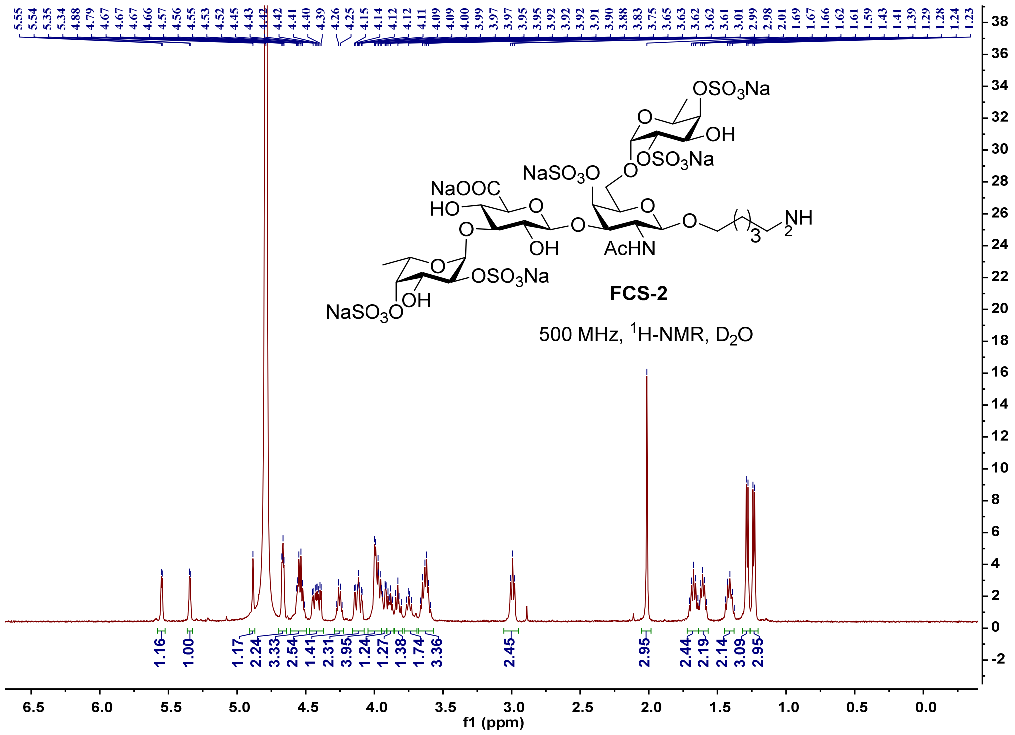

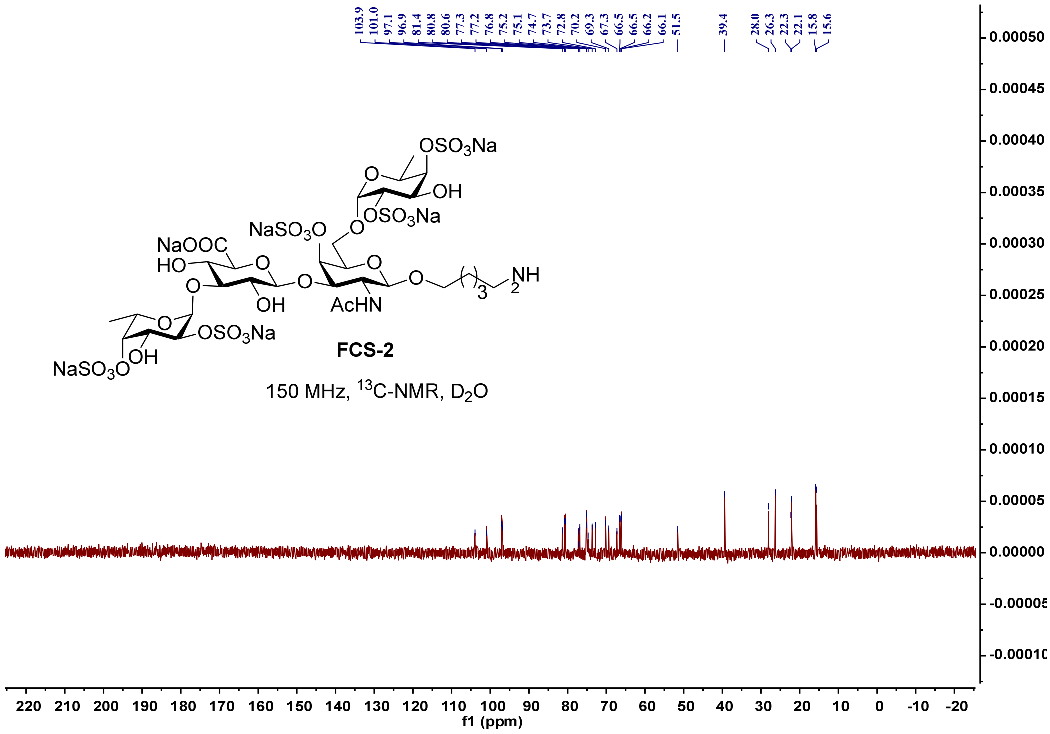

Figure S17. ^{1}H NMR and ^{13}C NMR spectrum of compound **FCS-2**.
